# Therapy-Induced Electrophysiological Changes in Primary Progressive Aphasia: A Preliminary Study

**DOI:** 10.3389/fnhum.2022.766866

**Published:** 2022-03-31

**Authors:** Jara Stalpaert, Sofie Standaert, Lien D’Helft, Marijke Miatton, Anne Sieben, Tim Van Langenhove, Wouter Duyck, Pieter van Mierlo, Miet De Letter

**Affiliations:** ^1^Department of Rehabilitation Sciences, Ghent University, Ghent, Belgium; ^2^Logopediepraktijk Bieke Van Waeyenberghe, Lievegem, Belgium; ^3^Department of Neurology, Ghent University Hospital, Ghent, Belgium; ^4^Department of Experimental Psychology, Ghent University, Ghent, Belgium; ^5^Medical Image and Signal Processing Group, Department of Electronics and Information Systems, Ghent University, Ghent, Belgium

**Keywords:** primary progressive aphasia, event-related potentials, language, neuroplasticity, language therapy, follow-up

## Abstract

**Aims:**

This preliminary study aimed to investigate therapy-induced electrophysiological changes in persons with primary progressive aphasia (PPA). The investigated event-related potential (ERP) components associated with language processing were the mismatch negativity, P300, N400, and P600.

**Methods:**

A linguistic ERP test battery and standardized language assessment were administered in four patients with PPA of which two received speech-language therapy (SLT) and two did not receive therapy. The battery was administered twice with approximately 6 months in between in each patient. The results of the follow-up assessments were compared to the results of the initial assessments.

**Results:**

Although the results of the behavioral language assessment remained relatively stable between the initial and follow-up assessments, changes in the mean amplitudes, onset latencies, and duration of the ERP components were found in the four patients. In the two patients that did not receive SLT, an increased delay in 50% and a decreased mean amplitude in 25% of the measured ERP components were found. The electrophysiological changes found in the patients that received SLT were variable. Interestingly, the mismatch negativity and the N400 effect elicited by the categorical priming paradigm were less delayed and had an increased mean amplitude at the follow-up assessment in the patient with the non-fluent variant who received SLT. In this patient, the P600 component was absent at the initial assessment but present at the follow-up assessment.

**Conclusion:**

Although no clear patterns in electrophysiological changes between patients who received SLT and patients who did not receive SLT were found by our preliminary study, it seems like the SLT induced improvements or compensation mechanisms in some specific language comprehension processes in the patient with the NFV. The results of this study are still preliminary because only four heterogeneous patients were included. Future studies should include larger patient groups of the three clinical variants because the therapy-induced electrophysiological changes might differ depending on the clinical variant and the underlying pathology.

## Introduction

The ability to communicate is one of the most important activities in daily life that can deteriorate in various neurodegenerative diseases. Primary progressive aphasia (PPA) is a group of clinical syndromes in which the language abilities, and consequently verbal communication, progressively deteriorate with relative preservation of the other cognitive functions. Gorno-Tempini et al. (2011) provided a framework for the root diagnosis of PPA and the classification into three variants: the non-fluent or agrammatic variant (NFV), the semantic variant (SV), and the logopenic variant (LV). In the NFV of PPA, the key characteristics are the presence of agrammatism in language production and/or apraxia of speech. The comprehension of syntactically complex sentences could be impaired and single-word comprehension and object knowledge are most frequently spared. The SV is characterized by an impaired confrontation naming and single-word comprehension. In this variant, other supportive features are impaired object knowledge, surface dyslexia or dysgraphia, spared repetition, and a spared speech production. The core features of the LV are impaired single-word retrieval and an impaired repetition of sentences and phrases. Phonologic errors could also be present in this variant but single-word comprehension, object knowledge, motor speech, and grammatical processing are most frequently spared.

Since the onset of PPA tends to be before the age of 65 (Mesulam et al., 2014), the psychosocial and economic impact of this disease on the persons with PPA themselves, but also on their caregivers, families, and the society at large should not be underestimated. At this age, the persons are often still employed, take care of relatives, and have a rich social life, so that PPA likely influences their quality of life, social activities, and full-life participation. Currently, no curative or symptomatic pharmacological treatments are available for PPA. However, the results of non-pharmacological interventions such as support groups and speech-language therapy (SLT) seem promising (Rogalski and Khayum, 2018). Although the evidence for the effectiveness of SLT interventions in PPA is sparse and limited to mostly small participant groups or case studies, positive gains have been reported. Only a few studies have included larger participant groups such as Rogalski et al. (2016) and Henry et al. (2019). Two main types of SLT interventions for persons with PPA can be differentiated namely the impairment-based interventions and the functional communication-based interventions (Volkmer et al., 2020a). The impairment-based interventions consist mainly of word-retrieval therapies in which only the function of word-retrieval is targeted. Consequently, the main outcome of these studies is also most frequently an improved or maintained language function for the trained items. It is, however, less clear if these gains are generalizable to functional communication (Carthery-Goulart et al., 2013; Cadório et al., 2017; Volkmer et al., 2020a). On the other hand, the systematic review of Volkmer et al. (2020b) investigated the research literature on functional communication-based interventions for PPA. These studies investigated mainly the effectiveness of communication skills training for the person with PPA (and their communication partner) and alternative and augmentative communication. All nineteen studies reported improvements in impairment-based and communication-based assessments, social validity judgments, or in the confidence and quality of life of the person with PPA and their communication partner.

To identify which type of SLT serves which patient best in each stage of the disease, it is important to understand how specific improvements in language function and functional communication due to specific SLT interventions relate to mechanisms of reorganization or reactivation in the brain. Therapy-induced brain changes have been investigated by functional magnetic resonance imaging (fMRI) in three case studies with PPA (Dressel et al., 2010; Marcotte and Ansaldo, 2010; Beeson et al., 2011). The results of these three cases, one case of each of the three variants, suggest that the activation might be increased due to SLT in the cortical areas that are typically preserved in the specific variant to compensate for their specific language loss.

Another appropriate technique that could provide insights into the brain changes induced by SLT is the event-related potential (ERP) technique. ERPs are small voltage fluctuations in the electroencephalography that are time-locked to a particular event (Luck, 2014). ERP components that could be associated with language processing are the mismatch negativity (MMN), the P300, the N400, the late positivity complex (LPC) and the P600. The language-related *MMN* and *P300* components are elicited respectively by a pre-attentive and attentive auditory oddball paradigm in which a deviant linguistic stimulus infrequently occurs in a sequence of standard linguistic stimuli such as phonemes, syllables, and words (Aaltonen et al., 1987; Näätänen et al., 1997; Aerts et al., 2013). The MMN is a negative response that occurs between 160 and 220 ms and the P300 is a positive response that occurs at 300–600 ms after the presentation of the deviant stimulus. The MMN is characterized by a frontocentral topographic distribution and the P300 by a parietal distribution. In terms of phoneme perception, the MMN can be associated with phoneme discrimination and the P300 with phoneme categorization (Kok, 2001; Alain and Tremblay, 2007; Luck and Kappenman, 2012; Näätänen et al., 2012). The ERP component associated with semantic processing is the *N400* which is a negative response that starts around 200–300 ms after the onset of a visually or auditorily presented word and peaks around 400 ms. The N400 is characterized by a centroparietal topographic distribution and is often associated with unexpected or incongruous words (Lau et al., 2008; Kutas and Federmeier, 2011; Luck and Kappenman, 2012). At the sentence level, the N400 can be followed by a positive component with a bilateral parietal distribution from approximately 500–1,000 ms after word onset. This component has been referred to as the LPC. This component might reflect the integration of plausibility conflicts (Kim and Osterhout, 2005), more general reanalysis processes (Friederici, 2002), and/or sentence context updating (Kaan et al., 2007). Finally, the ERP component typically observed with syntactically violated sentences is the *P600*. The P600 is a positive response with a parietal topographic distribution that starts around 500 ms after stimulus onset (onset of the word that creates a syntactic processing problem) and lasts several hundred milliseconds (Friederici, 2004; Kielar et al., 2012; Luck and Kappenman, 2012).

Therapy-induced electrophysiological changes have not been investigated in persons with PPA yet (Stalpaert et al., 2020). In persons with aphasia after stroke, on the other hand, the review of Cocquyt et al. (2020) investigated the sensitivity of ERPs to objectify therapy-induced neuroplasticity. The authors concluded that the amplitude and topography parameters of the linguistic ERPs were sensitive to SLT in persons with aphasia after stroke. More specifically, this review suggests that increased amplitudes of early and mid-to-late ERP components and an increase of left-hemispheric lateralization might reflect gains of SLT. This preliminary follow-up study aimed to explore therapy-induced electrophysiological changes in persons with PPA by a linguistic ERP battery. Changes in the amplitude, latency, and topographical distribution of the MMN, P300, N400, and P600 components might be induced by SLT in persons with PPA. These therapy-induced changes might give information about phoneme perception, verbal semantic, and syntactic reanalysis and repair processes in persons with PPA. Importantly, these changes in the various components might be different in the variants of PPA. Since this is the first study to investigate therapy-induced electrophysiological changes in patients with PPA, it is difficult to hypothesize which changes are expected for each component and in each variant.

## Materials and Methods

### Participants

Four patients with a clinical diagnosis of PPA were included in this follow-up study. These patients were recruited at the Department of Neurology of the Ghent University Hospital. Based on the case history, the neurological evaluation, the neuroimaging results, and the evaluation of the language and speech abilities, the clinical diagnosis was made by experienced neurologists (TV and AS) and speech-language pathologists (SLPs; MD and JS). Each patient met the criteria for the root diagnosis of PPA and was classified following the criteria of Gorno-Tempini et al. (2011). The four included patients were evaluated twice with approximately 6 months in between with an electrophysiological test battery, the Dutch version of the Comprehensive Aphasia Test (CAT-NL) (Swinburn et al., 2014), the Diagnostic Instrument for Apraxia of Speech (DIAS) (Feiken and Jonkers, 2012), and the Montreal Cognitive Assessment (MoCA) (Nasreddine et al., 2005). Furthermore, the Dutch Handedness Inventory (DHI; Van Strien, 1992) was administered to each included patient. After the initial assessment, SLT was started in two of the four patients by SLPs (one patient with NFV and one patient with PPA not otherwise specified). In these two patients, SLT was administered twice a week with a therapy session duration of 30 min. The SLT focused on the identified language and speech impairments by the behavioral and electrophysiological assessments. The specific content of the SLT in each patient is specified in the section “Results.” The remaining two patients (one patient with SV and one patient with LV) were not interested to start SLT.

Two age-matched healthy control (HC) groups consisted each of 30 right-handed adults recruited by snowball sampling (five male and five female participants per age decade: 50–59 years, 60–69 years, and 70–79 years). In the first HC group, three ERP paradigms (MMN, P300, and P600) of the electrophysiological test battery were administered. The age of this HC group ranged from 50 to 78 years with a mean of 63.9 years (SD = 8.31) and the education level ranged from 10 to 17 years with a mean of 14 years (SD = 2.05). The scores for the MoCA ranged from 26 to 30 with a mean score of 28.4 (SD = 1). In the second HC group, the remaining two ERP paradigms (categorical priming and semantic anomaly) of the electrophysiological test battery were administered. The age of this HC group ranged from 51 to 84 with a mean of 64.2 years (SD = 9.09) and the education level ranged from 9 to 17 with a mean of 14.33 years (SD = 2.23). The MoCA scores of the HC group ranged from 25 to 30 with a mean score of 27.57 (SD = 1.36). All HCs were right-handed following the DHI, had a (corrected-to-)normal vision, and reported no subjective complaints of hearing loss. None of the HCs had a history of neurological disorders, developmental learning or language disorders, and psychiatric disorders. The native language of all participants was Dutch. The study was performed in accordance with the Declaration of Helsinki and approved by the Ethics Committee of the University Hospital Ghent. Informed consent was obtained from all the participants.

### Experimental Procedure

The electrophysiological test battery consisted of five linguistic ERP paradigms (Cocquyt et al., 2021; Stalpaert et al., 2021a,b; Stalpaert et al., under review)^1^. At the initial evaluation, the five paradigms of the test battery were administered randomly in one session in each patient. For the follow-up evaluations, the order was kept the same in each patient. Both the initial and follow-up electrophysiological evaluations were conducted in the morning or early afternoon in the same dimly illuminated room in the Ghent University Hospital. In each paradigm, the stimuli were presented by E-Prime 3.0 (Psychology Software Tools, Pittsburgh, PA, United States) and the auditory stimuli were delivered at the same comfortable listening level for all participants by ER1 insert earphones (Etymotic Research).

#### Inattentive Oddball Paradigm (Mismatch Negativity)

The auditory oddball paradigm of Stalpaert et al. (2021b) consisted of 600 standard and 150 deviant stimuli with a stimulus onset asynchrony (SOA) of 500 ms. The standard stimulus [bƏ] differed from the deviant stimulus [gƏ] by one phonemic contrast, namely the place of articulation. During this paradigm, the participants were instructed to ignore the stimuli and to focus on a silent movie (Mickey Mouse). The total duration of the experiment was approximately 7 min.

#### Attentive Oddball Paradigm (P300)

The attentive oddball paradigm was also from the study of Stalpaert et al. (2021b). The same stimuli were used as in the inattentive oddball paradigm. The paradigm started with a training block of sixteen standard and four deviant stimuli. Subsequently, the experimental block consisted of 160 standard and 40 deviant stimuli with an SOA of 2,000 ms. During this paradigm, the participants were instructed to press the green button on the Chronos response box (Psychology Software Tools, Pittsburgh, PA, United States) when they heard the deviant stimulus. A white fixation cross was presented on a black background to focus their attention and to reduce vertical and horizontal eye movements. The total duration of the experiment was approximately 8 min.

#### Categorical Word Priming Paradigm (N400)

The categorical word priming paradigm of Cocquyt et al. (2021) consisted of 120 Dutch prime-target pairs that were auditorily presented to the participants. Half of the word pairs were categorically related (e.g., rugby – basketball) while the other half were members of a different category (e.g., potato – photographer). None of the word pairs was associatively related. The paradigm started with a practice block followed by seven experimental blocks that were presented in random order. Between blocks, participants could take a break as long as they wanted. The inter-stimulus interval (ISI) between the targets and primes varied between 830 and 1,520 ms and the SOA was 1,800 ms. The interval between the trials was 2,500 ms. The participants were instructed to judge whether the word pairs belonged to the same or a different category by a button press response. To avoid contamination of response-related potentials, this button press response was delayed (Van Vliet et al., 2014). The HC group had to press their left or right index finger on a green or red button for the categorically related and unrelated pairs respectively. Due to motor deficits in some patients, the patients had to press the green and red buttons on the Chronos response box with their hand of preference. The place of the green and red buttons was not randomized. During the paradigm, a white fixation cross was presented on a black background to focus their attention and to reduce vertical and horizontal eye movements. The total duration of the experiment without breaks was 17 min.

#### Semantic Anomaly Paradigm at the Sentence Level (N400)

The semantic anomaly paradigm at the sentence level of Stalpaert et al. (2021a) consisted of 120 visually presented Dutch sentences of which half were semantically and syntactically correct sentences (e.g., “The girl tied the dog to the tree.”) and the other half contained a semantic violation at the end of the sentence (e.g., “The bucket is full of fever.”). The final words (= target words) of the correct and incorrect sentences were closely matched in orthographic length, amount of orthographic neighbors (Marian et al., 2012), word frequency (Keuleers et al., 2010), concreteness (Brysbaert et al., 2014), age of acquisition, valence, arousal, and dominance (Moors et al., 2013). A cloze probability test revealed that the sentences in both conditions were equally constraining (cloze probability correct: 54.6%; incorrect: 60.3%). The paradigm started with a practice block followed by seven experimental blocks in which the stimuli were presented randomly. The participants could take a break as long as they wanted between the blocks. A white fixation cross on a black background was presented for 1.5 s at the start of each trial to warn the participants that they had to fixate their eyes on the center of the screen. Subsequently, the sentences were presented word by word in white, lowercase letters at the center of the screen on a black background. Each word was presented with a duration of 500 ms followed by a blank (black) screen for 500 ms. After the presentation of the complete sentence, a blank screen was presented during 1 s followed by the word “press.” At the presentation of “press,” the participants had to judge whether the sentence was semantically acceptable or not by a button press response. This button press response was delayed and measured in the same manner as in the categorical word priming paradigm. The total duration of the experiment without breaks was approximately 26 min.

#### Word-Order Violations Paradigm (P600)

The word-order violations paradigm of Stalpaert et al. (see text footnote 1) consisted of 60 Dutch sentences. Half of these sentences were syntactically and semantically correct and the other half contained a word-order violation (e.g., “The orchestra played to an empty almost hall in Brussels.”). The correct sentences were not the counterparts of the sentences with the word-order violation. An adverb-adjective-noun or an adjective-adverb-noun construction was present in the middle of respectively the correct and incorrect sentences. The target word was the adjective in the correct and the adverb in the incorrect sentences. A practice block of six sentences was followed by four experimental blocks in which the sentences were presented randomly to the participants. The participants could take a break as long as they wanted between the blocks. The presentation of the sentences and the button press response was the same as in the semantic anomaly paradigm. The total duration of the experiment was approximately 17 min without breaks.

### EEG Recording

Continuous EEG was recorded at 32 electrode sites in the HC groups and 126 electrode sites in the patients with PPA, both using an EasyCap electrode cap (Brain Products, Munich, Germany). The same subsets of electrodes were analyzed in both the HC and PPA group. FCz was used as the online reference electrode and AFz as the ground electrode. The electrode impedances were kept below 10 kΩ by using an abrasive electrolyte gel (Abralyt, 2000, EasyCap). Data were collected with a BrainVision BrainAmp amplifier (Brain Products, Munich, Germany) and were continuously digitized with a sampling frequency of 500 Hz. BrainVision Recorder was used as recording software (Brain Products, Munich, Germany).

### Event-Related Potential Data Analysis

Offline EEG analysis was performed with the BrainVision Analyzer 2 software (Brain Products, Munich, Germany). First, the practice blocks were removed and bad electrode channels were disabled. The online reference electrodes and the electrodes of interest were not disabled in any of the participants. Subsequently, continuous EEG recordings were band-pass filtered using an infinite impulse response filter (zero phase shift Butterworth filter) with half-amplitude cut-off frequencies of 0.3 and 30 Hz and a slope of 12 dB/octave. A notch filter was applied at 50 Hz. The independent component analysis (ICA) was used to remove eye blinks and horizontal eye movements. Following the ICA, the disabled channels were interpolated and the average of the mastoid electrodes (TP9 and TP10) was applied as a new reference to the data. Next, the responses elicited by the two conditions in each paradigm were segmented separately. The conditions and the time window of the epochs of each paradigm are presented in Table 1. Baseline correction was applied after the segmentation using the pre-stimulus windows presented in Table 1. Subsequently, artifact rejection was applied automatically with the following settings: maximum gradient criterion of 75 μV, minimal-maximal amplitude criterion of 100 μV, maximum difference criterion of 150 μV, and low activity criterion of 0.5 μV during 100 ms. Following the artifact rejection, the responses elicited by the two conditions in each paradigm were averaged separately. At least 75% of the trials in the HCs and 50% of the trials in the patients had to be included in the averaged ERPs. The button press accuracies were also collected but both the trials with a correct and incorrect button press response were included in the analysis. Finally, difference waves for each paradigm were computed by subtracting the response elicited by one condition from the response elicited by the other condition.

**TABLE 1:** The two conditions, the time window of the epoch, and the time windows of the mean amplitude and onset latency for each paradigm.

Paradigm	Conditions	Epoch	Time windows mean amplitude	Time window onset latency
MMN	1. standard 2. deviant	−100–500 ms: onset stimulus	150–250 ms 250–350 ms 350–450 ms	150–450 ms
P300	1. standard 2. deviant	−300–1,200 ms: onset stimulus	350–550 ms 550–750 ms 750–950 ms	350–950 ms
Categorical priming (N400)	1. same category 2. different category	−300–1200 ms: onset target word	300–500 ms 500–700 ms 700–900 ms	300–900 ms
Semantic anomaly (N400 and LPC)	1. correct sentence 2. incorrect sentence	−300–1,200 ms: onset target word	N400: 300–500 ms 500–700 ms 700 – 900 ms LPC: 500–700 ms 700–900 ms 900–1,100 ms	N400: 300–900 ms LPC: 500–1,100 ms
P600	1. correct sentence 2. incorrect sentence	−300–1,500 ms: onset target word	500–750 ms 750–1000 ms 1000–1,250 ms	500–1,250 ms

*MMN, mismatch negativity; LPC, late positivity complex; ms, milliseconds.*

Two main outcome variables were extracted from the difference waves of each paradigm namely the mean amplitude and the onset latency. Concerning the mean amplitude, the average voltage over three specified measurement windows was computed (Luck, 2014). These time windows were defined based on previous research, visual inspection of the topographic distribution of the components in the HC group, and the hypothesis that the components might be delayed or prolonged in patients with PPA. For the onset latency, the negative area for the MMN and the N400 or the positive area for the P300, LPC, and P600 under the difference waveforms within a specific time window was computed. The time point that divided the first 25% of the area from the last 75% of the area was defined as the onset latency, or 25% fractional area latency (Luck, 2014). The specific time windows for both the mean amplitude and onset latency of each paradigm can be found in Table 1. The mean of the mean amplitudes and onset latencies was calculated for different electrode subsets namely the frontal (F3, F4, Fz), central (C3, C4, Cz), parietal (P3, P4, Pz), left (F3, C3, P3), midline (Fz, Cz, Pz), and right electrode sites (F4, C4, P4).

### Interpretation Event-Related Potentials

In clinical practice, ERP components are most frequently assessed by visual inspection. Although statistical methods for the interpretation of ERPs at the single-subject have been investigated as well, contradictory results have been reported for the various methods. The combination of two or more methods is proposed to be the most sufficient to decide on the presence or absence of an ERP component at the single-subject level (Kallionpää et al., 2019). In this study, we combined two methods that are easily applicable in clinical practice to interpret the ERP results of each patient.

First, the ERP results of the initial and follow-up assessments in each patient were visually inspected. The results of each ERP paradigm were plotted on figures and two raters evaluated independently whether the ERP component of interest was present or not. The figures were presented in a random order to the two raters. If both raters agreed on the presence, we defined the component to be present (Kallionpää et al., 2019). Furthermore, each rater also evaluated if the component was delayed, prolonged, or showed an accelerated decay in comparison to the HC group. The ERP results of the follow-up assessments were also compared to the results of the initial assessments by visual inspection. After visual inspection, the results of each paradigm in each patient were compared to the results of the HC group and the results of the initial assessments were compared to the follow-up assessments by *Z*-scores (Crawford et al., 2006). The means and standard deviations of the mean amplitudes and the onset latencies at the various electrode subsets of the HC group were calculated and based on these results the raw values of the patients were converted into standardized *Z*-scores. The raw values and the *Z*-scores can be found in the Supplementary Material. Since we do not know which *Z*-scores are clinically significant, we consider *Z*-scores ≥ 1.28 (α ≤ 0.10) as impaired in comparison to the HC group.

## Results

The demographic characteristics and the button press accuracies during the ERP paradigms of each included patient are presented in Table 2. In the section “Results,” each case is described by summarizing (1) the diagnostic process including the results of the neurological, imaging, language, and speech examinations, (2) the results of the neurological, language, and speech follow-up assessments, and (3) the electrophysiological results of the initial and follow-up assessments. Subsequently, the results of the patients who received SLT were compared to the results of the patients who did not receive SLT. In the Supplementary Material, the raw values and the corresponding Z-scores of the mean amplitudes and onset latencies of each component, the results of the CAT-NL, and the results of the DIAS can be found. The figures of the electrophysiological results of each paradigm in each patient can also be found in the Supplementary Material.

**TABLE 2:** Demographic characteristics and button press accuracies of the included patients with PPA.

Patient	NFV		PPA-NOS		LV		SV	
Sex	male		female		male		male	
Handedness	right		right		left		right	
Education (years)	12		12		14		12	
Age at onset (years; months)	64;4		68		64		70	
Assessment time	T1	T2	T1	T2	T1	T2	T1	T2
Age at assessment (years; months)	65;9	66;2	70	70;8	68;3	68;9	74;3	74;9
Duration between T1 and T2 (in months)	5		8		6		6	
Speech-language therapy	yes		yes		no		no	
MoCA total score (30)	25	25	23	22	27	27	27	29
**Button press accuracies (% correct)**								
P300	100	100	80	83.5	84.5	24	97.5	99.5
N400 (priming)	92.5	96.7	92.5	90.8	89.2	92.5	90.8	92.5
N400 (anomaly)	96.7	96.7	89.2	91.7	95.8	96.7	–	–
P600	93.3	81.7	41.7	53.3	98.3	95	–	–

*NFV, non-fluent variant; PPA-NOS, not otherwise specified; LV, logopenic variant; SV, semantic variant; MoCA, Montreal Cognitive Assessment; T1, initial assessment; T2, follow-up assessment.*

### Case Descriptions

#### Patient With NFV (Speech-Language Therapy)

This patient was referred to the Department of Neurology of the Ghent University Hospital because of progressive unintelligible speech and difficulties with balance for approximately 1 year. The initial clinical neurological examination showed a right-lateralized, mild extrapyramidal syndrome, besides the language and speech impairments. MRI was unremarkable and FDG-PET showed a mild hypometabolism in the right precuneus of unknown significance. CSF biomarkers and genetic mutations were not investigated in this patient. At T1 (test moment 1), 3 months after the initial neurological examination, the language and speech abilities were evaluated by the CAT-NL, the DIAS, and the electrophysiological test battery. Although no deficits were found in the CAT-NL, his spontaneous speech showed a slow speech rate, a reduction of spontaneous speech, short sentences, word-finding difficulties, and sporadically telegraphic speech. His spontaneous speech also showed the presence of AOS which was confirmed by the DIAS. Based on these results, the clinical diagnosis of the NFV was made (Gorno-Tempini et al., 2011) and SLT was initiated twice a week. The therapy focused on training phonological input and output, syntactic comprehension and production, and motor speech on the level of words and sentences. At T2 (5 months after T1), the CAT-NL still did not show any deficits except for the reading words subtest. In the DIAS, no critical differences between the scores of the subtests on T1 and T2 were found. However, the DIAS showed the presence of five characteristics of AOS which was two more than at T1. The patient reported that he thought that language and speech difficulties did not increase between T1 and T2. Differences in the presence, mean amplitudes, and onset latencies of the ERP components were found by four of the five paradigms at T1 and the five paradigms at T2 in comparison to the HC group. Furthermore, differences were found between the results of each paradigm at T1 and T2. The electrophysiological results are presented in Table 3.

**TABLE 3:** The electrophysiological results of each ERP component in the patients with NFV and PPA-NOS who received SLT at the initial assessment and the follow-up assessment.

	NFV		PPA-NOS	
	T1 vs. HCs	T2 vs. T1	T1 vs. HCs	T2 vs. T1
MMN (inattentive oddball paradigm)	Present F, L: delayed, MA ↑ F: accelerated decay	No delay and decay C, M, R: MA ↑	Absent	Absent
P300 (attentive oddball paradigm)	Present accelerated decay	C: delayed F, C, L, M, R: MA ↓	Absent	Absent
N400 (categorical priming paradigm)	Present	Earlier onset latency F, C, P, L, M, R: MA ↑	Present prolonged	Not prolonged F, P, L, M: delayed
N400 (semantic anomaly paradigm)	Present P: prolonged P: MA ↑ F: MA ↓	Absent at F, C, L, M, R	Present prolonged C: delayed	No delay C, L: prolonged
LPC (semantic anomaly paradigm)	Present prolonged C, P, M, R: delayed	No delay	Present F, C, P, L, M, R: delayed	Absent
P600 (grammaticality judgment paradigm)	Absent	Present at P	Present prolonged	Not prolonged P, L, M: delayed

*The differences in mean amplitude, onset latency, and topographic distribution between the initial assessment and the healthy control group are presented in the column “T1 vs. HCs.” In the column “T2 vs. T1,” the differences in mean amplitude, onset latency, and topographic distribution between the initial and the follow-up assessments are presented. NFV, non-fluent variant; PPA-NOS, primary progressive aphasia not otherwise specified; T1, initial assessment; T2, follow-up assessment; HC, healthy control; MMN, mismatch negativity; LPC, late positivity complex; F, frontal electrode sites; C, central electrode sites; P, parietal electrode sites; L, left electrode sites; M, midline electrode sites; R, right electrode sites; MA ↓, reduced mean amplitude; MA ↑, increased mean amplitude.*

#### Patient With PPA-NOS (Not Otherwise Specified; Speech-Language Therapy)

This patient was referred to the Department of Neurology of the Ghent University Hospital because of progressive difficulties with forming sentences and speech problems. MRI showed mild atrophy at the left frontal and temporal operculum and FDG-PET showed a left fronto-insular hypometabolism. CSF biomarkers and genetic mutations were not investigated in this patient. One month after the initial neurological examination (T1), the language and speech abilities were evaluated by the CAT-NL, the DIAS, and the electrophysiological test battery. Although the results of the CAT-NL only showed deficits in the fluency and sentence repetition subtests, paragrammatic errors, interrupted sentences, sporadic telegraphic speech, a reduction in spontaneous speech, word-finding difficulties, and phonological and semantic paraphasias were present in her spontaneous speech. Also, the DIAS did not conclude the presence of AOS because only two characteristics of AOS were present in this assessment. However, she performed worse on the alternating sequences than on the sequential sequences and articulatory groping was noticed during the DIAS and spontaneous speech which might suggest the discrete presence of AOS. Based on these results, she was diagnosed with PPA-NOS (not otherwise specified) since she met one core feature and two supportive features of the NFV and two core features and three supportive features of the LV (Gorno-Tempini et al., 2011).

Due to COVID-19 restrictions, she initiated SLT twice a week only 2 months after T1. In the first 2 months, the therapy was delivered online due to COVID-19. Based on the behavioral and electrophysiological results, therapy goals were determined by the first author (JS) in collaboration with the two SLPs (SS and LD’H) who delivered the SLT to the patient. The therapy focused on training phonological input and output, word fluency, syntactic production, word comprehension, and sentence comprehension. In the phonological training, she had to discriminate phonemes and combine syllables and letters into words. To practice word fluency, she had to generate words within semantic categories (varying in frequency) and generate words that started with a given sound. Phonological and semantic cues were given in these exercises to stimulate errorless learning. In the syntactic training, she practiced completing sentences, composing sentences with keywords, judging the grammaticality of sentences, and describing pictures. To train word comprehension and production, she had to describe words and pictures, name words based on a description, and produce synonyms and antonyms. Finally, the patient asked the SLPs to practice reading the newspaper. Reading aloud and text comprehension was therefore trained by the newspaper “Wablieft” in which short and clear sentences are used. Six months after the SLT was initiated (T2), almost the same results as at T1 were found. In addition, the subtests spoken and written sentence comprehension of the CAT-NL were impaired at T2. The DIAS also confirmed the presence of AOS at T2 since three characteristics of AOS were present. Differences in the presence, mean amplitudes, and onset latencies of the ERP components were found by the five paradigms at T1 and T2 in comparison to the HC group. Furthermore, differences were found between the results of each paradigm at T1 and T2. The electrophysiological results are presented in Table 3.

#### Patient With Logopenic Variant (No Speech-Language Therapy)

This patient consulted the neurologist because of difficulties with speaking and short-term memory. The neuropsychological examination showed a normal neurocognitive profile except for deficits in visual alternating attention and left-right orientation. MRI was unremarkable and FDG-PET scan suggested hypometabolism in the right precuneus of unknown significance. CSF biomarker profile was not compatible with Alzheimer’s disease. One year after the initial consultation at the Department of Neurology (T1), the language and speech abilities were evaluated by the CAT-NL, the DIAS, and the electrophysiological test battery. His spontaneous speech was characterized by word-finding difficulties, circumlocutions, phonological and semantic paraphasias, and unfinished sentences. Scores outside the normative range were only found in the repetition subcategory of the CAT-NL (mainly complex words and sentences subtests). Consequently, the clinical diagnosis of the LV was made (Gorno-Tempini et al., 2011). At that time, the patient was not interested to start with SLT. Six months later (T2), the same results were found on the behavioral assessments. However, the patient reported that he had more word-finding difficulties, difficulties in conversations (by phone), and difficulties discussing with people. The results of the MMN paradigm were unreliable due to a low number of included trials. Differences in the presence, mean amplitudes, and onset latencies of the ERP components were found by the four paradigms at both T1 and T2 in comparison to the HC group. Furthermore, differences were found between the results at T1 and the results at T2. The electrophysiological results can be found in Table 4.

**TABLE 4:** The electrophysiological results of each ERP component in the patients with LV and SV who did not receive SLT at the initial assessment and the follow-up assessment.

	LV		SV	
	T1 vs. HCs	T2 vs. T1	T1 vs. HCs	T2 vs. T1
MMN (inattentive oddball paradigm)	–	–	Present	Present
P300 (attentive oddball paradigm)	Present F, C, L, M, R: delayed F, R: prolonged	F: no delay or prolongation P: delayed C, L: prolonged	Present	F, L, M, R: delayed
N400 (categorical priming paradigm)	Absent	Absent	Absent	Absent
N400 (semantic anomaly paradigm)	Present F, P, L, M: prolonged R: delayed	C, P, L, M: delayed Not prolonged	–	–
LPC (semantic anomaly paradigm)	Present Prolonged F, C, P, L, M, R: delayed	R: no delay	–	–
P600 (grammaticality judgment paradigm)	Present F, C, L, M: delayed Prolonged	R: delayed F, C, P, M, R: accelerated decay, MA ↓	–	–

*The differences in mean amplitude, onset latency, and topographic distribution between the initial assessment and the healthy control group are presented in the column “T1 vs. HCs.” In the column “T2 vs. T1,” the differences in mean amplitude, onset latency, and topographic distribution between the initial and the follow-up assessments are presented. LV, logopenic variant; SV, semantic variant; T1, initial assessment; T2, follow-up assessment; HC, healthy control; MMN, mismatch negativity; LPC, late positivity complex; F, frontal electrode sites; C, central electrode sites; P, parietal electrode sites; L, left electrode sites; M, midline electrode sites; R, right electrode sites; MA ↓, reduced mean amplitude; MA ↑, increased mean amplitude; –, unreliable data.*

#### Patient With Semantic Variant (No Speech-Language Therapy)

This patient consulted the neurologist because of word-finding difficulties and memory problems. The imaging results (MRI and FDG-PET) indicated the possible diagnosis of the SV based on predominant atrophy and hypometabolism on the anterior temporal lobes bilaterally. However, the evaluation of the language abilities by an SLP showed that not all criteria of the SV were met at the time of the initial neurological consultation. Twenty months after the initial consultation (T1), the patient was included in this study. The CAT-NL and the DIAS did not reveal any deficits except for a score outside the normative range for the writing to dictation subtest due to an error in an irregular word which suggests the possible presence of surface dysgraphia. Furthermore, the patient complained about deficits in word comprehension and word-finding difficulties. In a spontaneous conversation between the patient and the first author (JS), impaired word comprehension and word-finding difficulties were also present. His spontaneous speech was also characterized by circumlocutions, semantic paraphasias, neologisms, and logorrhea. Based on these results, he met the two core diagnostic criteria and possibly three of the four supportive criteria of the SV (Gorno-Tempini et al., 2011). The patient was not interested to start SLT. Six months later (T2), no deficits were found in the behavioral assessments and the patient complained of the same language difficulties. The results of the semantic anomaly paradigm and the P600 paradigm were unreliable due to a low number of included trials. In this patient, it is important to mention that at both assessments and in all paradigms much alpha activity was present which complicated the visual inspection. The electrophysiological results are presented in Table 4.

### Electrophysiological Changes: Speech-Language Therapy vs. No Speech-Language Therapy

In general, electrophysiological changes were present after approximately 6 months in multiple ERP components in each included patient. More specifically, the mean amplitudes, the onset latencies, and/or the duration of the ERP components differed between the initial assessment and the follow-up assessment. The changes in these variables are presented in Table 5 for both the patients that received SLT and the patients that did not receive SLT.

**TABLE 5:** Summary of the electrophysiological changes that occurred after approximately 6 months in patients with primary progressive aphasia that did and did not receive speech-language therapy.

Electrophysiological changes		% of ERP components in patients with SLT	% of ERP components in patients with no SLT
Mean amplitude	Increased	25	0
	Decreased	25	25
Onset latency	Increased delay	25	50
	Decreased delay	33.3	12.5
Duration	Increased prolongation	8.3	12.5
	Decreased prolongation	16.7	12.5
	Accelerated decay	0	12.5
	No more accelerated decay	8.3	0
No electrophysiological changes		16.7	37.5

*SLT, speech-language therapy.*

## Discussion

This preliminary study aimed to explore therapy-induced electrophysiological changes in persons with PPA by the language-related MMN, P300, N400, and P600 components. Four patients with PPA of which two received SLT and two did not receive SLT were included in this study. Since the effectivity of SLT is most frequently measured by standardized language assessments in clinical practice, we will first discuss the results on the CAT-NL of the initial and follow-up assessments in the four patients. The results remained stable in the two patients that did not receive SLT. In the patients that received SLT, the score of one additional subtest and two additional subtests were outside the normative range at the follow-up assessment in respectively the patients with NFV and PPA-NOS. Importantly, almost no scores of the subtests of the CAT-NL were outside the normative range in the four patients and no language deficits were found in the subtests that investigated language comprehension except for the follow-up assessment of the patient with PPA-NOS. These results do not provide much information about the linguistic deterioration or therapy-induced changes of the specific language processes, or how to adapt and determine therapy goals for SLT.

While the results on the CAT-NL remained relatively stable between the initial and the follow-up assessments, changes in the mean amplitudes, onset latencies, and duration of the ERP components were found in the four patients. The most important changes that were found in the two patients that did not receive SLT were an increased delay in 50% and a decreased mean amplitude in 25% of the measured ERP components. These results might indicate that the evaluated language comprehension processes deteriorated between the initial and follow-up assessments. The electrophysiological changes found in the patients that received SLT were more variable. Our most important findings were a decreased delay in 33.3% and an increased mean amplitude in 25% of the measured ERP components which might suggest improvements and/or the presence of compensation mechanisms in language comprehension processes. Cocquyt et al. (2020) also found that an increased amplitude might reflect gains of SLT in persons with aphasia after stroke. In contrast, the mean amplitude decreased and the delay increased in 25% of the measured ERP components which might indicate that some language comprehension processes progressively deteriorated despite the SLT. These electrophysiological results provide information about (therapy-induced) changes in specific language comprehension processes which behavioral language assessments cannot provide.

Focusing on the results of the MMN and P300 components, no clear difference between the electrophysiological changes in the patients who received SLT and the patients who did not receive SLT was found. Interestingly, the timing of the processes associated with phoneme categorization was more delayed at the follow-up assessment than at the initial assessment regardless of the therapy condition. In the patient with the NFV (who received SLT), the MMN at the follow-up assessment was less delayed and the mean amplitude was increased in comparison to the initial assessment. This decreased delay and increased mean amplitude might suggest improvements and/or the occurrence of compensation mechanisms in the processes associated with phoneme discrimination. The same results were found for the N400 effect elicited by the categorical priming paradigm in this patient which might also indicate improvements and/or the presence of compensation mechanisms in the processes involved in this paradigm. Since this N400 effect elicited by the categorical priming paradigm was absent in the two patients that did not receive SLT, no conclusions can be drawn about the differences in electrophysiological changes in the patients that did and did not receive SLT. Concerning the results of the N400 effect elicited by the semantic anomaly paradigm and the P600, the electrophysiological changes between the initial and follow-up assessments were very variable. An interesting finding was that the P600 component was absent at the initial assessment but present at the follow-up assessment in the patient with the NFV, although only at the parietal electrode sites. This result might also suggest improvements and/or the presence of compensation mechanisms in the syntactic reanalysis and repair mechanisms. Although no clear patterns in electrophysiological changes between patients who received SLT and patients who did not receive SLT were found by our preliminary study, it seems like the SLT induced improvements or compensation mechanisms in some specific language comprehension processes in the patient with the NFV. The presence of compensation mechanisms due to SLT was also found in the case studies of Dressel et al. (2010), Marcotte and Ansaldo (2010), and Beeson et al. (2011).

The results of this study are still preliminary because only four patients were included. In the interpretation of these results, it is important to take into account that the patients were all various clinical phenotypes of PPA who had various language and speech difficulties, were in another disease stage, and possibly had a varying speed of linguistic decline. In future research, it is important to compare larger groups of patients with the same variant because the underlying pathology and the clinical characteristics can differ between variants. Furthermore, the ERP technique is characterized by the presence of individual differences between averaged ERP waveforms across subjects and the presence of alpha activity may severely diminish the ability to identify an ERP component in some individuals such as the patient with the SV (Luck, 2014). However, the main advantage of the ERP technique is its temporal resolution to provide continuous records of neural processing in the order of milliseconds. In contrast to behavioral assessments, ERPs give us objective information about the neural processes from the period before the presentation of the stimulus, the period between the stimulus and the response, and also the period after the response. These linguistic ERPs may provide information about the nature and timing of the processing deficiencies in patients with PPA which the behavioral assessments cannot provide (Luck, 2014). ERPs are also considered to have a moderate to high test-retest reliability which is an important characteristic for a measurement technique to evaluate the effectiveness of SLT (Lew et al., 2007; Cassidy et al., 2012; Kiang et al., 2013; Besche-Richard et al., 2014). Future studies should include larger patient groups of the three clinical variants because the therapy-induced electrophysiological changes might differ depending on the clinical variant and the underlying pathology. It is also important for further research to correlate the electrophysiological changes with changes in language functions, functional communication, and quality of life of the patient to identify which type of SLT is the most beneficial for each patient. Finally, the types of SLT and the usability of the ERP technique to determine and monitor therapy goals and plans should be investigated.

## Data Availability Statement

The raw data supporting the conclusions of this article will be made available by the authors, without undue reservation.

## Ethics Statement

The studies involving human participants were reviewed and approved by the Ethics Committee of Ghent University Hospital. The patients/participants provided their written informed consent to participate in this study. Written informed consent was obtained from the individual(s) for the publication of any potentially identifiable images or data included in this article.

## Author Contributions

JS and MD contributed to the design of the experiments. TV and AS recruited the patients with PPA. SS and LD’H provided therapy for the patient with PPA-NOS. JS contributed to the data collection, data analysis, interpretation of the results, and writing of the manuscript under the supervision of MD and PM. MD, PM, TV, MM, AS, WD, LD’H, and SS provided critical feedback and helped shape the manuscript. All authors approved the final version for submission.

## Conflict of Interest

The authors declare that the research was conducted in the absence of any commercial or financial relationships that could be construed as a potential conflict of interest.

## Publisher’s Note

All claims expressed in this article are solely those of the authors and do not necessarily represent those of their affiliated organizations, or those of the publisher, the editors and the reviewers. Any product that may be evaluated in this article, or claim that may be made by its manufacturer, is not guaranteed or endorsed by the publisher.

## Footnotes

1 Stalpaert, J., Miatton, M., Sieben, A., Van Langenhove, T., van Mierlo, P., and De Letter, M. (under review). Syntactic event-related potentials in the diagnostic process of the nonfluent and logopenic variants of primary progressive aphasia: a case series. *Int. J. Lang. Commun. Disord.*

## References

[B1] Aaltonen O. Niemi P. Nyrke T. Tuhkanen M. (1987). Event-related brain potentials and the perception of a phonetic continuum. *Biol. Psychol.* 24 197–207. 10.1016/0301-0511(87)90002-0 3663795

[B2] Aerts A. van Mierlo P. Hartsuiker R. J. Hallez H. Santens P. De Letter M. (2013). Neurophysiological investigation of phonological input: aging effects and development of normative data. *Brain Lang.* 125 253–263. 10.1016/j.bandl.2013.02.010 23542728

[B3] Alain C. Tremblay K. (2007). The role of event-related brain potentials in assessing central auditory processing. *J. Am. Acad. Audiol.* 18 573–589. 10.3766/jaaa.18.7.5 18236645

[B4] Beeson P. M. King R. M. Bonakdarpour B. Henry M. L. Cho H. Rapcsak S. Z. (2011). Positive effects of language treatment for the logopenic variant of primary progressive aphasia. *J. Mol. Neurosci.* 45 724–736. 10.1007/s12031-011-9579-2 21710364 PMC3208072

[B5] Besche-Richard C. Iakimova G. Hardy-Baylé M. Passerieux C. (2014). Behavioral and brain measures (N400) of semantic priming in patients with schizophrenia: test-retest effect in a longitudinal study. *Psychiatry Clin. Neurosci.* 68 365–373. 10.1111/pcn.12137 24405516

[B6] Brysbaert M. Stevens M. De Deyne S. Voorspoels W. Storms G. (2014). Norms of age of acquisition and concreteness for 30,000 Dutch words. *Acta Psychol.* 150 80–84. 10.1016/j.actpsy.2014.04.010 24831463

[B7] Cadório I. Lousada M. Martins P. Figueiredo D. (2017). Generalization and maintenance of treatment gains in primary progressive aphasia (PPA): a systematic review. *Int. J. Lang. Commun. Disord.* 52 543–560. 10.1111/1460-6984.12310 28120406

[B8] Carthery-Goulart M. T. da Silveira A. D. C. Machado T. H. Mansur L. L. Parente M. Senaha M. L. H. (2013). Nonpharmacological interventions for cognitive impairments following primary progressive aphasia: a systematic review of the literature. *Dement. Neuropsychol.* 7 122–131. 10.1590/s1980-57642013dn70100018 29213828 PMC5619554

[B9] Cassidy S. M. Robertson I. H. O’Connell R. G. (2012). Retest reliability of event-related potentials: evidence from a variety of paradigms. *Psychophysiology* 49 659–664. 10.1111/j.1469-8986.2011.01349.x 22335452

[B10] Cocquyt E. M. Santens P. van Mierlo P. Duyck W. Szmalec A. De Letter M. (2021). Age- and gender-related differences in verbal semantic processing: the development of normative electrophysiological data in the Flemish population. *Lang. Cogn. Neurosci.* 37 241–267. 10.1080/23273798.2021.1957137

[B11] Cocquyt E. M. Vandewiele M. Bonnarens C. Santens P. De Letter M. (2020). The sensitivity of event-related potentials/fields to logopedic interventions in patients with stroke-related aphasia. *Acta Neurol. Belgica* 120 805–817. 10.1007/s13760-020-01378-3 32474880

[B12] Crawford J. R. Garthwaite P. H. Azzalini A. Howell D. C. Laws K. R. (2006). Testing for a deficit in single-case studies: effects of departures from normality. *Neuropsychologia* 44 666–677. 10.1016/j.neuropsychologia.2005.06.001 16046228

[B13] Dressel K. Huber W. Frings L. Kümmerer D. Saur D. Mader I. (2010). Model-oriented naming therapy in semantic dementia: a single-case fMRI study. *Aphasiology* 24 1537–1558. 10.1080/02687038.2010.500567

[B14] Feiken J. Jonkers R. (2012). *Diagnostisch Instrument Voor Apraxie Van De Spraak.* Houten: Bohn Stafleu van Loghum.

[B15] Friederici A. D. (2002). Towards a neural basis of auditory sentence processing. *Trends Cogn. Sci.* 6 78–84. 10.1016/s1364-6613(00)01839-8 15866191

[B16] Friederici A. D. (2004). Event-related brain potential studies in language. *Curr. Neurol. Neurosci. Rep.* 4 466–470. 10.1007/s11910-004-0070-0 15509448

[B17] Gorno-Tempini J. Hillis A. E. Weintraub S. Kertesz A. Mendez M. Cappa S. F. (2011). Classification of primary progressive aphasia and its variants. *Neurology* 76 1006–1014. 10.1212/WNL.0b013e31821103e6 21325651 PMC3059138

[B18] Henry M. L. Hubbard H. I. Grasso S. M. Dial H. R. Beeson P. M. Miller B. L. (2019). Treatment for word retrieval in semantic and logopenic variants of primary progressive aphasia: immediate and long-term outcomes. *J. Speech Lang. Hear. Sci.* 62 2723–2749. 10.1044/2018_JSLHR-L-18-0144 PMC6802912 31390290

[B19] Kaan E. Dallas A. C. Barkley C. M. (2007). Processing bare quantifiers in discourse. *Brain Res.* 1146 199–209. 10.1016/j.brainres.2006.09.060 17070788 PMC1900382

[B20] Kallionpää R. Pesonen H. Scheinin A. Sandman N. Laitio R. Scheinin H. (2019). Single-subject analysis of N400 event-related potential component with five different methods. *Int. J. Psychophysiol.* 144 14–24. 10.1016/j.ijpsycho.2019.06.012 31228496

[B21] Keuleers E. Brysbaert M. New B. (2010). SUBTLEX-NL: a new measure for dutch word frequency based on film subtitles. *Behav. Res. Methods* 42 643–650. 10.3758/BRM.42.3.643 20805586

[B22] Kiang M. Patriciu I. Roy C. Christensen B. K. Zipursky R. B. (2013). Test-retest reliability and stability of N400 effects in a word-pair semantic priming paradigm. *Clin. Neurophysiol.* 124 667–674. 10.1016/j.clinph.2012.09.029 23122708

[B23] Kielar A. Meltzer-Asscher A. Thompson C. K. (2012). Electrophysiological responses to argument structure violations in healthy adults and individuals with agrammatic aphasia. *Neuropsychologia* 50 3320–3337. 10.1016/j.neuropsychologia.2012.09.013 23022079 PMC3518698

[B24] Kim A. Osterhout L. (2005). The independence of combinatory semantic processing: evidence from event-related potentials. *J. Mem. Lang.* 52 205–225. 10.1016/j.jml.2004.10.002

[B25] Kok A. (2001). On the utility of P3 amplitude as a measure of processing capacity. *Psychophysiology* 38 557–577. 10.1017/s0048577201990559 11352145

[B26] Kutas M. Federmeier K. D. (2011). Thirty years and counting: finding meaning in the N400 component of the event-related brain potential (ERP). *Ann. Rev. Psychol.* 62 621–647. 10.1146/annurev.psych.093008.131123 20809790 PMC4052444

[B27] Lau E. F. Phillips C. Poeppel D. (2008). A cortical network for semantics: (de)constructing the N400. *Nature Rev. Neurosci.* 9 920–933. 10.1038/nrn2532 19020511

[B28] Lew H. L. Gray M. Poole J. H. (2007). Temporal stability of auditory event-related potentials in healthy individuals and patients with traumatic brain injury. *J. Clin. Neurophysiol.* 24 392–397. 10.1097/WNP.0b013e31814a56e3 17912063

[B29] Luck S. J. (2014). *An Introduction To The Event-Related Potential Technique.* Cambridge, MA: MIT Press.

[B30] Luck S. J. Kappenman E. S. (2012). *The Oxford Handbook of Event-Related Potential Components.* Oxford: Oxford University Press.

[B31] Marcotte K. Ansaldo A. I. (2010). The neural correlates of semantic feature analysis in chronic aphasia: discordant patterns according to the etiology. *Semin. Speech Lang.* 31 52–63. 10.1055/s-0029-1244953 20221954

[B32] Marian V. Bartolotti J. Chabal S. Shook A. (2012). CLEARPOND: cross-linguistic easy-access resource for phonological and orthographic neighborhood densities. *PLoS One* 7:e43230. 10.1371/journal.pone.0043230 22916227 PMC3423352

[B33] Mesulam E. J. Wieneke C. Hurley R. S. Geula C. Bigio E. H. Thompson C. K. (2014). Primary progressive aphasia and the evolving neurology of the language network. *Nat. Rev. Neurol.* 10:554. 10.1038/nrneurol.2014.159 25179257 PMC4201050

[B34] Moors A. De Houwer J. Hermans D. Wanmaker S. Van Schie K. Van Harmelen A. L. (2013). Norms of valence, arousal, dominance, and age of acquisition for 4,300 Dutch words. *Behav. Res. Methods* 45 169–177. 10.3758/s13428-012-0243-8 22956359

[B35] Näätänen R. Kujala T. Escera C. Baldeweg T. Kreegipuu K. Carlson S. (2012). The mismatch negativity (MMN) - a unique window to disturbed central auditory processing in ageing and different clinical conditions. *Clin. Neurophysiol.* 123 424–458. 10.1016/j.clinph.2011.09.020 22169062

[B36] Näätänen R. Lehtokoski A. Lennes M. Cheour M. Huotilainen M. Iivonen A. (1997). Language-specific phoneme representations revealed by electric and magnetic brain responses. *Nature* 385 432–434. 10.1038/385432a0 9009189

[B37] Nasreddine Z. S. Phillips N. A. Bédirian V. Charbonneau S. Whitehead V. Collin I. (2005). The montreal cognitive assessment. MoCA: a brief screening tool for mild cognitive impairment. *J. Am. Geriatr. Soc.* 53 695–699. 10.1111/j.1532-5415.2005.53221.x 15817019

[B38] Rogalski E. J. Khayum B. (2018). A life participation approach to primary progressive aphasia intervention. *Semin. Speech Lang.* 39 284–296. 10.1055/s-0038-1660786 29933494 PMC6350508

[B39] Rogalski E. J. Saxon M. McKenna H. Wieneke C. Rademaker A. Corden M. E. (2016). Communication bridge: a pilot feasibility study of Internet-based speech-language therapy for individuals with progressive aphasia. *Alzheimers Dementia* 2 213–221. 10.1016/j.trci.2016.08.005 28503656 PMC5423699

[B40] Stalpaert J. Cocquyt E.-M. Criel Y. Segers L. Miatton M. Van Langenhove T. (2020). Language and speech markers of primary progressive aphasia: a systematic review. *Am. J. Speech Lang. Pathol.* 29 2206–2225. 10.1044/2020_AJSLP-20-00008 32810414

[B41] Stalpaert J. Cocquyt E.-M. Miatton M. Sieben A. Van Langenhove T. van Mierlo P. (2021a). A case series of verbal semantic processing in primary progressive aphasia: evidence from the N400 effect. *Int. J. Lang. Commun. Disord.* 56 1165–1189. 10.1111/1460-6984.12658 34357662

[B42] Stalpaert J. Miatton M. Sieben A. Van Langenhove T. van Mierlo P. De Letter M. (2021b). The electrophysiological correlates of phoneme perception in primary progressive aphasia: a preliminary case series. *Front. Hum. Neurosci.* 15:618549. 10.3389/fnhum.2021.618549 34149376 PMC8206281

[B43] Swinburn K. Porter G. Howard D. (2014). *Comprehensive Aphasia Test Nederlandstalige Bewerking (CAT-NL) (E. Visch-Brink, D. Vandenborre, H. J. de Smet*, & *P. Mariën, Trans.). Pearson Assessment and Information B.V.* København: Dansk Psykologisk Forlag.

[B44] Van Strien J. W. (1992). Classificatie van links- en rechtshandige proefpersonen. *Ned. Tijdschr. Voor Psychol. Haar Grensgebieden* 47 88–92.

[B45] Van Vliet M. Manyakov N. V. Storms G. Fias W. Wiersema J. R. Van Hulle M. M. (2014). Response-related potentials during semantic priming: the effect of a speeded button response task on ERPs. *PLoS One* 9:e87650. 10.1371/journal.pone.0087650 24516556 PMC3916390

[B46] Volkmer A. Rogalski E. Henry M. Taylor-Rubin C. Ruggero L. Khayum R. (2020a). Speech and language therapy approaches to managing primary progressive aphasia. *Pract. Neurol.* 20 154–161. 10.1136/practneurol-2018-001921 31358572 PMC6986989

[B47] Volkmer A. Spector A. Meitanis V. Warren J. D. Beeke S. (2020b). Effects of funcstional communication interventions for people with primary progressive aphasia and their caregivers: a systematic review. *Aging Mental Health* 24 1381–1393. 10.1080/13607863.2019.1617246 31134821

