# Supplementary material for: Therapy-Induced Electrophysiological Changes in Primary Progressive Aphasia: A Preliminary Study
Source: Front Hum Neurosci. 2022 Mar 31;16:766866. doi: 10.3389/fnhum.2022.766866 (PMC9008202; doi:10.3389/fnhum.2022.766866)
Supplement: Supplementary file 1 [file Data_Sheet_1.docx]

Supplementary Material

# Supplementary Figures and Tables of the patients with PPA

**MMN**

**Table 1.** Raw values of the mean amplitudes of the difference waves (deviant – standard condition) for each patient over the time windows 150ms – 250ms, 250ms – 350ms, and 350ms – 450ms at the frontal, central, parietal, left, midline, and right electrode sites.

| Variant |  | NFV  (SLT) | | PPA-NOS (SLT) | | SV  (no SLT) | | |
| --- | --- | --- | --- | --- | --- | --- | --- | --- |
|  |  | T1 | T2 | T1 | T2 | T1 | T2 |  |
| 150-250 | F | **-0.62** | -1.74 | **-0.03** | -0.71 | -1.61 | -1.17 |  |
|  | C | -0.65 | -1.34 | **-0.25** | **-0.31** | -1.51 | -1.17 |  |
|  | P | -0.59 | -0.88 | -0.79 | **0.69** | -1.2 | -1.00 |  |
|  | L | -0.68 | -1.18 | -0.52 | **-0.30** | -1.55 | -0.88 |  |
|  | M | -0.59 | -1.32 | **-0.31** | **-0.17** | -1.45 | -1.18 |  |
|  | R | -0.59 | -1.46 | **-0.24** | **0.14** | -1.32 | -1.29 |  |
| 250-350 | F | -2.35 | -2.46 | -0.48 | -0.02 | -0.73 | -0.69 |  |
|  | C | -1.7 | -2.21 | -0.53 | **0.32** | -0.78 | -0.86 |  |
|  | P | -0.88 | -0.79 | -0.67 | **1.05** | -0.88 | -1.14 |  |
|  | L | -1.67 | -1.59 | -0.65 | **0.30** | -0.71 | -0.50 |  |
|  | M | -1.59 | -1.90 | -0.6 | **0.49** | -0.81 | -0.92 |  |
|  | R | -1.68 | -1.98 | -0.44 | **0.55** | -0.87 | -1.27 |  |
| 350-450 | F | **0.91** | 0.23 | -0.28 | -0.69 | -0.41 | -0.62 |  |
|  | C | 0.14 | -0.64 | -0.49 | -0.34 | -0.54 | -0.71 |  |
|  | P | -0.28 | -0.48 | -0.66 | 0.33 | -0.88 | -0.81 |  |
|  | L | 0.27 | -0.21 | -0.29 | -0.39 | -0.75 | -0.68 |  |
|  | M | 0.42 | -0.12 | -0.51 | -0.26 | -0.55 | -0.74 |  |
|  | R | 0.09 | -0.57 | -0.64 | -0.04 | -0.53 | -0.72 |  |

Abbreviations: F = frontal; C = central; P = parietal; L = left; M = midline; R = right; NFV = nonfluent variant; SV = semantic variant; PPA-NOS = primary progressive aphasia not otherwise specified; SLT = speech-language therapy; in bold = reduced mean amplitude (Z ≥ 1.28); underlined = increased mean amplitude (Z ≤ -1.28).

**Table 2.** Raw values of the onset latencies of the difference waves (deviant – standard condition) for each patient at the frontal, central, parietal, left, midline, and right electrode sites.

| Variant | NFV  (SLT) | | PPA-NOS  (SLT) | | SV  (no SLT) | |
| --- | --- | --- | --- | --- | --- | --- |
|  | T1 | T2 | T1 | T2 | T1 | T2 |
| F | **239.33** | 223.33 | **265.33** | 197.33 | 193.33 | 219.33 |
| C | 226 | 230.67 | 222.67 | 195.33 | 198.67 | 222.67 |
| P | 230 | 215.33 | 212 | 324.67 | 215.33 | 230.00 |
| L | **237.33** | 215.33 | 216.67 | 196.00 | 195.33 | 221.33 |
| M | 228.67 | 224.00 | **242** | 261.33 | 202 | 224.00 |
| R | 229.33 | 230.00 | **241.33** | 260.00 | 210 | 226.67 |

Abbreviations: F = frontal; C = central; P = parietal; L = left; M = midline; R = right; NFV = nonfluent variant; SV = semantic variant; PPA-NOS = primary progressive aphasia not otherwise specified; SLT = speech-language therapy; in bold = delayed (Z ≥ 1.28).

**Table 3.** Z-scores of the mean amplitudes of the difference waves (deviant – standard condition) for each patient over the time windows 150ms – 250ms, 250ms – 350ms, and 350ms – 450ms at the frontal, central, parietal, left, midline, and right electrode sites.

| Variant |  | NFV  (SLT) | | PPA-NOS  (SLT) | | SV  (no SLT) | |  |
| --- | --- | --- | --- | --- | --- | --- | --- | --- |
|  |  | T1 | T2 | T1 | T2 | T1 | T2 | |
| 150-250 | F | **1.29** | -0.18 | **2.06** | 1.17 | -0.02 | 0.57 | |
|  | C | 0.94 | 0.00 | **1.48** | **1.40** | -0.23 | 0.23 | |
|  | P | 0.31 | -0.17 | -0.01 | **2.45** | -0.69 | -0.37 | |
|  | L | 0.9 | 0.02 | 1.17 | **1.57** | -0.62 | 0.55 | |
|  | M | 1.03 | 0.01 | **1.42** | **1.61** | -0.16 | 0.21 | |
|  | R | 0.88 | -0.37 | **1.39** | **1.94** | -0.18 | -0.13 | |
| 250-350 | F | -1.63 | -1.77 | 0.68 | 1.25 | 0.37 | 0.42 | |
|  | C | -1.07 | -1.70 | 0.4 | **1.46** | 0.09 | -0.01 | |
|  | P | -0.59 | -0.45 | -0.28 | **2.33** | -0.6 | -0.99 | |
|  | L | -1.42 | -1.29 | 0.19 | **1.70** | 0.09 | 0.43 | |
|  | M | -0.98 | -1.36 | 0.27 | **1.66** | 0.01 | -0.12 | |
|  | R | -1.22 | -1.63 | 0.48 | **1.84** | -0.11 | -0.66 | |
| 350-450 | F | **1.5** | 0.67 | 0.05 | -0.46 | -0.1 | -0.37 | |
|  | C | 0.54 | -0.42 | -0.23 | -0.05 | -0.3 | -0.50 | |
|  | P | 0.14 | -0.16 | -0.42 | 1.01 | -0.73 | -0.63 | |
|  | L | 0.81 | 0.11 | -0.02 | -0.17 | -0.71 | -0.59 | |
|  | M | 0.97 | 0.29 | -0.19 | 0.11 | -0.24 | -0.49 | |
|  | R | 0.59 | -0.27 | -0.37 | 0.42 | -0.22 | -0.49 | |

Abbreviations: F = frontal; C = central; P = parietal; L = left; M = midline; R = right; NFV = nonfluent variant; SV = semantic variant; PPA-NOS = primary progressive aphasia not otherwise specified; SLT = speech-language therapy; in bold = reduced mean amplitude (Z ≥ 1.28); underlined = increased mean amplitude (Z ≤ -1.28).

**Table 4.** Z-scores of the onset latencies of the difference waves (deviant – standard condition) for each patient at the frontal, central, parietal, left, midline, and right electrode sites.

| Variant | NFV  (SLT) | | PPA-NOS  (SLT) | | SV  (no SLT) | |  |
| --- | --- | --- | --- | --- | --- | --- | --- |
|  | T1 | T2 | T1 | T2 | T1 | T2 | |
| F | **1.86** | 0.89 | **3.44** | -0.69 | -0.93 | 0.65 | |
| C | 0.91 | 1.20 | 0.7 | -1.00 | -0.79 | 0.70 | |
| P | 0.65 | -0.05 | -0.21 | **5.16** | -0.05 | 0.65 | |
| L | **1.46** | 0.15 | 0.23 | -1.00 | -1.04 | 0.51 | |
| M | 0.95 | 0.69 | **1.7** | **2.79** | -0.55 | 0.69 | |
| R | 1.09 | 1.13 | **1.84** | **3.01** | -0.13 | 0.92 | |

Abbreviations: F = frontal; C = central; P = parietal; L = left; M = midline; R = right; NFV = nonfluent variant; SV = semantic variant; PPA-NOS = primary progressive aphasia not otherwise specified; SLT = speech-language therapy; in bold = delayed (Z ≥ 1.28).

**P300**

**Table 5.** Raw values of the mean amplitudes of the difference waves (deviant – standard condition) for each patient over the time windows 350ms – 550ms, 550ms – 750ms, and 750ms – 950ms at the frontal, central, parietal, left, midline, and right electrode sites.

| Variant |  | NFV  (SLT) | | PPA-NOS  (SLT) | | | LV  (no SLT) | | | SV  (no SLT) | |
| --- | --- | --- | --- | --- | --- | --- | --- | --- | --- | --- | --- |
|  |  | T1 | T2 | T1 | T2 | T1 | | T2 | T1 | | T2 |
| 350-550 | F | 2.21 | -2.16 | -0.74 | -0.29 | -0.56 | | -1.34 | 0.43 | | **-3.49** |
|  | C | 2.36 | -0.89 | -0.68 | -1.01 | -0.85 | | -1.29 | 0.18 | | -0.97 |
|  | P | 3.92 | 0.63 | -1.42 | **-1.61** | 0.44 | | -0.51 | 1.06 | | 2.25 |
|  | L | 3.28 | -0.41 | -1.08 | -0.90 | -0.33 | | -1.30 | -0.34 | | -1.71 |
|  | M | 3.41 | -0.64 | -1.08 | -1.25 | -0.6 | | -1.20 | 0.48 | | -1.24 |
|  | R | 1.8 | -1.37 | -0.68 | -0.75 | -0.04 | | -0.63 | 1.53 | | 0.73 |
| 550-750 | F | -2.15 | **-6.00** | -1.47 | 0.44 | 0.7 | | 1.21 | 0.35 | | -1.03 |
|  | C | -1.94 | **-3.42** | -1.48 | -0.15 | 1.46 | | 1.79 | 0.68 | | 0.88 |
|  | P | 1.43 | -0.58 | **-2.23** | -0.49 | 2.07 | | 1.88 | 2.88 | | 3.18 |
|  | L | -0.55 | **-3.56** | -1.77 | -0.55 | 1.17 | | 1.49 | 1.42 | | 0.83 |
|  | M | -1.17 | **-3.47** | -2.09 | -0.15 | 1.32 | | 1.80 | 0.99 | | 0.48 |
|  | R | -0.94 | **-2.97** | -1.32 | 0.51 | 1.74 | | 1.59 | 1.5 | | 1.71 |
| 750-950 | F | -1.29 | -3.65 | -1.84 | -0.33 | 1.74 | | 0.89 | -0.53 | | -1.07 |
|  | C | -1.11 | -1.05 | -1.48 | -0.48 | 0.9 | | 1.96 | 0.07 | | -1.43 |
|  | P | -0.41 | -1.13 | -1.35 | 0.10 | 1.25 | | 2.23 | 1.92 | | -0.12 |
|  | L | -1.2 | -2.97 | -1.69 | -0.47 | 0.94 | | 1.16 | 1.01 | | -0.81 |
|  | M | -1.75 | -1.67 | -1.62 | -0.05 | 0.7 | | 1.90 | -0.25 | | -1.85 |
|  | R | 0.13 | -1.18 | -1.37 | -0.19 | 2.25 | | 2.02 | 0.7 | | 0.03 |

Abbreviations: F = frontal; C = central; P = parietal; L = left; M = midline; R = right; NFV = nonfluent variant; LV = logopenic variant; SV = semantic variant; PPA-NOS = primary progressive aphasia not otherwise specified; SLT = speech-language therapy; in bold = reduced mean amplitude (Z ≤ -1.28); underlined = increased mean amplitude (Z ≥ 1.28).

**Table 6.** Raw values of the onset latencies of the difference waves (deviant – standard condition) for each patient at the frontal, central, parietal, left, midline, and right electrode sites.

| Variant | NFV  (SLT) | | PPA-NOS  (SLT) | | LV  (no SLT) | | SV  (no SLT) | |
| --- | --- | --- | --- | --- | --- | --- | --- | --- |
|  | T1 | T2 | T1 | T2 | T1 | T2 | T1 | T2 |
| F | 426.67 | 384.67 | 422 | 478.67 | **682.67** | 637.33 | 384.67 | **792.67** |
| C | 442 | **565.33** | 414.67 | 526.67 | **622** | **662.67** | 510.67 | 549.33 |
| P | 464 | 460.67 | 432.67 | **707.33** | 573.33 | **668.00** | 553.33 | 483.33 |
| L | 427.33 | 449.33 | 402 | 523.33 | **621.33** | **638.67** | 546 | **629.33** |
| M | 432 | 430.00 | 412.67 | **624.67** | **628** | **659.33** | 436.67 | **640.67** |
| R | 473.33 | 531.33 | 454.67 | **564.67** | **628.67** | **670.00** | 466 | **555.33** |

Abbreviations: F = frontal; C = central; P = parietal; L = left; M = midline; R = right; NFV = nonfluent variant; LV = logopenic variant; SV = semantic variant; PPA-NOS = primary progressive aphasia not otherwise specified; SLT = speech-language therapy; in bold = delayed (Z ≥ 1.28).

**Table 7.** Z-scores of the mean amplitudes of the difference waves (deviant – standard condition) for each patient over the time windows 350ms – 550ms, 550ms – 750ms, and 750ms – 950ms at the frontal, central, parietal, left, midline, and right electrode sites.

| Variant |  | NFV  (SLT) | | PPA-NOS  (SLT) | | LV  (no SLT) | | SV  (no SLT) | |
| --- | --- | --- | --- | --- | --- | --- | --- | --- | --- |
|  |  | T1 | T2 | T1 | T2 | T1 | T2 | T1 | T2 |
| 350-550 | F | 0.31 | -1.15 | -0.67 | -0.52 | -0.61 | -0.88 | -0.28 | **-1.60** |
|  | C | 0.3 | -0.64 | -0.58 | -0.67 | -0.63 | -0.75 | -0.33 | -0.66 |
|  | P | 0.18 | -0.68 | -1.21 | **-1.26** | -0.73 | -0.98 | -0.57 | -0.26 |
|  | L | 0.59 | -0.67 | -0.9 | -0.83 | -0.64 | -0.97 | -0.64 | -1.11 |
|  | M | 0.38 | -0.80 | -0.92 | -0.97 | -0.78 | -0.96 | -0.47 | -0.97 |
|  | R | -0.12 | -1.16 | -0.93 | -0.96 | -0.73 | -0.92 | -0.21 | -0.47 |
| 550-750 | F | -0.44 | **-2.07** | -0.15 | 0.66 | 0.77 | 0.98 | 0.62 | 0.03 |
|  | C | -0.85 | **-1.39** | -0.68 | -0.19 | 0.4 | 0.52 | 0.11 | 0.19 |
|  | P | -0.44 | -1.13 | **-1.7** | -1.10 | -0.22 | -0.29 | 0.05 | 0.16 |
|  | L | -0.44 | **-1.83** | -1.01 | -0.44 | 0.36 | 0.51 | 0.47 | 0.20 |
|  | M | -0.73 | **-1.65** | -1.1 | -0.32 | 0.27 | 0.46 | 0.14 | -0.07 |
|  | R | -0.88 | **-1.84** | -1.06 | -0.20 | 0.39 | 0.32 | 0.27 | 0.37 |
| 750-950 | F | 0 | -1.07 | -0.25 | 0.43 | 1.37 | 0.99 | 0.34 | 0.10 |
|  | C | -0.14 | -0.11 | -0.32 | 0.17 | 0.84 | 1.36 | 0.44 | -0.30 |
|  | P | -0.22 | -0.54 | -0.64 | 0.01 | 0.53 | 0.97 | 0.83 | -0.09 |
|  | L | -0.05 | -1.06 | -0.33 | 0.37 | 1.18 | 1.31 | 1.22 | 0.18 |
|  | M | -0.44 | -0.40 | -0.38 | 0.34 | 0.68 | 1.23 | 0.25 | -0.49 |
|  | R | 0.16 | -0.65 | -0.76 | -0.03 | 1.47 | 1.32 | 0.51 | 0.10 |

Abbreviations: F = frontal; C = central; P = parietal; L = left; M = midline; R = right; NFV = nonfluent variant; LV = logopenic variant; SV = semantic variant; PPA-NOS = primary progressive aphasia not otherwise specified; SLT = speech-language therapy; in bold = reduced mean amplitude (Z ≤ -1.28); underlined = increased mean amplitude (Z ≥ 1.28).

**Table 8.** Z-scores of the onset latencies of the difference waves (deviant – standard condition) for each patient at the frontal, central, parietal, left, midline, and right electrode sites.

| Variant | NFV  (SLT) | | PPA-NOS  (SLT) | | LV  (no SLT) | | SV  (no SLT) | |
| --- | --- | --- | --- | --- | --- | --- | --- | --- |
|  | T1 | T2 | T1 | T2 | T1 | T2 | T1 | T2 |
| F | -0.53 | -0.88 | -0.57 | -0.11 | **1.58** | 1.20 | -0.88 | **2.48** |
| C | -0.43 | **1.33** | -0.83 | 0.78 | **2.15** | **2.73** | 0.55 | 1.10 |
| P | -0.38 | -0.42 | -0.76 | **2.64** | 0.98 | **2.15** | 0.73 | -0.14 |
| L | -0.7 | -0.45 | -1 | 0.42 | **1.56** | **1.77** | 0.68 | **1.66** |
| M | -0.65 | -0.67 | -0.87 | **1.52** | **1.56** | **1.91** | -0.6 | **1.70** |
| R | -0.12 | 0.79 | -0.42 | **1.32** | **2.33** | **2.99** | -0.24 | **1.17** |

Abbreviations: F = frontal; C = central; P = parietal; L = left; M = midline; R = right; NFV = nonfluent variant; LV = logopenic variant; SV = semantic variant; PPA-NOS = primary progressive aphasia not otherwise specified; SLT = speech-language therapy; in bold = delayed (Z ≥ 1.28).

**Categorical priming paradigm**

**Table 9.** Raw values of the mean amplitudes of the difference waves (different category – same category condition) for each patient over the time windows 300ms – 500ms, 500ms – 700ms, and 700ms – 900ms at the frontal, central, parietal, left, midline, and right electrode sites.

| Variant |  | NFV  (SLT) | | PPA-NOS  (SLT) | | LV  (no SLT) | | SV  (no SLT) | |
| --- | --- | --- | --- | --- | --- | --- | --- | --- | --- |
|  |  | T1 | T2 | T1 | T2 | T1 | T2 | T1 | T2 |
| 300-500 | F | 0.28 | -2.35 | -0.93 | 0.52 | -0.55 | -0.76 | -0.55 | -0.76 |
|  | C | -0.13 | -2.51 | -0.42 | 0.43 | -0.61 | -0.76 | -0.61 | -0.76 |
|  | P | -0.67 | -2.12 | 0.15 | 0.40 | -1.12 | -1.12 | -1.12 | -1.12 |
|  | L | 0.33 | -1.90 | -0.16 | 0.30 | -0.76 | -0.63 | -0.76 | -0.63 |
|  | M | -0.36 | -3.06 | -0.28 | 0.46 | -0.85 | -0.69 | -0.85 | -0.69 |
|  | R | -0.48 | -2.03 | -0.76 | 0.59 | -0.67 | -1.31 | -0.67 | -1.31 |
| 500-700 | F | -1.78 | -2.46 | -1.26 | -1.05 | -0.07 | 0.02 | -0.07 | 0.02 |
|  | C | -1.74 | -2.95 | -0.76 | -0.96 | -0.10 | -0.45 | -0.10 | -0.45 |
|  | P | -2.06 | -3.80 | -1.09 | -0.95 | -0.71 | -0.91 | -0.71 | -0.91 |
|  | L | -1.41 | -2.66 | -0.4 | -0.73 | **0.38** | 0.06 | **0.38** | 0.06 |
|  | M | -1.95 | -3.99 | -1.12 | -1.14 | -0.30 | -0.20 | -0.30 | -0.20 |
|  | R | -2.23 | -2.56 | -1.58 | -1.08 | -0.95 | -1.20 | -0.95 | -1.20 |
| 700-900 | F | -0.34 | -2.10 | -0.77 | -0.91 | **1.23** | **1.23** | **1.23** | **1.23** |
|  | C | -1.09 | -1.27 | -0.67 | -1.45 | **1.56** | **0.80** | **1.56** | **0.80** |
|  | P | -1.51 | -1.25 | -1.5 | -1.87 | **0.93** | -0.76 | **0.93** | -0.76 |
|  | L | -0.57 | -1.23 | -0.33 | -1.29 | **1.97** | **1.08** | **1.97** | **1.08** |
|  | M | -0.95 | -2.34 | -1.03 | -1.77 | **1.25** | **0.94** | **1.25** | **0.94** |
|  | R | -1.42 | -1.05 | -1.57 | -1.17 | 0.49 | -0.75 | 0.49 | -0.75 |

Abbreviations: F = frontal; C = central; P = parietal; L = left; M = midline; R = right; NFV = nonfluent variant; LV = logopenic variant; SV = semantic variant; PPA-NOS = primary progressive aphasia not otherwise specified; SLT = speech-language therapy; in bold = reduced mean amplitude (Z ≥ 1.28); underlined = increased mean amplitude (Z ≤ -1.28).

**Table 10.** Raw values of the onset latencies of the difference waves (different category – same category condition) for each patient at the frontal, central, parietal, left, midline, and right electrode sites.

| Variant | NFV  (SLT) | | PPA-NOS  (SLT) | | LV  (no SLT) | | SV  (no SLT) | |
| --- | --- | --- | --- | --- | --- | --- | --- | --- |
|  | T1 | T2 | T1 | T2 | T1 | T2 | T1 | T2 |
| F | 530 | 450.67 | 455.33 | **586.67** | **589.33** | 416.00 | 424.67 | 368.00 |
| C | 532.67 | 444.00 | 454.67 | 594.67 | 391.33 | 506.67 | 427.33 | 398.00 |
| P | 526.67 | 477.33 | 584 | **612.00** | 522 | 583.33 | 412 | 444.67 |
| L | 545.33 | 459.33 | 458 | **600.00** | 516.67 | 529.33 | 365.33 | 392.67 |
| M | 520 | 462.67 | 528.67 | **604.67** | 494 | 460.00 | 441.33 | 391.33 |
| R | 524 | 450.00 | 507.33 | 588.67 | 492 | 516.67 | 457.33 | 426.67 |

Abbreviations: F = frontal; C = central; P = parietal; L = left; M = midline; R = right; NFV = nonfluent variant; LV = logopenic variant; SV = semantic variant; PPA-NOS = primary progressive aphasia not otherwise specified; SLT = speech-language therapy; in bold = delayed (Z ≥ 1.28).

**Table 11.** Z-scores of the mean amplitudes of the difference waves (different category – same category condition) for each patient over the time windows 300ms – 500ms, 500ms – 700ms, and 700ms – 900ms at the frontal, central, parietal, left, midline, and right electrode sites.

| Variant |  | NFV  (SLT) | | PPA-NOS  (SLT) | | LV  (no SLT) | | SV  (no SLT) | |
| --- | --- | --- | --- | --- | --- | --- | --- | --- | --- |
|  |  | T1 | T2 | T1 | T2 | T1 | T2 | T1 | T2 |
| 300-500 | F | 0.82 | -1.40 | -0.2 | 1.02 | 1.24 | 0.59 | 0.12 | -0.06 |
|  | C | 0.39 | -1.99 | 0.1 | 0.95 | 0.93 | 0.59 | -0.09 | -0.24 |
|  | P | -0.17 | -1.71 | 0.7 | 0.97 | 0.32 | **1.39** | -0.65 | -0.65 |
|  | L | 0.97 | -1.34 | 0.47 | 0.95 | 0.98 | 0.85 | -0.15 | -0.02 |
|  | M | 0.27 | -2.08 | 0.34 | 0.98 | 0.86 | 0.87 | -0.16 | -0.02 |
|  | R | -0.05 | -1.69 | -0.34 | 1.10 | 0.83 | 0.84 | -0.25 | -0.93 |
| 500-700 | F | -0.74 | -1.24 | -0.35 | -0.20 | **1.74** | 0.89 | 0.52 | 0.58 |
|  | C | -0.18 | -1.17 | 0.64 | 0.47 | **1.46** | 1.04 | 1.18 | 0.89 |
|  | P | -0.11 | -1.47 | 0.65 | 0.76 | 0.94 | **1.44** | 0.95 | 0.79 |
|  | L | -0.06 | -1.13 | 0.8 | 0.52 | **1.48** | 0.99 | **1.47** | 1.19 |
|  | M | -0.28 | -1.80 | 0.34 | 0.32 | **1.43** | **1.28** | 0.96 | 1.03 |
|  | R | -0.75 | -1.02 | -0.21 | 0.20 | **1.39** | 1.20 | 0.3 | 0.09 |
| 700-900 | F | 0.03 | -1.45 | -0.33 | -0.45 | 1.08 | 0.40 | **1.35** | **1.35** |
|  | C | -0.21 | -0.37 | 0.14 | -0.51 | 0.88 | 0.62 | **2.01** | **1.38** |
|  | P | -0.22 | -0.03 | -0.21 | -0.49 | 0.46 | 1.02 | **1.59** | 0.33 |
|  | L | 0.11 | -0.48 | 0.33 | -0.53 | 0.57 | 0.45 | **2.38** | **1.58** |
|  | M | -0.07 | -1.15 | -0.14 | -0.71 | 0.81 | 0.87 | **1.64** | **1.40** |
|  | R | -0.47 | -0.15 | -0.6 | -0.26 | 1.08 | 0.83 | 1.17 | 0.10 |

Abbreviations: F = frontal; C = central; P = parietal; L = left; M = midline; R = right; NFV = nonfluent variant; LV = logopenic variant; SV = semantic variant; PPA-NOS = primary progressive aphasia not otherwise specified; SLT = speech-language therapy; in bold = reduced mean amplitude (Z ≥ 1.28); underlined = increased mean amplitude (Z ≤ -1.28).

**Table 12.** Z-scores of the onset latencies of the difference waves (different category – same category condition) for each patient at the frontal, central, parietal, left, midline, and right electrode sites.

| Variant | NFV  (SLT) | | PPA-NOS  (SLT) | | LV  (no SLT) | | SV  (no SLT) | |
| --- | --- | --- | --- | --- | --- | --- | --- | --- |
|  | T1 | T2 | T1 | T2 | T1 | T2 | T1 | T2 |
| F | 0.92 | -0.17 | -0.1 | **1.70** | **1.73** | -0.64 | -0.52 | -1.30 |
| C | 0.28 | -0.99 | -0.84 | 1.17 | -1.74 | -0.09 | -1.23 | -1.65 |
| P | -0.03 | -0.88 | 0.97 | **1.45** | -0.11 | 0.95 | -2.02 | -1.45 |
| L | 0.92 | -0.41 | -0.43 | **1.77** | 0.48 | 0.68 | -1.87 | -1.45 |
| M | 0.35 | -0.58 | 0.49 | **1.72** | -0.07 | -0.62 | -0.92 | -1.73 |
| R | 0.05 | -0.82 | -0.15 | 0.80 | -0.32 | -0.04 | -0.73 | -1.09 |

Abbreviations: F = frontal; C = central; P = parietal; L = left; M = midline; R = right; NFV = nonfluent variant; LV = logopenic variant; SV = semantic variant; PPA-NOS = primary progressive aphasia not otherwise specified; SLT = speech-language therapy; in bold = delayed (Z ≥ 1.28).

**Semantic anomaly paradigm**

**Table 13.** Raw values of the mean amplitudes of the difference waves (incorrect – correct sentences) for each patient over the time windows 300ms – 500ms, 500ms – 700ms, 700ms – 900ms, and 900ms – 1100ms at the frontal, central, parietal, left, midline, and right electrode sites.

| Variant |  | NFV  (SLT) | | PPA-NOS  (SLT) | | LV  (no SLT) | |
| --- | --- | --- | --- | --- | --- | --- | --- |
|  |  | T1 | T2 | T1 | T2 | T1 | T2 |
| 300-500 | F | **1.28** | **1.93** | -0.3 | -0.81 | -0.87 | 0.36 |
|  | C | -1 | **0.02** | -0.41 | -1.27 | -1.73 | **0.07** |
|  | P | -2.95 | -1.10 | -1.27 | -0.43 | -2 | **0.41** |
|  | L | -0.3 | **0.29** | -0.81 | -0.97 | -1.55 | -0.30 |
|  | M | -0.89 | **0.36** | -0.9 | -0.76 | -2.03 | **0.49** |
|  | R | -1.47 | **0.19** | -0.27 | -0.76 | -1.02 | **0.65** |
| 500-700 | F | 1.59 | 3.00 | -0.22 | -1.14 | -2.32 | -0.74 |
|  | C | -0.35 | **2.74** | -0.75 | -1.40 | -1.25 | -1.14 |
|  | P | -2.01 | **2.65** | -1.16 | -0.21 | -1.33 | -0.74 |
|  | L | 0.21 | 2.92 | -0.58 | -0.99 | -1.75 | -1.02 |
|  | M | 0.08 | **3.56** | -0.88 | -0.76 | -1.83 | -0.64 |
|  | R | -1.06 | 1.91 | -0.66 | -1.01 | -1.33 | -0.96 |
| 700-900 | F | 1.9 | **4.64** | 0.29 | -0.83 | -1.36 | 0.27 |
|  | C | 1.58 | **4.86** | 0.15 | -1.10 | 1.1 | 0.57 |
|  | P | -0.36 | **3.84** | 0.37 | -0.36 | 0.64 | 0.54 |
|  | L | 0.91 | **3.96** | 0.12 | -0.82 | -0.63 | -0.16 |
|  | M | 1.59 | **5.40** | 0.46 | -0.52 | -0.02 | 0.63 |
|  | R | 0.62 | **3.98** | 0.24 | -0.96 | 1.03 | 0.91 |
| 900-1100 | F | 2.06 | **3.64** | 1.07 | 0.25 | 0.14 | 0.56 |
|  | C | 1.95 | **3.56** | 1.32 | 0.40 | 0.85 | 0.98 |
|  | P | -0.28 | 2.42 | 1.18 | 0.68 | -0.58 | 0.76 |
|  | L | 0.71 | **2.70** | 0.62 | 0.66 | -0.76 | 0.52 |
|  | M | 2.04 | **3.67** | 1.52 | 0.89 | -0.14 | 0.88 |
|  | R | 0.97 | **3.26** | 1.43 | -0.22 | 1.3 | 0.91 |

Abbreviations: F = frontal; C = central; P = parietal; L = left; M = midline; R = right; NFV = nonfluent variant; LV = logopenic variant; PPA-NOS = primary progressive aphasia not otherwise specified; SLT = speech-language therapy; in bold = reduced mean amplitude N400 or increased mean amplitude LPC (Z ≥ 1.28); underlined = increased mean amplitude N400 or reduced mean amplitude LPC (Z ≤ -1.28).

**Table 14.** Raw values of the onset latencies of the difference waves (incorrect – correct sentences) for each patient at the frontal, central, parietal, left, midline, and right electrode sites.

| Variant |  | NFV  (SLT) | | PPA-NOS  (SLT) | | LV  (no SLT) | |
| --- | --- | --- | --- | --- | --- | --- | --- |
|  |  | T1 | T2 | T1 | T2 | T1 | T2 |
| N400 | F | 355.33 | 459.33 | 447.33 | 442.67 | 504 | 524.00 |
|  | C | 432 | 400.67 | **486** | 441.33 | 418 | **514.00** |
|  | P | 408 | 384.67 | 426.67 | 417.33 | 400.67 | **526.00** |
|  | L | 390.67 | 422.67 | 440.67 | 442.67 | 439.33 | **496.00** |
|  | M | 377.33 | 415.33 | 438.67 | 416.67 | 430.67 | **528.00** |
|  | R | 427.33 | 406.67 | 480.67 | 442.00 | **452.67** | **540.00** |
| LPC | F | 696.67 | 682.67 | **830.67** | **885.33** | **953.33** | **826.67** |
|  | C | **807.33** | 698.67 | **884.67** | **923.33** | **833.33** | **834.00** |
|  | P | **834.67** | 674.00 | **887.33** | 756.67 | **821.33** | **834.67** |
|  | L | 720 | 666.00 | **842** | **910.67** | **876.67** | **892.00** |
|  | M | **785.33** | 681.33 | **884** | **838.00** | **889.33** | **807.33** |
|  | R | **833.33** | 708.00 | **876.67** | **816.67** | **842** | 796.00 |

Abbreviations: F = frontal; C = central; P = parietal; L = left; M = midline; R = right; NFV = nonfluent variant; LV = logopenic variant; PPA-NOS = primary progressive aphasia not otherwise specified; SLT = speech-language therapy; in bold = delayed (Z ≥ 1.28).

**Table 15.** Z-scores of the mean amplitudes of the difference waves (incorrect – correct sentences) for each patient over the time windows 350ms – 550ms, 550ms – 750ms, and 750ms – 950ms at the frontal, central, parietal, left, midline, and right electrode sites.

| Variant |  | NFV  (SLT) | | PPA-NOS  (SLT) | | LV  (no SLT) | |
| --- | --- | --- | --- | --- | --- | --- | --- |
|  |  | T1 | T2 | T1 | T2 | T1 | T2 |
| 300-500 | F | **1.49** | **2.01** | 0.25 | -0.15 | -0.2 | 0.77 |
|  | C | 0.4 | **1.31** | 0.93 | 0.16 | -0.26 | **1.36** |
|  | P | -1.6 | 0.35 | 0.17 | 1.06 | -0.6 | **1.93** |
|  | L | 0.66 | **1.31** | 0.1 | -0.08 | -0.72 | 0.66 |
|  | M | 0.37 | **1.60** | 0.37 | 0.50 | -0.75 | **1.73** |
|  | R | -0.15 | **1.64** | 1.14 | 0.61 | 0.34 | **2.12** |
| 500-700 | F | 0.48 | 1.24 | -0.49 | -0.99 | -1.62 | -0.77 |
|  | C | -0.49 | **1.28** | -0.72 | -1.10 | -1.01 | -0.95 |
|  | P | -1.84 | **1.30** | -1.27 | -0.63 | -1.39 | -0.99 |
|  | L | -0.45 | 1.27 | -0.96 | -1.21 | -1.7 | -1.23 |
|  | M | -0.48 | **1.62** | -1.06 | -0.98 | -1.62 | -0.91 |
|  | R | -0.82 | 1.21 | -0.55 | -0.79 | -1.01 | -0.75 |
| 700-900 | F | 0.55 | **2.30** | -0.48 | -1.19 | -1.53 | -0.49 |
|  | C | 0.11 | **1.91** | -0.67 | -1.36 | -0.15 | -0.44 |
|  | P | -1.1 | **1.30** | -0.68 | -1.10 | -0.53 | -0.59 |
|  | L | -0.36 | **1.57** | -0.86 | -1.44 | -1.33 | -1.02 |
|  | M | 0.03 | **2.14** | -0.59 | -1.13 | -0.86 | -0.50 |
|  | R | -0.24 | **1.94** | -0.49 | -1.27 | 0.03 | -0.05 |
| 900-1100 | F | 1.12 | **2.18** | 0.46 | -0.09 | -0.17 | 0.12 |
|  | C | 0.75 | **1.55** | 0.44 | -0.02 | 0.2 | 0.27 |
|  | P | -0.47 | 1.14 | 0.4 | 0.10 | -0.65 | 0.15 |
|  | L | 0.11 | **1.33** | 0.06 | 0.08 | -0.79 | -0.01 |
|  | M | 0.86 | **1.77** | 0.57 | 0.22 | -0.35 | 0.22 |
|  | R | 0.49 | **1.88** | 0.78 | -0.35 | 0.7 | 0.38 |

Abbreviations: F = frontal; C = central; P = parietal; L = left; M = midline; R = right; NFV = nonfluent variant; LV = logopenic variant; PPA-NOS = primary progressive aphasia not otherwise specified; SLT = speech-language therapy; in bold = reduced mean amplitude N400 or increased mean amplitude LPC (Z ≥ 1.28); underlined = increased mean amplitude N400 or reduced mean amplitude LPC (Z ≤ -1.28).

**Table 16.** Z-scores of the onset latencies of the difference waves (incorrect – correct sentences) for each patient at the frontal, central, parietal, left, midline, and right electrode sites.

| Variant |  | NFV  (SLT) | | PPA-NOS  (SLT) | | LV  (no SLT) | |
| --- | --- | --- | --- | --- | --- | --- | --- |
|  |  | T1 | T2 | T1 | T2 | T1 | T2 |
| N400 | F | -0.66 | 0.32 | 0.21 | 0.16 | 0.74 | 0.93 |
|  | C | 0.59 | -0.06 | **1.71** | 0.78 | 0.3 | **2.28** |
|  | P | 0.18 | -0.30 | 0.57 | 0.38 | 0.03 | **2.61** |
|  | L | -0.21 | 0.46 | 0.84 | 0.88 | 0.81 | **2.00** |
|  | M | -0.45 | 0.16 | 0.53 | 0.18 | 0.41 | **1.97** |
|  | R | -1.15 | -0.26 | 0.45 | 0.36 | **1.43** | **2.06** |
| LPC | F | -0.02 | -0.17 | **1.42** | **2.01** | **2.74** | **1.38** |
|  | C | **1.48** | 0.16 | **2.43** | **2.90** | **1.8** | **1.81** |
|  | P | **1.83** | -0.19 | **2.49** | 0.85 | **1.66** | **1.83** |
|  | L | 0.44 | -0.25 | **1.99** | **2.86** | **2.43** | **2.62** |
|  | M | **1.4** | 0.00 | **2.73** | **2.11** | **2.8** | **1.70** |
|  | R | **1.51** | 0.01 | **2.03** | **1.31** | **1.61** | 1.06 |

Abbreviations: F = frontal; C = central; P = parietal; L = left; M = midline; R = right; NFV = nonfluent variant; LV = logopenic variant; PPA-NOS = primary progressive aphasia not otherwise specified; SLT = speech-language therapy; in bold = delayed (Z ≥ 1.28).

**P600**

**Table 17*.*** Raw values of the mean amplitudes of the difference waves (incorrect – correct condition) for each patient over the time windows 500ms – 750ms, 750ms – 1000ms, and 1000ms – 1250ms at the frontal, central, parietal, left, midline, and right electrode sites.

| Variant |  | NFV  (SLT) | | PPA-NOS  (SLT) | | LV  (no SLT) | |
| --- | --- | --- | --- | --- | --- | --- | --- |
|  |  | T1 | T2 | T1 | T2 | T1 | T2 |
| 500-750 | F | **-1.95** | **-3.04** | 3.92 | 0.28 | **-2.49** | -1.73 |
|  | C | -0.89 | **-1.89** | 4.26 | 0.93 | 0.28 | -0.32 |
|  | P | 0.06 | 0.49 | 1.69 | -0.65 | 2.44 | 0.36 |
|  | L | -1.21 | **-1.72** | 3.43 | -0.24 | -0.99 | -1.44 |
|  | M | -0.76 | **-1.15** | 4.09 | 0.38 | 0.51 | -0.41 |
|  | R | -0.81 | **-1.56** | 2.35 | 0.42 | 0.71 | 0.16 |
| 750-1000 | F | **-2.46** | **-3.94** | 3.66 | 2.07 | 0.68 | -0.56 |
|  | C | -0.37 | **-0.74** | 4.76 | 2.10 | 3.27 | **1.03** |
|  | P | 0.31 | 3.65 | 2.89 | 0.25 | 3.71 | **1.00** |
|  | L | **-1.67** | -0.52 | 3.78 | 0.98 | 1.37 | -1.03 |
|  | M | -0.3 | 0.12 | 4.86 | 1.80 | 2.7 | **0.59** |
|  | R | **-0.54** | **-0.62** | 2.67 | 1.65 | 3.59 | **1.91** |
| 1000-1250 | F | **-2.21** | **-5.77** | 1.92 | 1.97 | 2.98 | **-0.04** |
|  | C | -0.11 | **-3.50** | 3.1 | 2.14 | 4.54 | **1.97** |
|  | P | -0.85 | -0.51 | 2.65 | 0.18 | 3.47 | 1.51 |
|  | L | -1.14 | **-3.03** | 3.18 | 1.03 | 3.42 | 0.32 |
|  | M | -0.52 | **-3.22** | 3.37 | 1.67 | 3.95 | **0.96** |
|  | R | **-1.5** | **-3.53** | 1.13 | 1.59 | 3.62 | **2.16** |

Abbreviations: F = frontal; C = central; P = parietal; L = left; M = midline; R = right; NFV = nonfluent variant; LV = logopenic variant; PPA-NOS = primary progressive aphasia not otherwise specified; SLT = speech-language therapy; in bold = reduced mean amplitude (Z ≤ -1.28).

**Table 18*.*** Raw values of the onset latencies of the difference waves (incorrect – correct condition) for each patient at the frontal, central, parietal, left, midline, and right electrode sites.

| Variant | NFV  (SLT) | | PPA-NOS  (SLT) | | LV  (no SLT) | |
| --- | --- | --- | --- | --- | --- | --- |
|  | T1 | T2 | T1 | T2 | T1 | T2 |
| F | 634.67 | 644.00 | 740.67 | 812.00 | **996.67** | **954.00** |
| C | 756 | 763.33 | 678.67 | 774.00 | **855.33** | **900.00** |
| P | 663.33 | 762.00 | 718.67 | **872.67** | 746.67 | 788.67 |
| L | 655.33 | 704.00 | 680 | **850.67** | **921.33** | **986.67** |
| M | 757.33 | 692.67 | 736.67 | **820.67** | **862.67** | **818.67** |
| R | 641.33 | 772.67 | 721.33 | 787.33 | 814.67 | **837.33** |

Abbreviations: F = frontal; C = central; P = parietal; L = left; M = midline; R = right; NFV = nonfluent variant; LV = logopenic variant; PPA-NOS = primary progressive aphasia not otherwise specified; SLT = speech-language therapy; in bold = delayed (Z ≥ 1.28).

**Table 19*.*** Z-scores of the mean amplitudes of the difference waves (incorrect – correct condition) for each patient over the time windows 500ms – 750ms, 750ms – 1000ms, and 1000ms – 1250ms at the frontal, central, parietal, left, midline, and right electrode sites.

| Variant |  | NFV  (SLT) | | PPA-NOS  (SLT) | | LV  (no SLT) | |  |
| --- | --- | --- | --- | --- | --- | --- | --- | --- |
|  |  | T1 | T2 | T1 | T2 | T1 | T2 | |
| 500-750 | F | **-1.39** | **-1.77** | 0.67 | -0.60 | **-1.58** | -0.63 | |
|  | C | -1.14 | **-1.48** | 0.67 | -0.49 | -0.72 | -1.24 | |
|  | P | -0.75 | -0.61 | -0.24 | -0.96 | -0.01 | -0.88 | |
|  | L | -1.16 | **-1.36** | 0.68 | -0.78 | -1.07 | -0.83 | |
|  | M | -1.17 | **-1.29** | 0.41 | -0.80 | -0.75 | -1.06 | |
|  | R | -1.16 | **-1.45** | 0.03 | -0.70 | -0.59 | -1.08 | |
| 750-1000 | F | **-1.58** | **-2.12** | 0.62 | 0.05 | -0.46 | -0.99 | |
|  | C | -1.2 | **-1.32** | 0.53 | -0.37 | 0.03 | **-1.60** | |
|  | P | -1.06 | 0.02 | -0.23 | -1.08 | 0.04 | **-1.43** | |
|  | L | **-1.49** | -1.03 | 0.65 | -0.44 | -0.29 | -0.98 | |
|  | M | -1.23 | -1.10 | 0.41 | -0.56 | -0.28 | **-1.53** | |
|  | R | **-1.39** | **-1.42** | -0.14 | -0.54 | 0.21 | **-1.79** | |
| 1000-1250 | F | **-1.48** | **-3.03** | 0.31 | 0.34 | 0.77 | **-1.30** | |
|  | C | -0.72 | **-1.94** | 0.43 | 0.08 | 0.94 | **-1.44** | |
|  | P | -0.98 | -0.86 | 0.27 | -0.61 | 0.56 | -0.94 | |
|  | L | -0.94 | **-1.72** | 0.85 | -0.04 | 0.95 | -0.63 | |
|  | M | -0.86 | **-1.78** | 0.47 | -0.11 | 0.67 | **-1.33** | |
|  | R | **-1.46** | **-2.33** | -0.33 | -0.13 | 0.74 | **-1.81** | |

Abbreviations: F = frontal; C = central; P = parietal; L = left; M = midline; R = right; NFV = nonfluent variant; LV = logopenic variant; PPA-NOS = primary progressive aphasia not otherwise specified; SLT = speech-language therapy; in bold = reduced mean amplitude (Z ≤ -1.28).

**Table 20*.*** Z-scores of the onset latencies of the difference waves (incorrect – correct condition) for each patient at the frontal, central, parietal, left, midline, and right electrode sites.

| Variant | NFV  (SLT) | | PPA-NOS  (SLT) | | LV  (no SLT) | |  |
| --- | --- | --- | --- | --- | --- | --- | --- |
|  | T1 | T2 | T1 | T2 | T1 | T2 | |
| F | -0.72 | -0.62 | 0.34 | 1.04 | **2.88** | **2.45** | |
| C | 0.53 | 0.63 | -0.56 | 0.78 | **1.93** | **2.55** | |
| P | -0.9 | 0.64 | -0.03 | **2.37** | 0.4 | 1.06 | |
| L | -0.79 | -0.12 | -0.45 | **1.90** | **2.87** | **3.77** | |
| M | 0.81 | -0.20 | 0.48 | **1.80** | **2.45** | **1.77** | |
| R | -1.19 | 0.62 | -0.09 | 0.82 | 1.2 | **1.51** | |

Abbreviations: F = frontal; C = central; P = parietal; L = left; M = midline; R = right; NFV = nonfluent variant; LV = logopenic variant; PPA-NOS = primary progressive aphasia not otherwise specified; SLT = speech-language therapy; in bold = delayed (Z ≥ 1.28).

**Behavioral language and speech assessments**

**Table 21.** Raw scores (corresponding C-scores) of all subtests of the CAT-NL for each patient.

| Patient |  | NFV | | LV | | SV | | PPA-NOS | |
| --- | --- | --- | --- | --- | --- | --- | --- | --- | --- |
| Speech-language therapy | | yes | | no | | no | | yes | |
|  | Total | T1 | T2 | T1 | T2 | T1 | T2 | T1 | T2 |
| Cognitive screening |  | 37 (9) | 36 (9) | 39 (10) | 39 (10) | 38 (10) | 34 (8) | 33 (7) | 33 (7) |
| Line bisection |  | 0 (6) | 0 (6) | 0 (6) | 0 (6) | 0 (6) | 0 (6) | 0 (6) | 0 (6) |
| Semantic memory | 10 | 10 (6) | 10 (6) | 10 (6) | 10 (6) | 10 (6) | 9 (4) | 10 (6) | 10 (6) |
| Fluency |  | 22 (8) | 17 (7) | 39 (10) | 46 (10) | 32 (9) | 33 (9) | **11 (5)** | **13 (6)** |
| Recognition memory | 10 | 10 (5) | 10 (5) | 10 (5) | 10 (5) | 9 (4) | 9 (4) | 9 (4) | 10 (5) |
| Ideational praxis | 12 | 11 (6) | 11 (6) | 11 (6) | 11 (6) | 12 (7) | 11 (6) | 11 (6) | 11 (6) |
| Arithmetic | 6 | 6 (6) | 6 (6) | 6 (6) | 6 (6) | 6 (6) | 5 (5) | 6 (6) | 6 (6) |
| Comprehension |  | 18 (10) | 17 (9) | 17 (9) | 17 (9) | 17 (9) | 18 (10) | 14 (7) | 11 (5) |
| Spoken total | 66 | 66 (9) | 62 (7) | 61 (7) | 62 (7) | 65 (9) | 63 (8) | 64 (8) | 57 (5) |
| Spoken words | 30 | 30 (7) | 26 (5) | 28 (6) | 29 (7) | 29 (7) | 28 (6) | 30 (7) | 30 (7) |
| Spoken sentences | 32 | 32 (8) | 32 (8) | 29 (6) | 30 (7) | 32 (8) | 32 (8) | 30 (7) | **23 (4)** |
| Spoken paragraphs | 4 | 4 (6) | 4 (6) | 4 (6) | 3 (5) | 4 (6) | 3 (5) | 4 (6) | 4 (6) |
| Written total | 62 | 61 (9) | 62 (10) | 62 (10) | 62 (10) | 60 (8) | 62 (10) | 55 (6) | 54 (6) |
| Written words | 30 | 30 (6) | 30 (6) | 30 (6) | 30 (6) | 30 (6) | 30 (6) | 30 (6) | 30 (6) |
| Written sentences | 32 | 31 (9) | 32 (9) | 32 (9) | 32 (9) | 30 (8) | 32 (9) | 25 (6) | **24 (5)** |
| Production |  | 30 (8) | 28 (7) | 30 (8) | 31 (9) | 31 (9) | 33 (10) | 29 (8) | 27 (7) |
| Repetition total | 61 | 57 (7) | 51 (5) | **48 (5)** | **46 (5)** | 58 (7) | 59 (8) | 56 (7) | **48 (5)** |
| Words | 32 | 32 (6) | 30 (5) | 28 (5) | 28 (5) | 32 (6) | 32 (6) | 32 (6) | 30 (5) |
| Complex words | 6 | 6 (6) | 4 (5) | 4 (5) | 4 (5) | 6 (6) | 6 (6) | 6 (6) | 4 (5) |
| Nonwords | 10 | 9 (6) | 8 (6) | 6 (5) | 4 (4) | 10 (7) | 10 (7) | 10 (7) | 6 (5) |
| Numbers | 7 | 4 (5) | 4 (5) | 5 (6) | 5 (6) | 5 (6) | 6 (8) | 4 (5) | 4 (5) |
| Sentences | 6 | 6 (7) | 5 (6) | 5 (6) | 5 (6) | 5 (6) | 5 (6) | **4 (5)** | **4 (5)** |
| Naming total | 58 | 77 (8) | 69 (7) | 91 (10) | 104 (10) | 83 (9) | 79 (9) | 62 (7) | 65 (7) |
| Objects | 48 | 45 (9) | 42 (8) | 42 (8) | 48 (10) | 41 (8) | 36 (6) | 42 (8) | 42 (8) |
| Actions | 10 | 10 (7) | 10 (7) | 10 (7) | 10 (7) | 10 (7) | 10 (7) | 10 (7) | 10 (7) |
| Reading aloud total | 70 | 68 (7) | 63 (6) | 70 (8) | 70 (8) | 70 (8) | 70 (8) | 68 (7) | 66 (7) |
| Words | 48 | 46 (6) | **41 (5)** | 48 (7) | 48 (7) | 48 (7) | 48 (7) | 48 (7) | 47 (7) |
| Complex words | 6 | 6 (6) | 6 (6) | 6 (6) | 6 (6) | 6 (6) | 6 (6) | 6 (6) | 5 (6) |
| Function words | 6 | 6 (5) | 6 (5) | 6 (5) | 6 (5) | 6 (5) | 6 (5) | 6 (5) | 6 (5) |
| Nonwords | 10 | 10 (7) | 10 (7) | 10 (7) | 10 (7) | 10 (7) | 10 (7) | 8 (6) | 8 (6) |
| Writing total | 83 | 81 (8) | 81 (8) | 80 (7) | 82 (8) | 77 (7) | 81 (8) | 81 (8) | 81 (8) |
| Copying | 31 | 31 (6) | 31 (6) | 31 (6) | 31 (6) | 31 (6) | 31 (6) | 31 (6) | 31 (6) |
| Naming objects | 23 | 22 (7) | 22 (7) | 22 (7) | 23 (7) | 22 (7) | 22 (7) | 22 (7) | 22 (7) |
| To dictation | 28 | 28 (7) | 28 (7) | 27 (6) | 28 (7) | **24 (5)** | 28 (7) | 28 (7) | 28 (7) |

Abbreviations: NFV = nonfluent variant; LV = logopenic variant; SV = semantic variant; PPA-NOS = not otherwise specified; scores in bold = impaired; T1 = initial assessment; T2 = follow-up assessment.

**Table 22**. Raw scores of all subtests of the DIAS for each patient.

| Patient |  | NFV | | LV | | SV | | PPA-NOS | |
| --- | --- | --- | --- | --- | --- | --- | --- | --- | --- |
| Speech-language therapy |  | yes | | no | | no | | yes | |
|  | Total | T1 | T2 | T1 | T2 | T1 | T2 | T1 | T2 |
| Buccofacial movements | 30 | 30 | **29** | 30 | 30 | 30 | 30 | **29** | **27** |
| Characteristic A: improved performance when imitating |  | 0 | 0 | 0 | 0 | 0 | 0 | 0 | 0 |
| Characteristic B: articulatory groping |  | 1 | 1 | 0 | 0 | 0 | 0 | 1 | **3** |
| Articulation consonants and vocals | 30 | **28** | **27** | 30 | 30 | 30 | 30 | 30 | 30 |
| C1: inconsistent production |  | 1 | **3** | 0 | 0 | 0 | 0 | 0 | 0 |
| C2: worse performance consonants than vocals |  | 2 | **3** | 0 | 0 | 0 | 0 | 0 | 0 |
| Diadochokinesis |  | 94 | **87** | 177 | 164 | 178 | 181 | **75** | **53** |
| C3: worse performance alternating sequences than sequential sequences |  | 0.99 | 0.90 | 0.99 | 0.91 | 0.92 | 0.95 | **0.55** | **0.55** |
| C4: articulatory groping |  | **Yes** | **Yes** | No | No | No | No | **Yes** | **Yes** |
| Articulation words | 264 | 247 | 241 | 264 | 263 | 264 | 264 | 264 | 252 |
| C5: initiation problems |  | **0.27** | **0.72** | 0 | 0 | 0 | 0 | 0 | 0 |
| C6: syllable segmentations |  | **0.83** | **1** | 0 | 0 | 0 | 0 | 0 | **0.5** |
| C7: cluster segmentations |  | **0.2** | 0 | 0 | 0 | 0 | 0 | 0 | 0 |
| C8: articulation complexity effect |  | 0.3 | **1.33** | 0 | 0 | 0 | 0 | 0 | 0 |
| Buccofacial apraxia |  | No | No | No | No | No | No | No | **Yes** |
| Apraxia of speech |  | **Yes** | **Yes** | No | No | No | No | No | **Yes** |

Abbreviations: NFV = nonfluent variant; LV = logopenic variant; SV = semantic variant; PPA-NOS = not otherwise specified; C = characteristic; scores in bold = impaired; T1 = initial assessment; T2 = follow-up assessment.

**Electrophysiological results of the MMN**

| **A – T1**  Fz  F3  **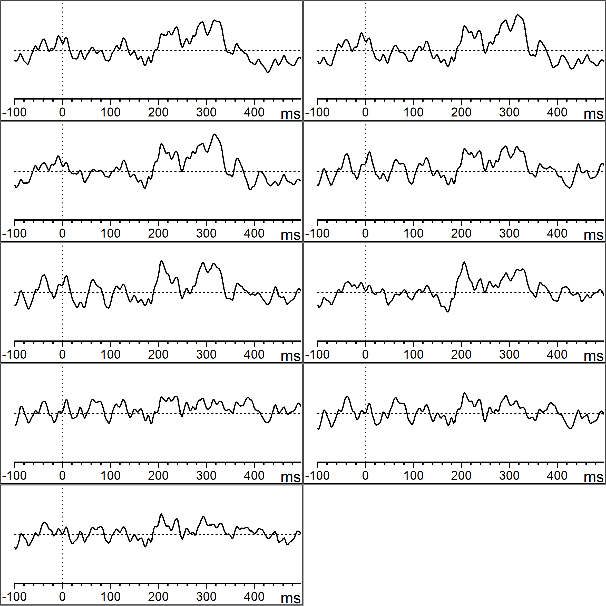**  P4  Pz  P3  C4  Cz  F4  C3  Pz  C4  C3  Fz  P3  F4  F3 | **B – T2**  P4  Pz  P3  C4  Cz  C3  F4  F3  Fz  **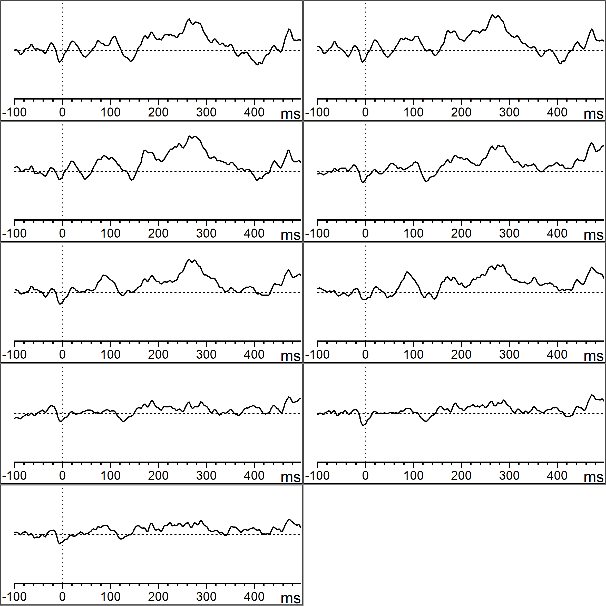**  F3  Fz  F4  P3  C3  C4  Pz |
| --- | --- |
| 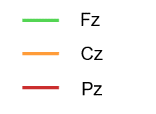**C – T1**  **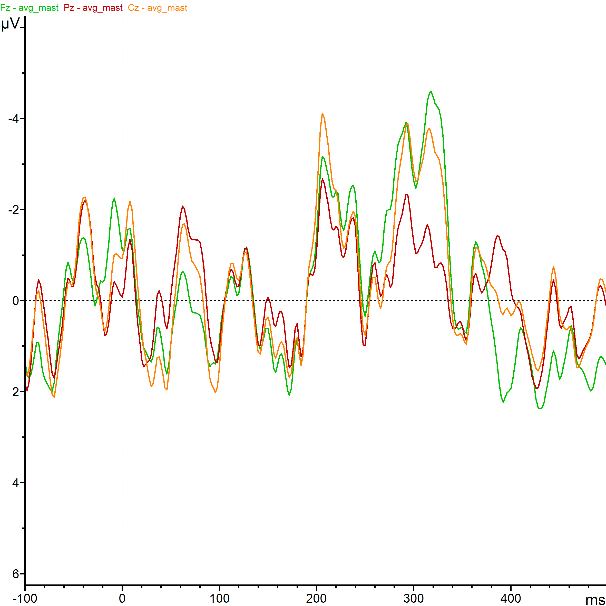** | 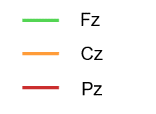**D – T2**  **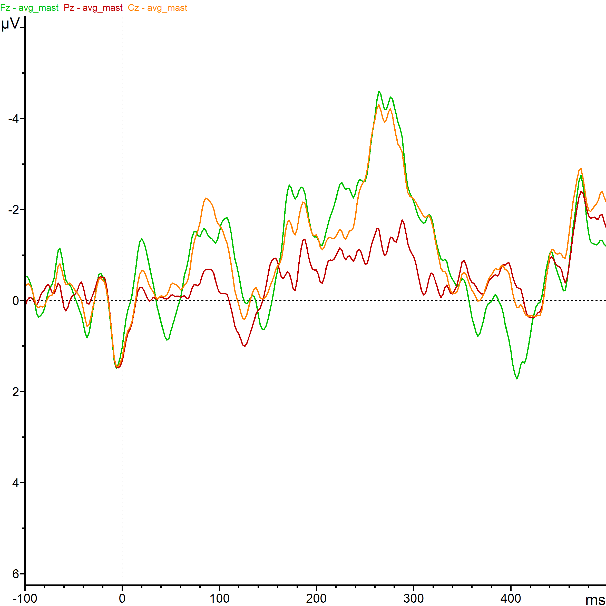** |
| **E – T1**  **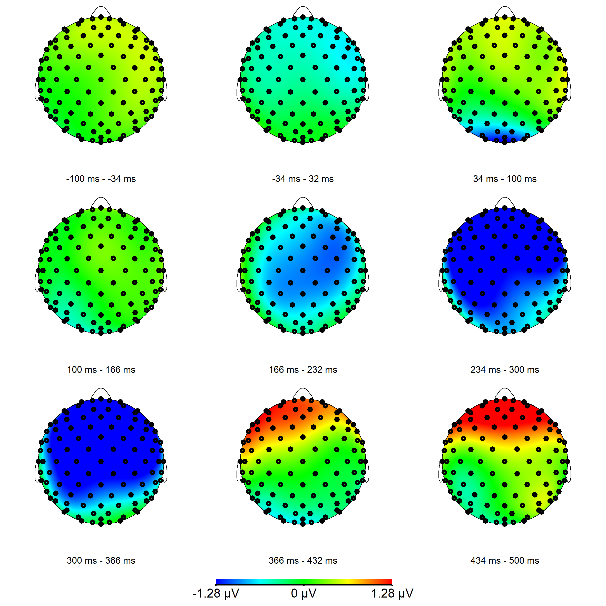** | **F – T2**  **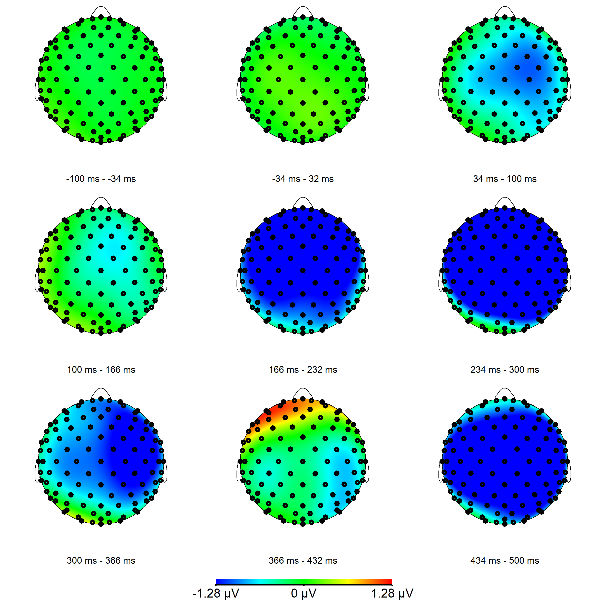** |

**Figure 1.** CASE NFV (SLT). A – B: Difference waveforms (deviant – standard). C – D: Difference waveforms at the midline electrode sites. E – F: Topographic distribution of the difference waveforms.

| **A – T1**  P4  Pz  P3  C4  Cz  C3  F4  Fz  F3  **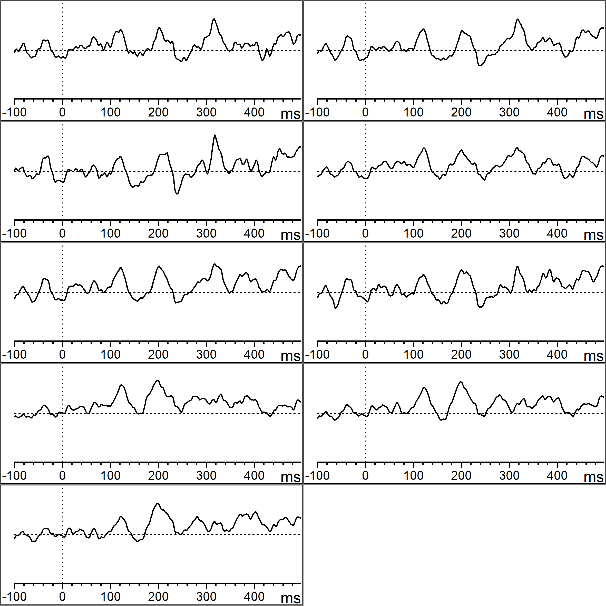**  Pz  C4  C3  Fz  P3  F4  F3 | **B – T1**  P4  Pz  P3  C4  Cz  C3  F4  Fz  F3  **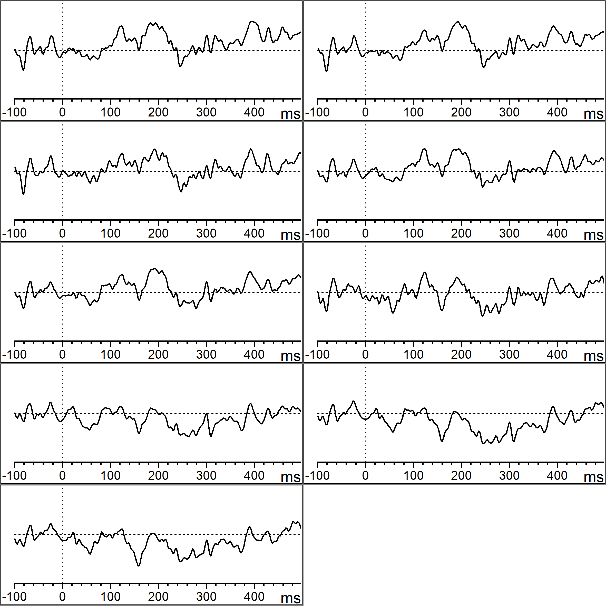**  F3  Fz  F4  P3  C3  C4  Pz |
| --- | --- |
| 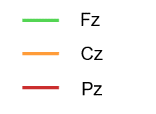**C – T2**  **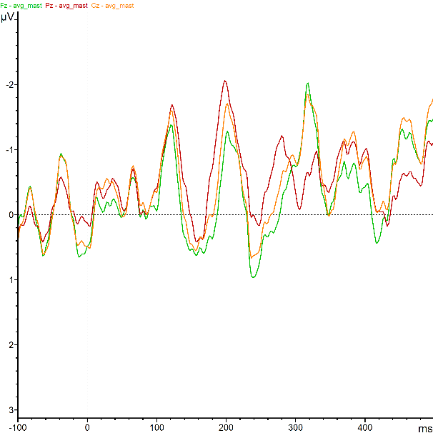** | 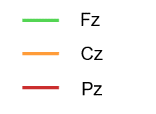**D – T2**  **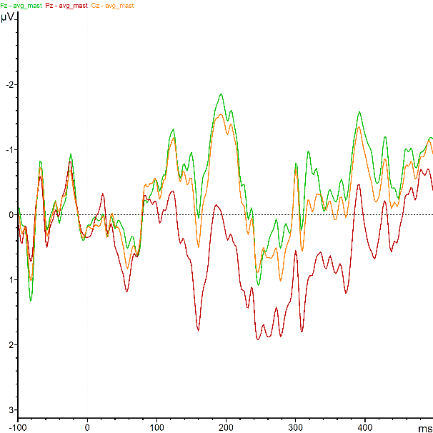** |
| **E – T1**  **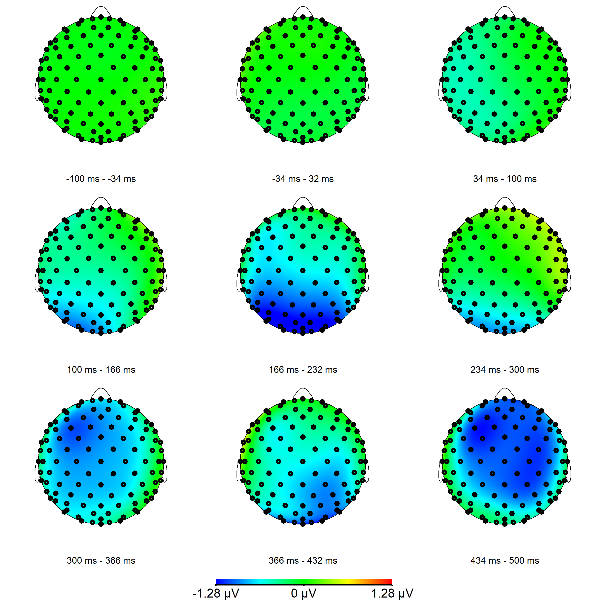** | **F – T2**  **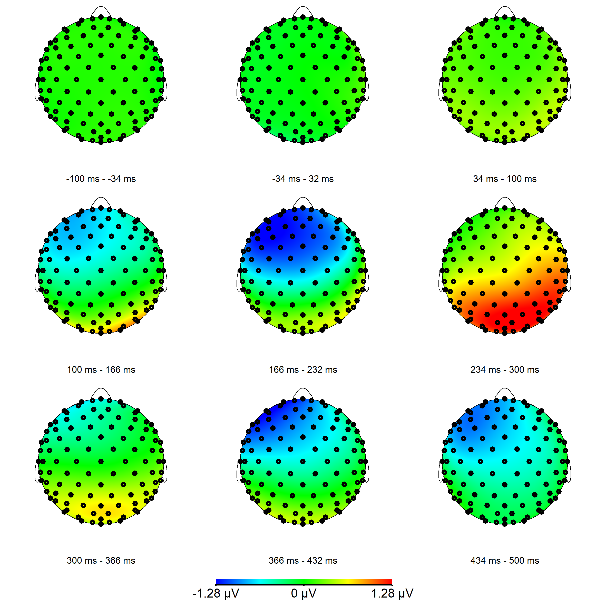** |

**Figure 2.** CASE PPA-NOS (SLT). A – B: Difference waveforms (deviant – standard). C – D: Difference waveforms at the midline electrode sites. E – F: Topographic distribution of the difference waveforms.

| **A – T1**  F3  Fz  F4  C3  Cz  C4  P3  Pz  P4  **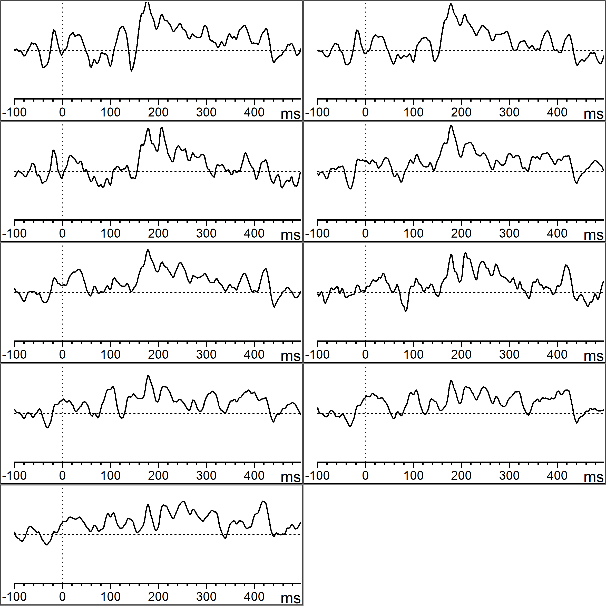**  Pz  C4  C3  Fz  P3  F4  F3 | **B – T2**  F3  Fz  F4  C3  Cz  C4  P3  Pz  P4  **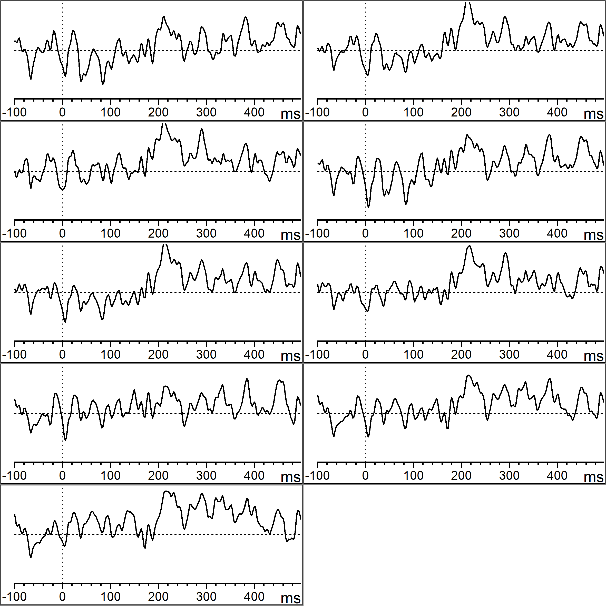**  F3  Fz  F4  P3  C3  C4  Pz |
| --- | --- |
| 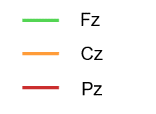**C – T1**  **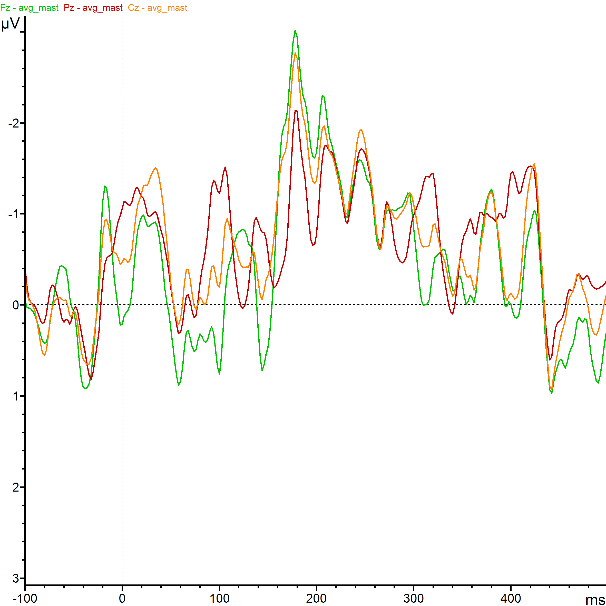** | 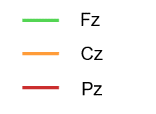**D – T2**  **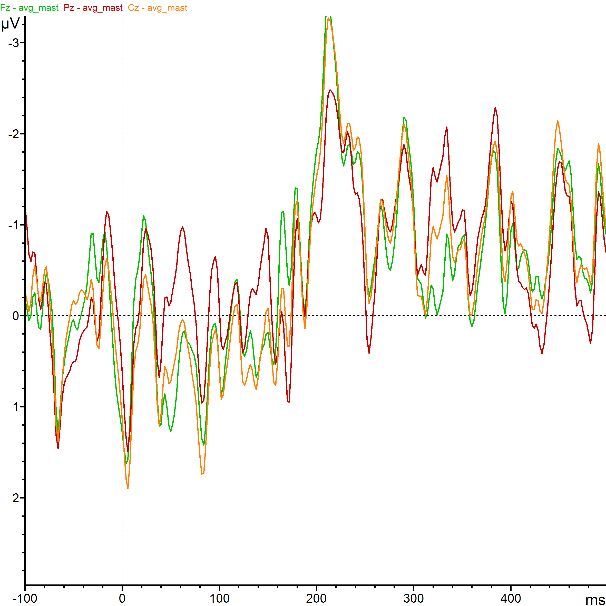** |
| **E – T1**  **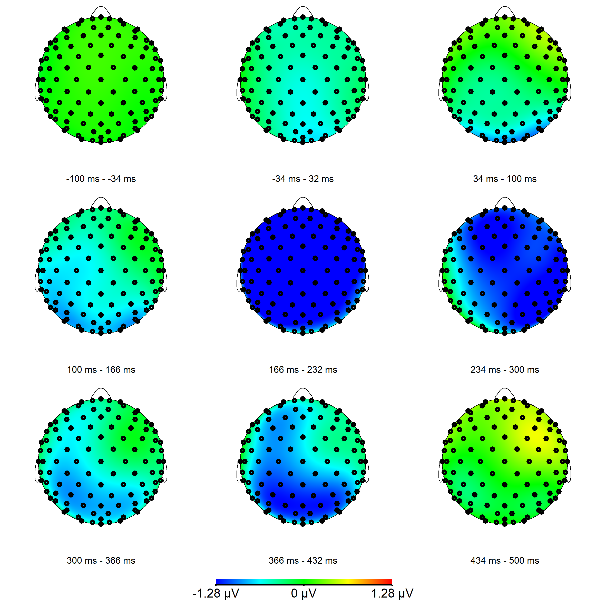** | **F – T2**  **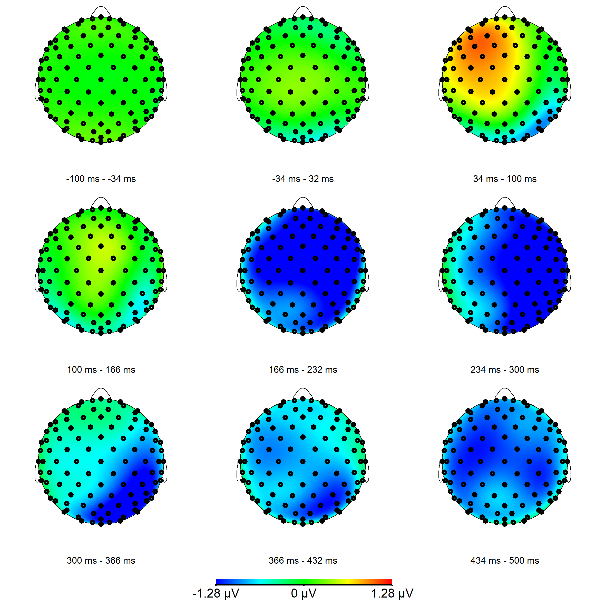** |

**Figure 3.** CASE SV (no SLT). A – B: Difference waveforms (deviant – standard). C – D: Difference waveforms at the midline electrode sites. E – F: Topographic distribution of the difference waveforms.

**Electrophysiological results of the P300**

| **A – T1**  Fz  F3  P3  Pz  P4  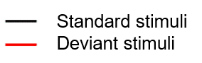**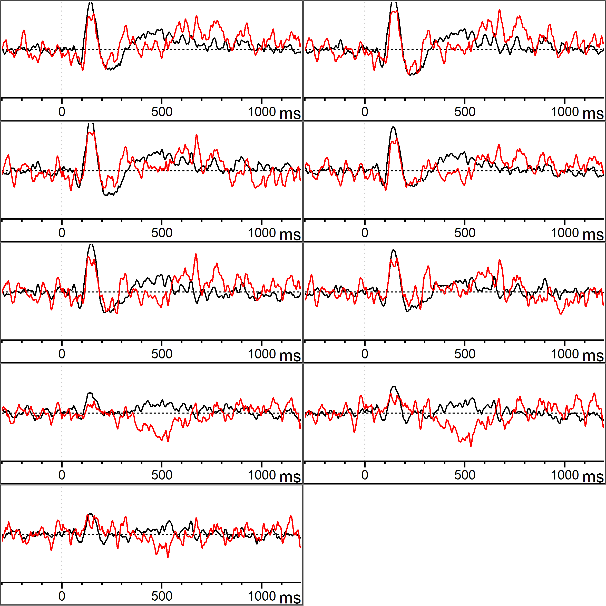**  Cz  C4  C3  F4  Pz  C4  C3  Fz  P3  F4  F3 | **B – T2**  P4  Pz  P3  C4  Cz  C3  F4  Fz  F3  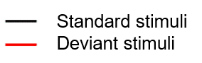**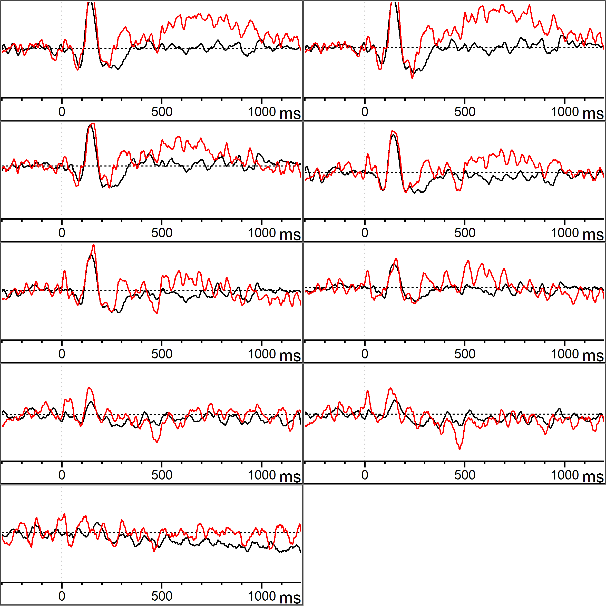**  F3  Fz  F4  P3  C3  C4  Pz |
| --- | --- |
| 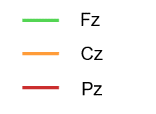**C – T1**  **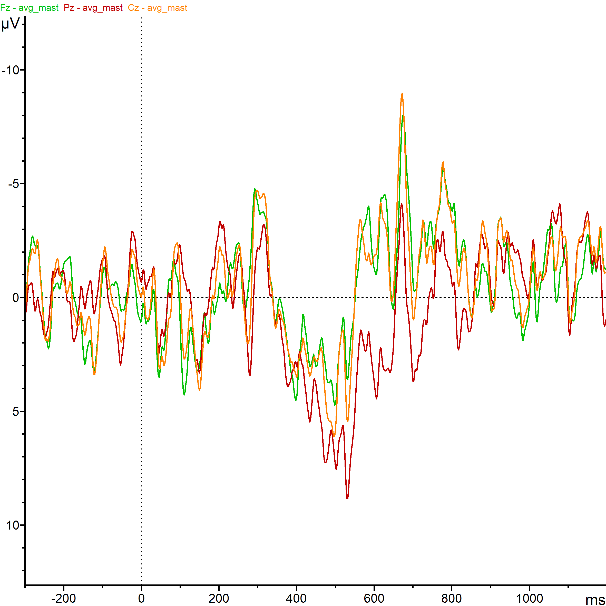** | 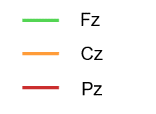**D – T2**  **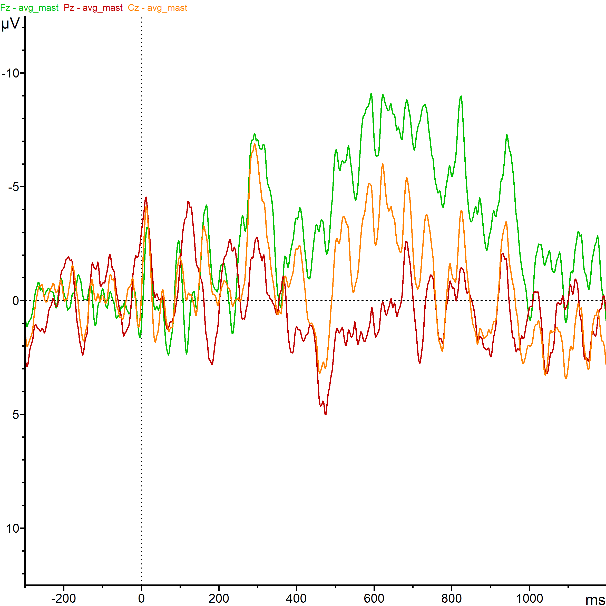** |
| **E – T1**  **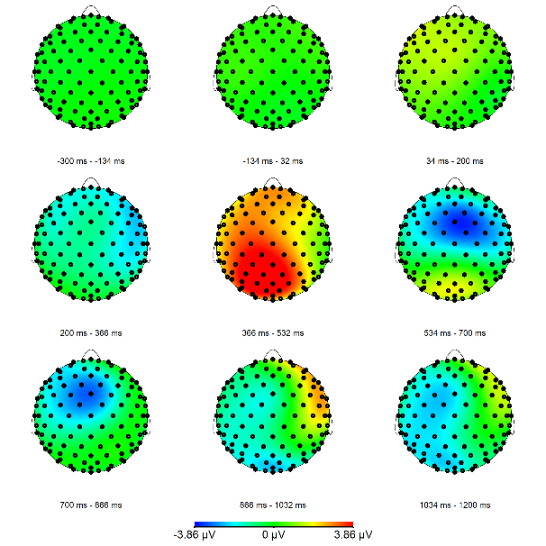** | **F – T2**  **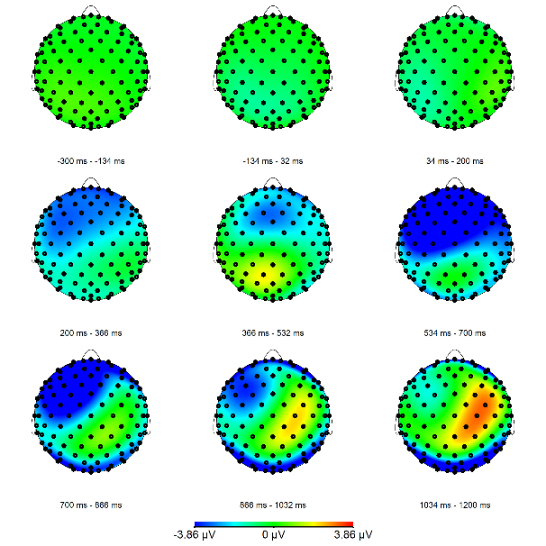** |

**Figure 4.** CASE NFV (SLT). A – B: Average waveforms elicited by the standard and deviant stimuli. C – D: Difference waveforms (deviant – standard) at the midline electrode sites. E – F: Topographic distribution of the difference waveforms.

| **A – T1**  F3  Fz  F4  C3  Cz  C4  P3  Pz  P4  **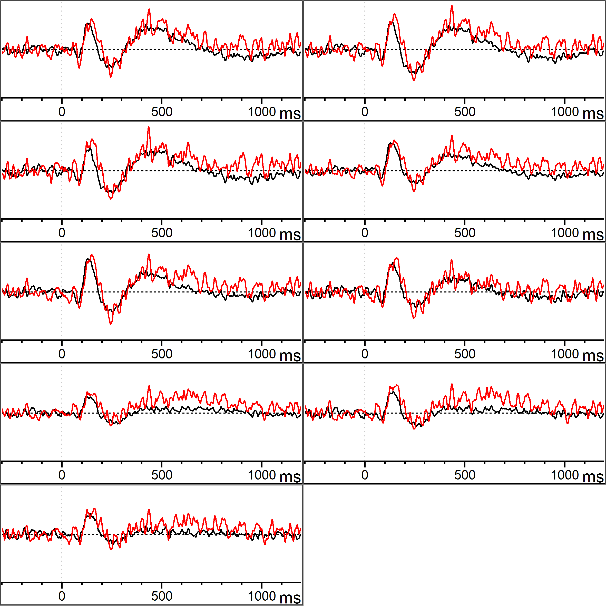** 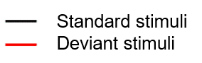  Pz  C4  C3  Fz  P3  F4  F3 | **B – T2**  P4  Pz  P3  C4  Cz  C3  F4  Fz  F3  **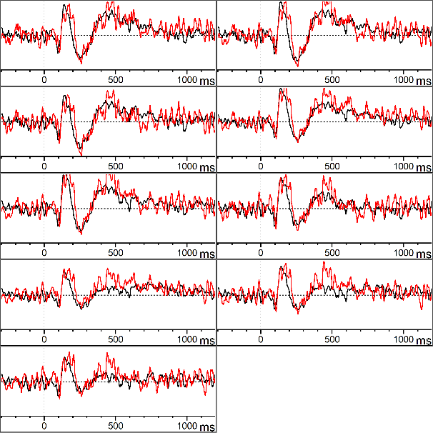**  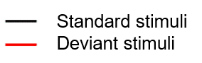  F3  Fz  F4  P3  C3  C4  Pz |
| --- | --- |
| 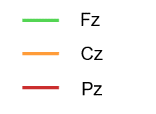**C – T1**  **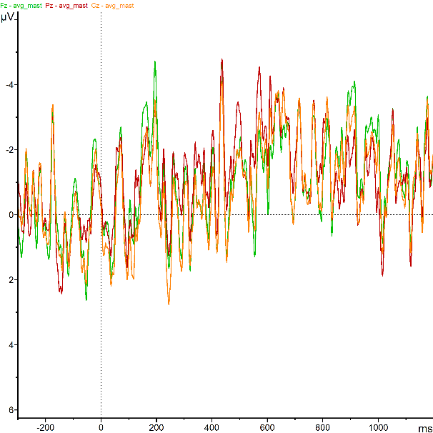** | 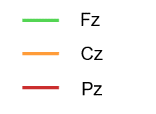**D – T2**  **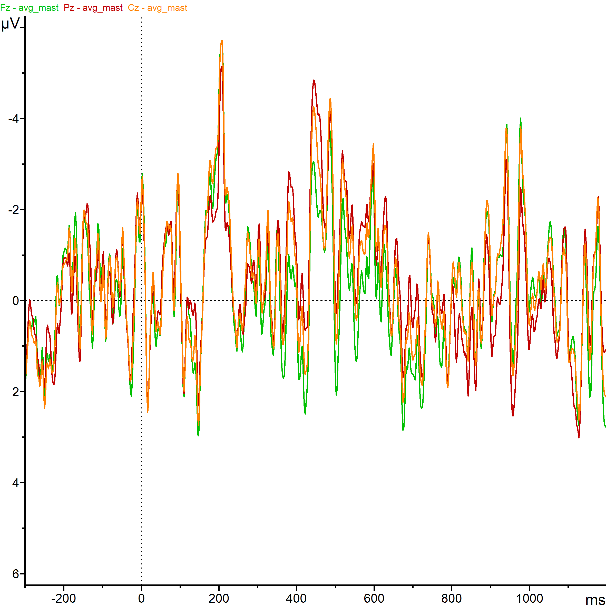** |
| **E – T1**  **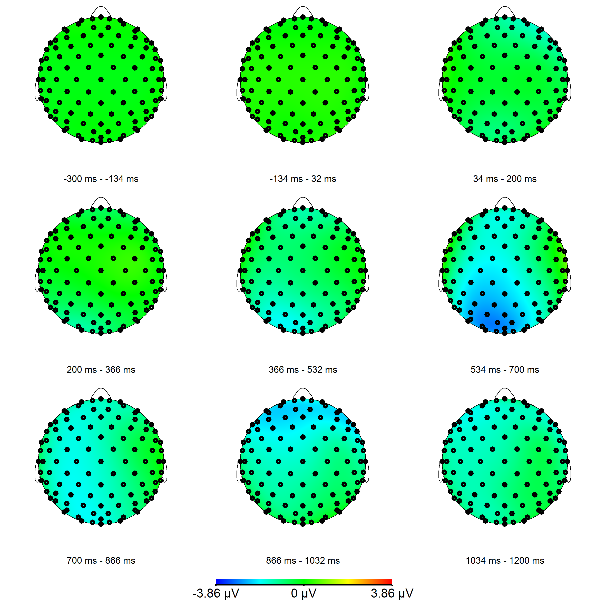** | **F – T2**  **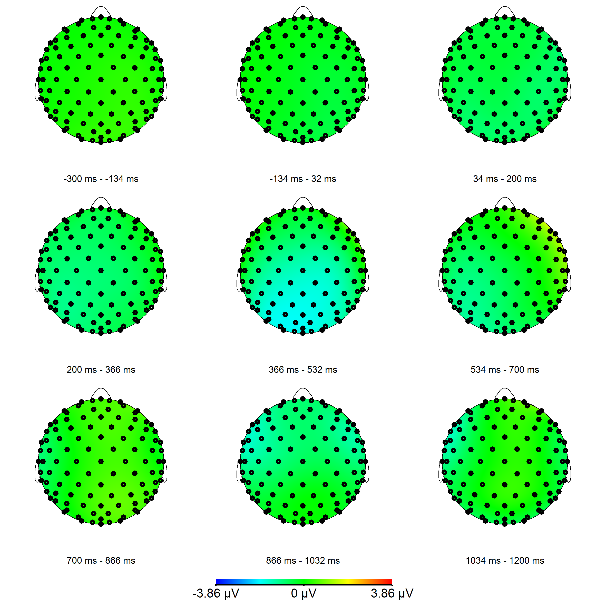** |

**Figure 5.** CASE PPA-NOS (SLT). A – B: Average waveforms elicited by the standard and deviant stimuli. C – D: Difference waveforms (deviant – standard) at the midline electrode sites. E – F: Topographic distribution of the difference waveforms.

| **A – T1**  P4  Pz  P3  C4  Cz  C3  F4  Fz  F3  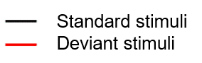**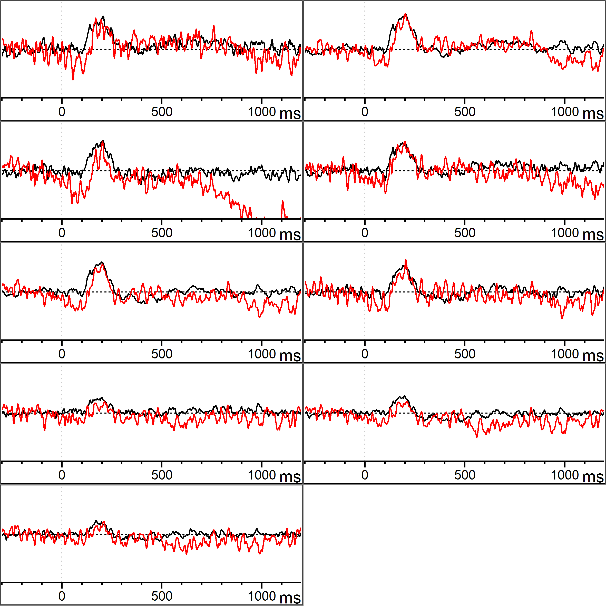**  Pz  C4  C3  Fz  P3  F4  F3 | **B – T2**  P4  Pz  P3  C4  Cz  C3  F4  Fz  F3  **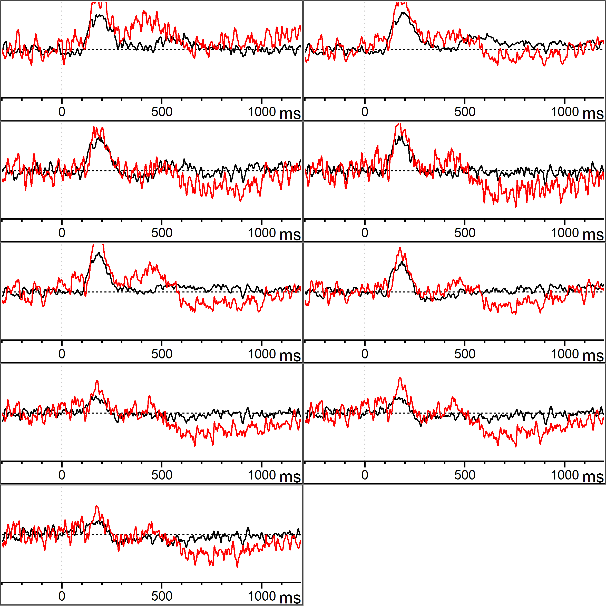** 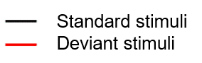  F3  Fz  F4  P3  C3  C4  Pz |
| --- | --- |
| 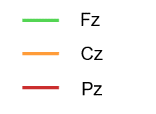**C – T1**  **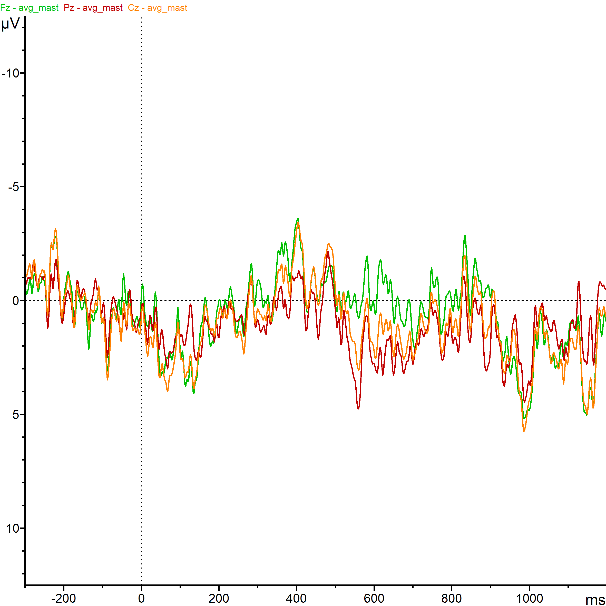** | 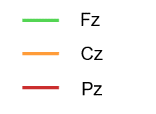**D – T2**  **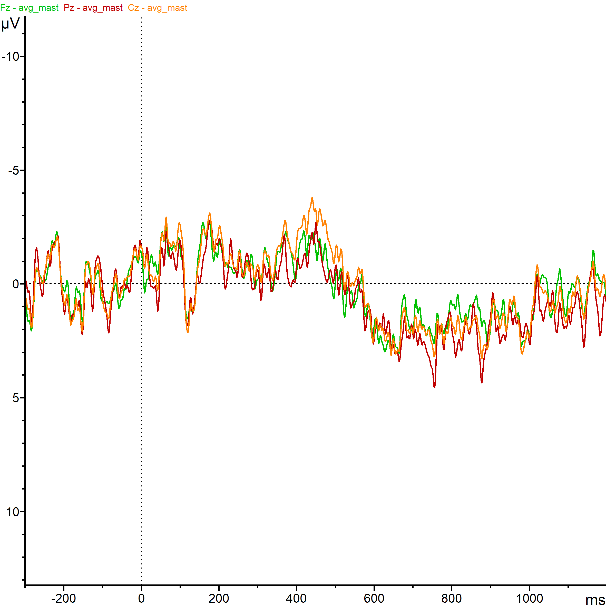** |
| **E – T1**  **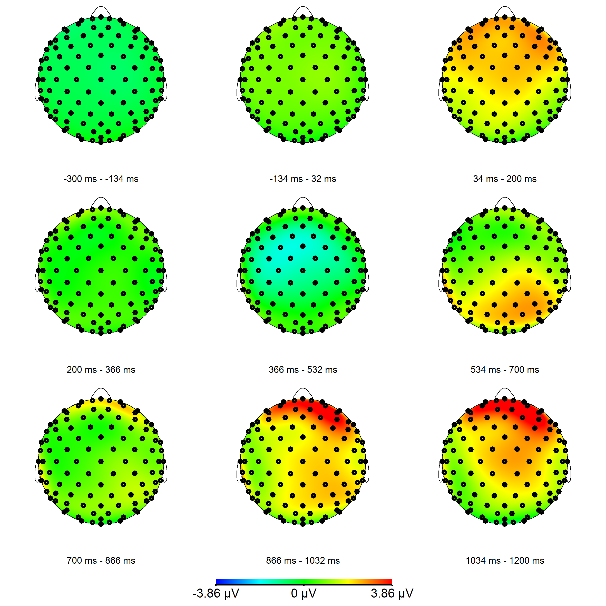** | **F – T2**  **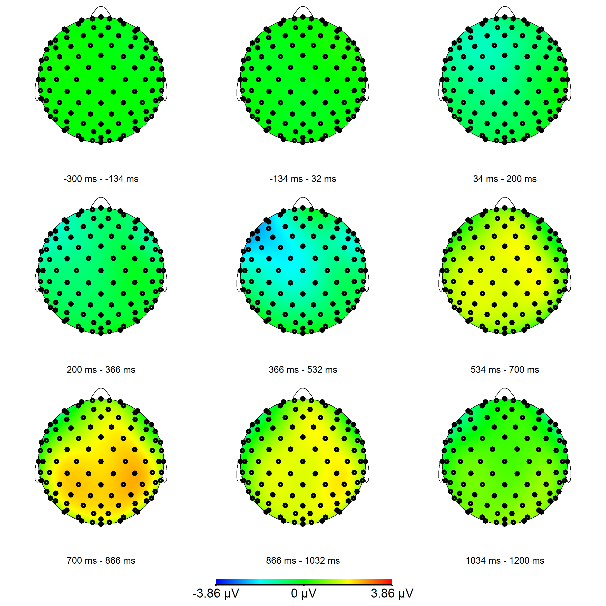** |

**Figure 6.** CASE LV (no SLT). A – B: Average waveforms elicited by the standard and deviant stimuli. C – D: Difference waveforms (deviant – standard) at the midline electrode sites. E – F: Topographic distribution of the difference waveforms.

| **A – T1**  F3  Fz  F4  C3  Cz  C4  P3  Pz  P4  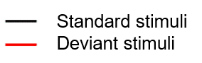**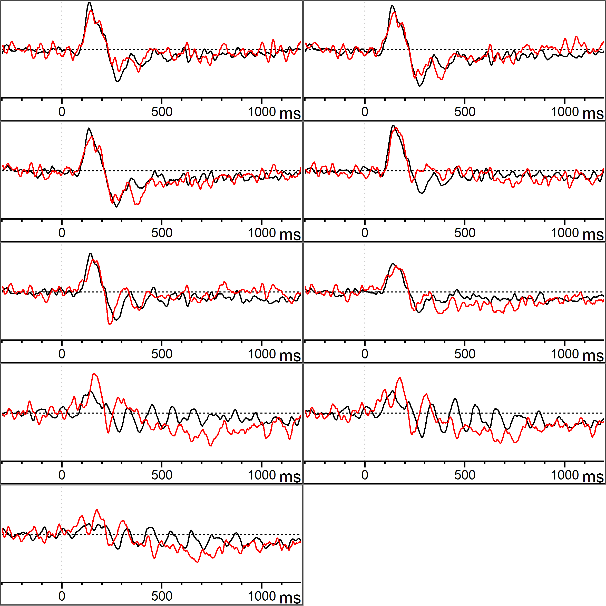**  Pz  C4  C3  Fz  P3  F4  F3 | **B – T2**  F3  Fz  F4  C3  Cz  C4  P3  Pz  P4  **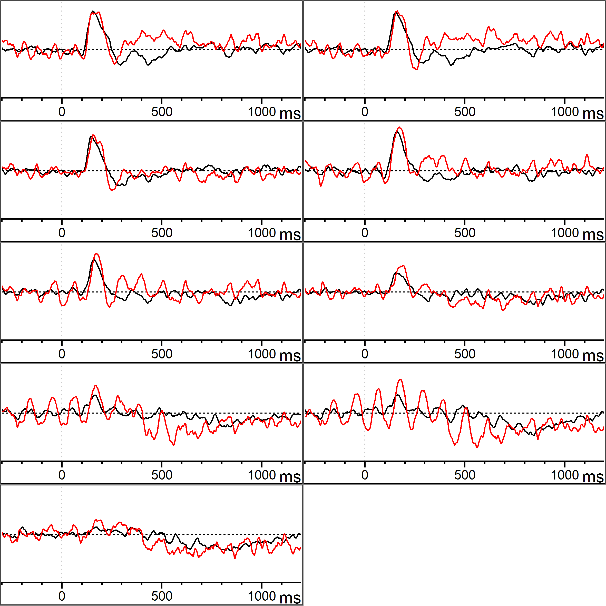** 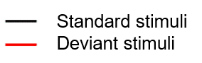  F3  Fz  F4  P3  C3  C4  Pz |
| --- | --- |
| 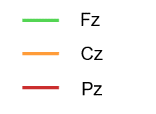**C – T1**  **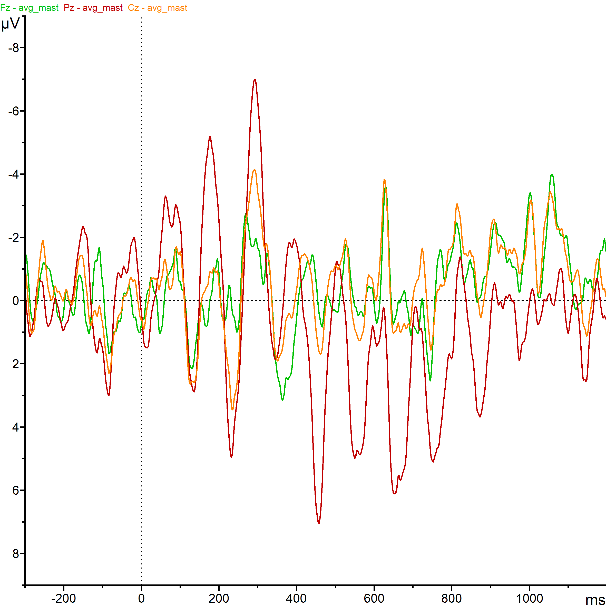** | 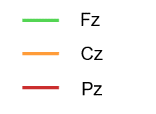**D – T2**  **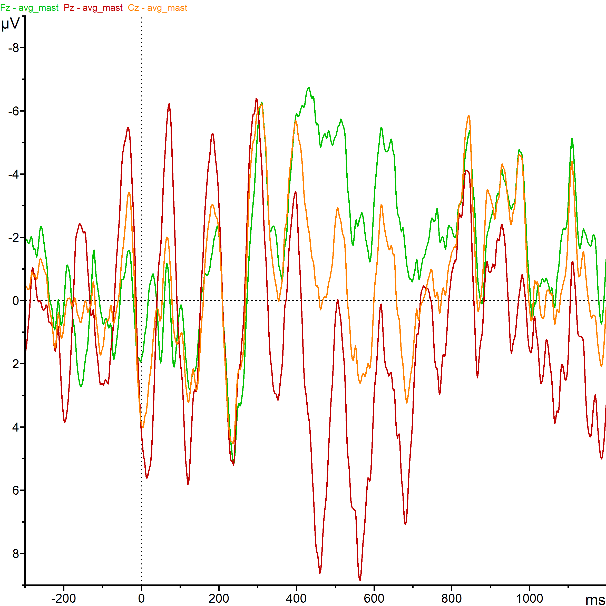** |
| **E – T1**  **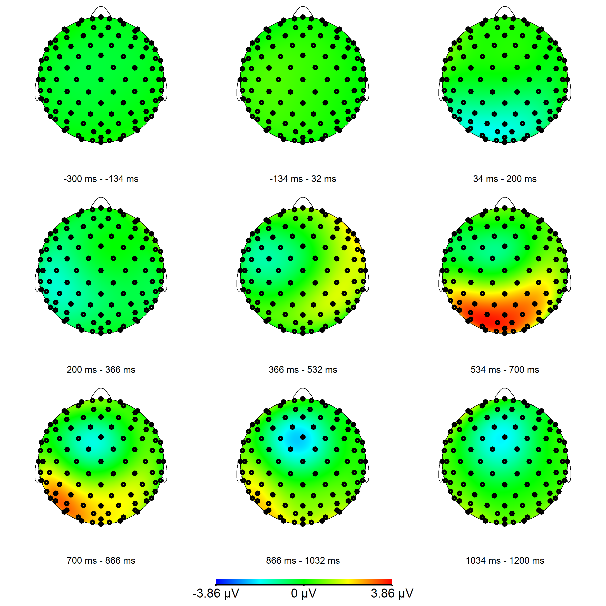** | **F – T2**  **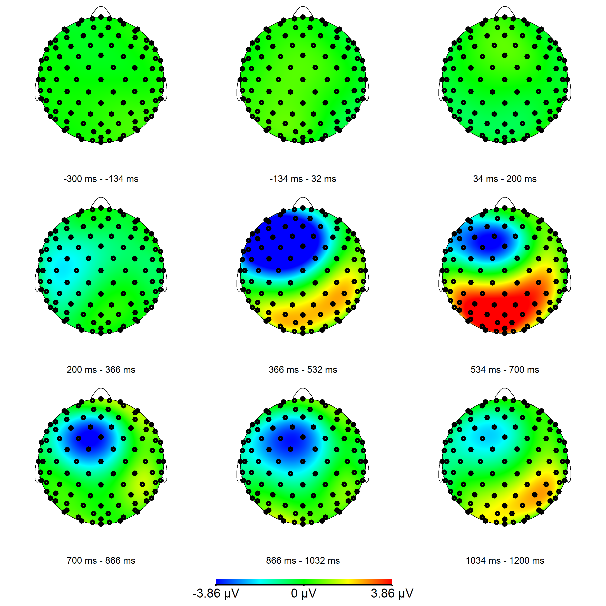** |

**Figure 7.** CASE SV (no SLT). A – B: Average waveforms elicited by the standard and deviant stimuli. C – D: Difference waveforms (deviant – standard) at the midline electrode sites. E – F: Topographic distribution of the difference waveforms.

**Electrophysiological results of the categorical priming paradigm**

| **A – T1**  P4  Pz  P3  C4  Cz  C3  F4  Fz  F3  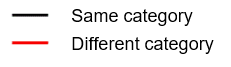**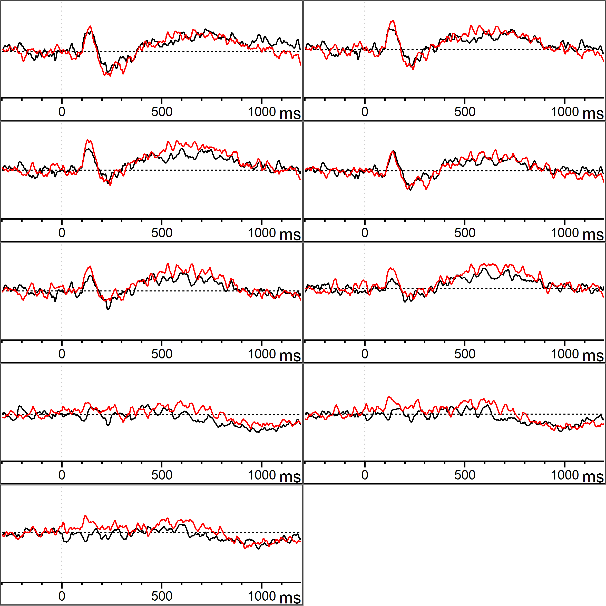**  Pz  C4  C3  Fz  P3  F4  F3 | **B – T2**  P4  Pz  P3  C4  Cz  C3  F4  Fz  F3  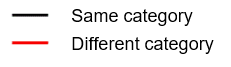**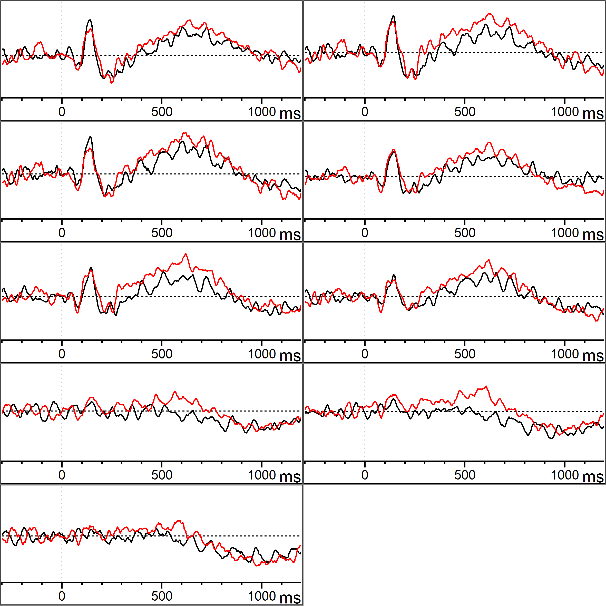** 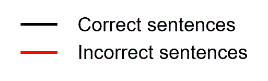  F3  Fz  F4  P3  C3  C4  Pz |
| --- | --- |
| 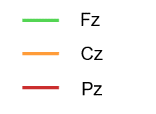**C – T1**  **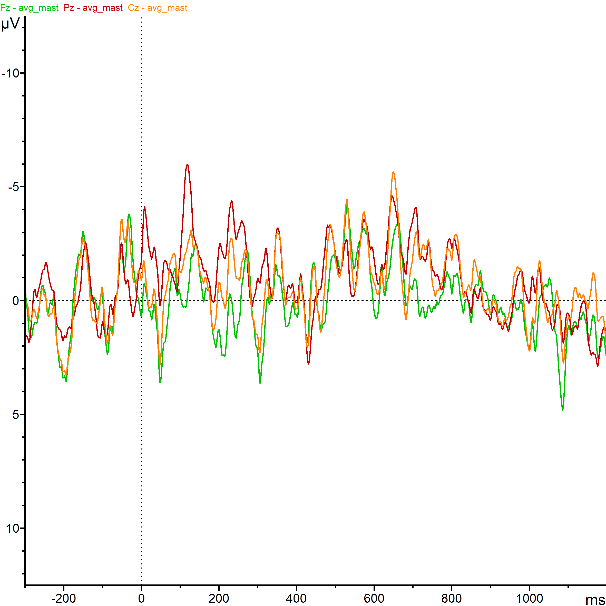** | 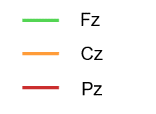**D – T2**  **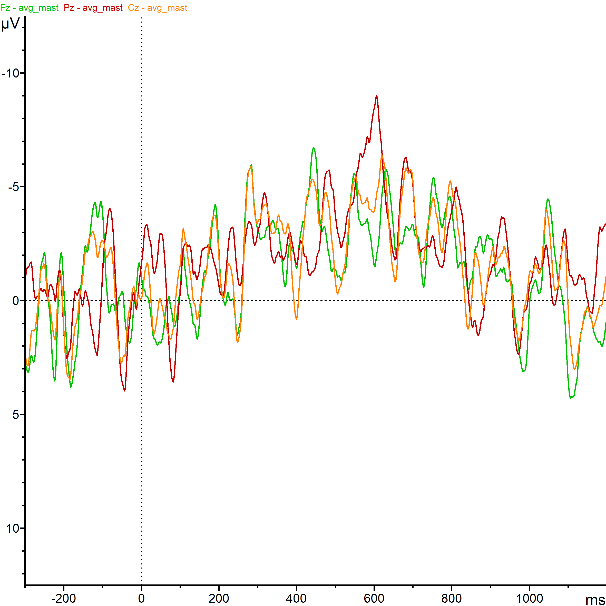** |
| **E – T1**  **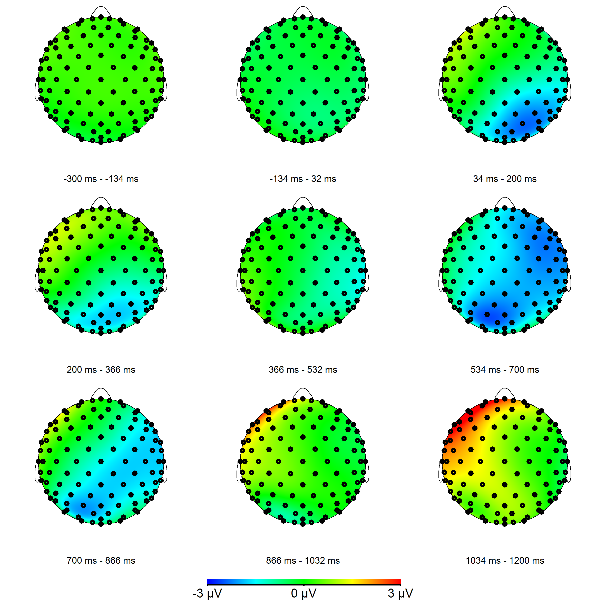** | **F – T2**  **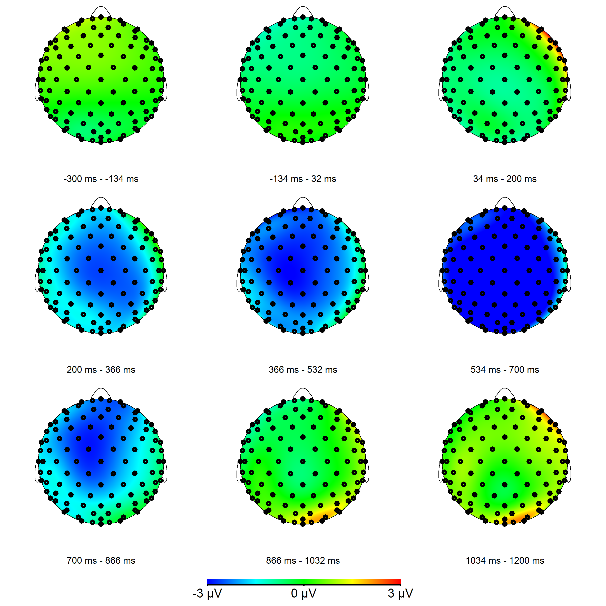** |

**Figure 8.** CASE NFV (SLT). A – B: Average waveforms elicited by the same and different category conditions. C – D: Difference waveforms (different category – same category condition) at the midline electrode sites. E – F: Topographic distribution of the difference waveforms.

| **A – T1**  **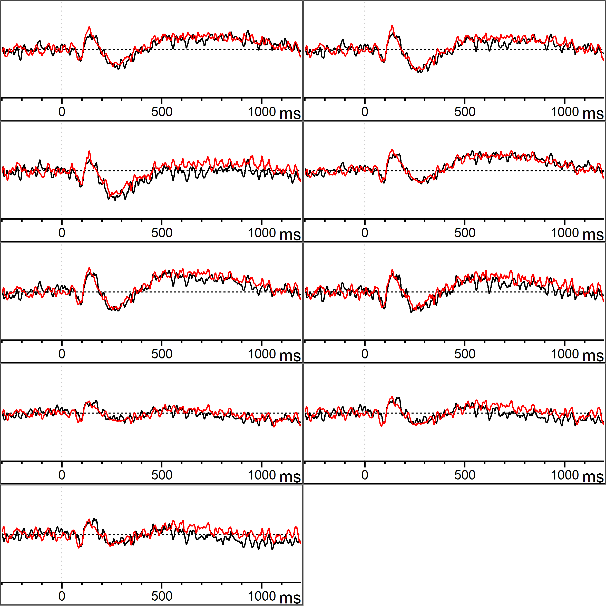**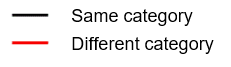  Pz  C4  C3  Fz  P3  F4  F3 | **B – T2**  P4  Pz  P3  C4  Cz  C3  F4  Fz  F3  **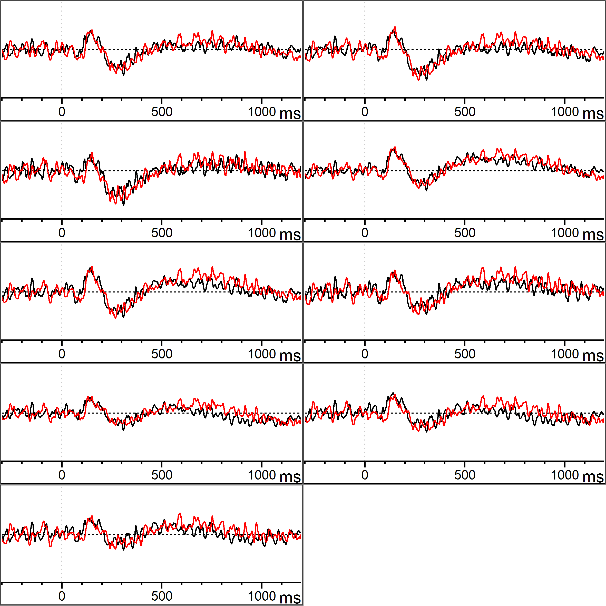**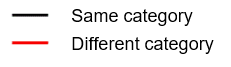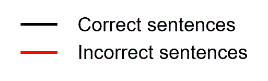  F3  Fz  F4  P3  C3  C4  Pz |
| --- | --- |
| 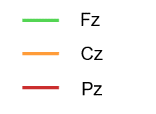**C – T1**  F3  Fz  F4  C3  Cz  C4  P3  Pz  P4  **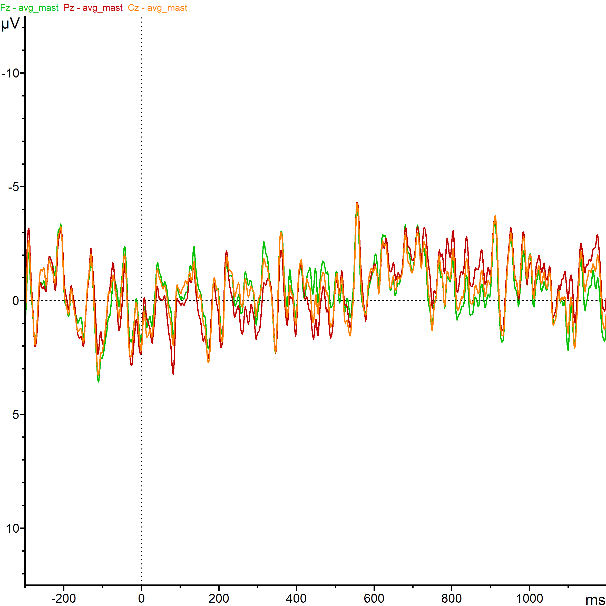** | 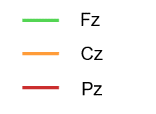**D – T2**  **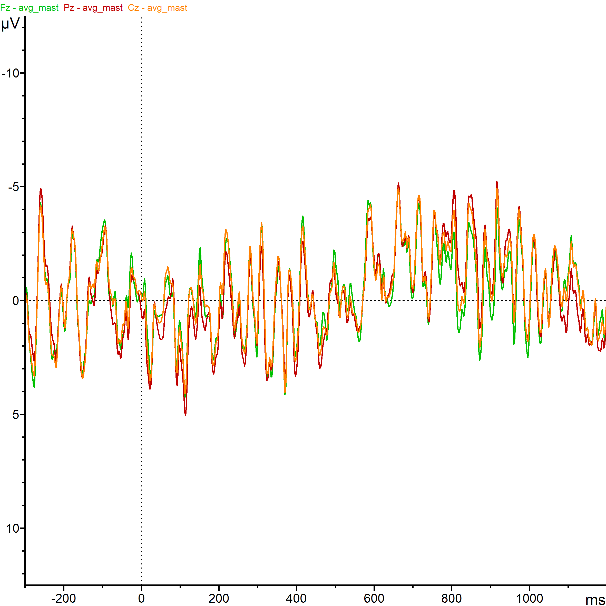** |
| **E – T1**  **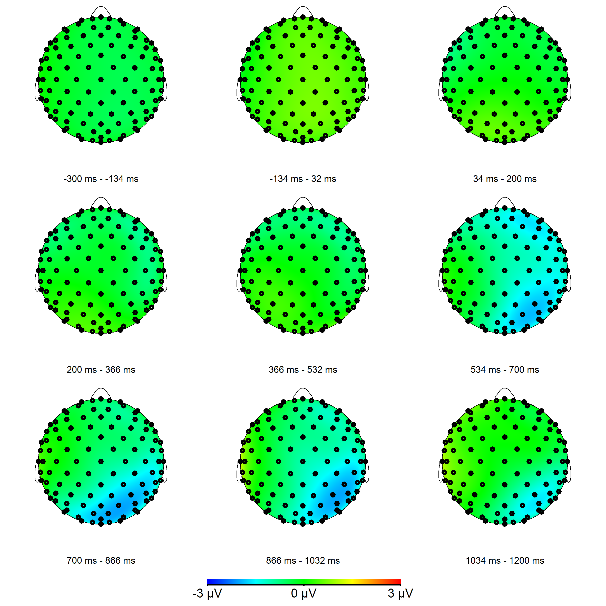** | **F – T2**  **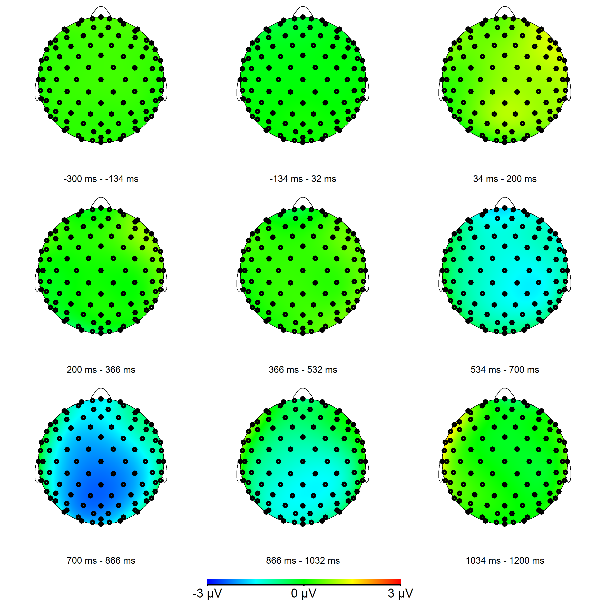** |

**Figure 9.** CASE PPA-NOS (SLT). A – B: Average waveforms elicited by the same and different category conditions. C – D: Difference waveforms (different category – same category condition) at the midline electrode sites. E – F: Topographic distribution of the difference waveforms.

| **A – T1**  P4  Pz  P3  C4  Cz  C3  F4  Fz  F3  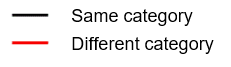 **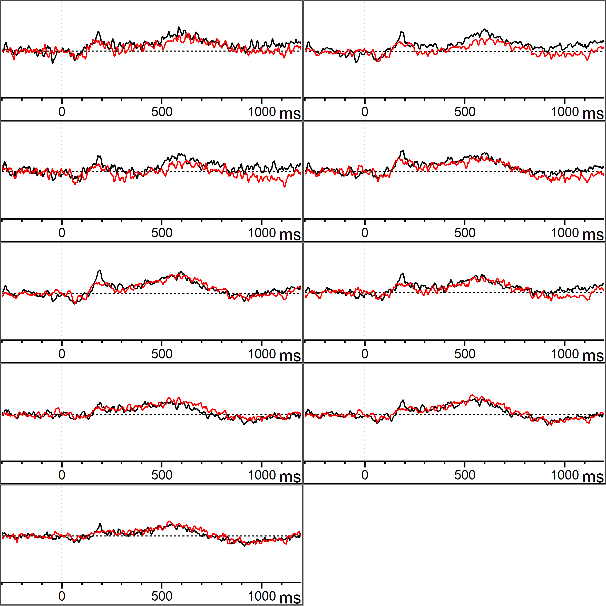**  Pz  C4  C3  Fz  P3  F4  F3 | **B – T2**  F3  Fz  F4  C3  Cz  C4  P3  Pz  P4  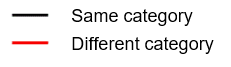 **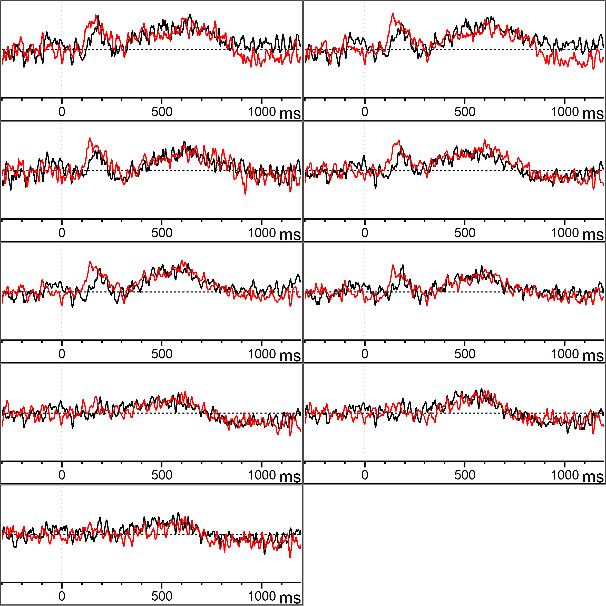** 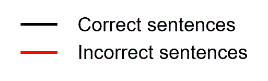  F3  Fz  F4  P3  C3  C4  Pz |
| --- | --- |
| 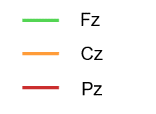**C – T1**  **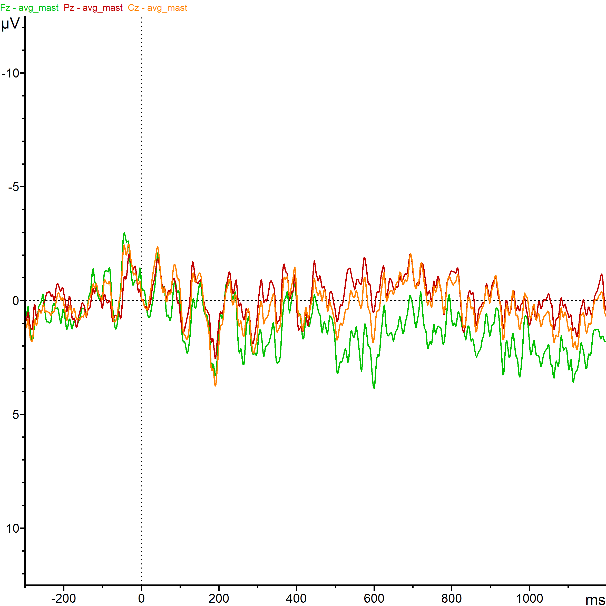** | 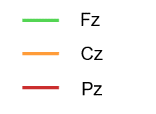**D – T2**  **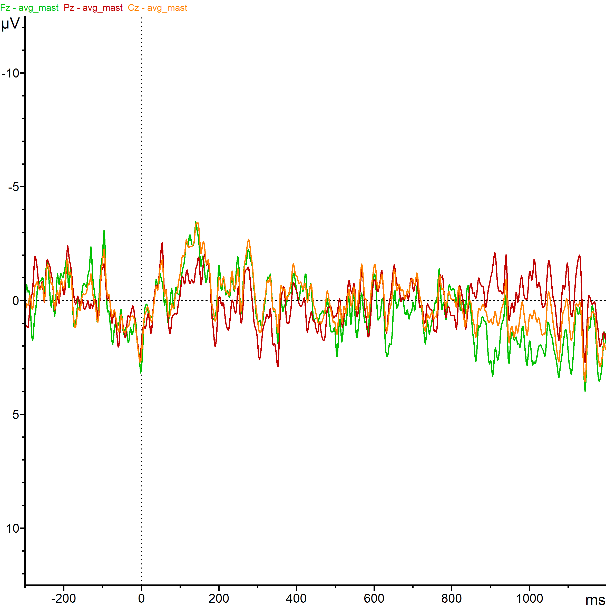** |
| **E – T1**  **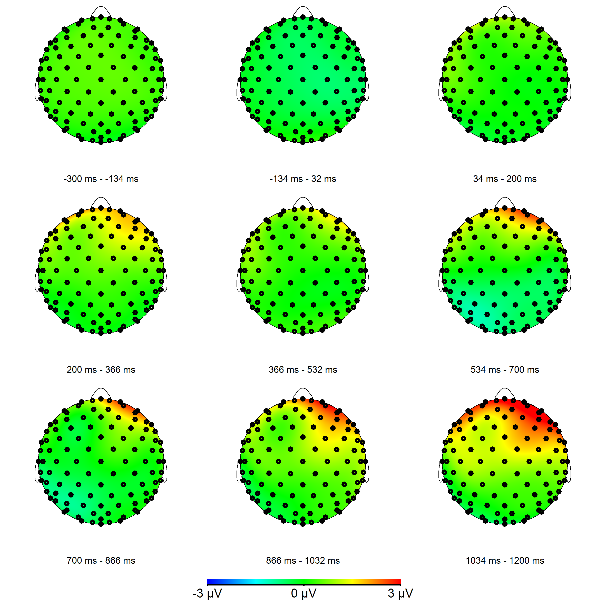** | **F – T2**  **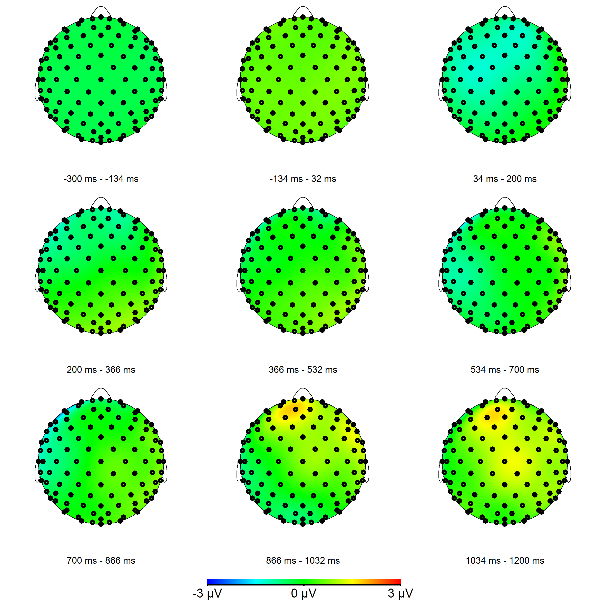** |

**Figure 10.** CASE LV (no SLT). A – D: Average waveforms elicited by the same and different category conditions. B – E: Difference waveforms (different category – same category condition) at the midline electrode sites. C – F: Topographic distribution of the difference waveforms.

| **A – T1**  P4  Pz  P3  C4  Cz  C3  F4  Fz  F3  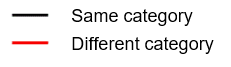 **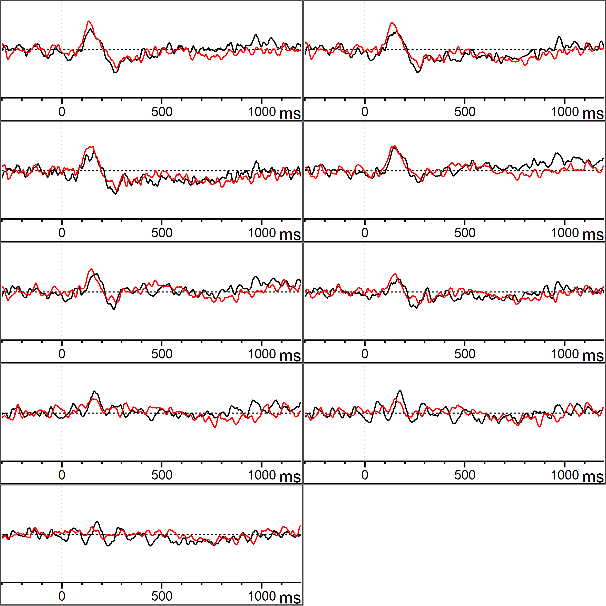**  Pz  C4  C3  Fz  P3  F4  F3 | **B – T2**  P4  Pz  P3  C4  Cz  C3  F4  Fz  F3  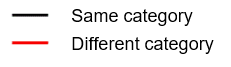  F3  Fz  F4  P3  C3  C4  Pz |
| --- | --- |
| **C – T1** | **D – T2** |
| **E – T1** | **F – T2** |

**Figure 11.** CASE SV (no SLT). A – B: Average waveforms elicited by the same and different category conditions. C – D: Difference waveforms (different category – same category condition) at the midline electrode sites. E – F: Topographic distribution of the difference waveforms.

**Electrophysiological results of the semantic anomaly paradigm**

| **A – T1**  P4  Pz  P3  C4  Cz  C3  F4  Fz  F3    Pz  C4  C3  Fz  P3  F4  F3 | **B – T2**  P4  Pz  P3  C4  Cz  C3  F4  Fz  F3    F3  Fz  F4  P3  C3  C4  Pz |
| --- | --- |
| **C – T1** | **D – T2** |
| **E – T1** | **F– T2** |

**Figure 12.** CASE NFV (SLT). A – B: Average waveforms elicited by the correct and incorrect sentences. C – D: Difference waveforms (incorrect – correct condition) at the midline electrode sites. E – F: Topographic distribution of the difference waveforms.

| **A – T1**  P4  Pz  P3  C4  Cz  C3  F4  Fz  F3    Pz  C4  C3  Fz  P3  F4  F3 | **B – T2**  F3  Fz  F4  C3  Cz  C4  P3  Pz  P4    F3  Fz  F4  P3  C3  C4  Pz |
| --- | --- |
| **C – T1** | **D – T2** |
| **E – T1** | **F – T2** |

**Figure 13.** CASE PPA-NOS (SLT). A – B: Average waveforms elicited by the correct and incorrect sentences. C – D: Difference waveforms (incorrect – correct condition) at the midline electrode sites. E – F: Topographic distribution of the difference waveforms.

| **A – T1**  P4  Pz  P3  C4  Cz  C3  F4  Fz  F3    Pz  C4  C3  Fz  P3  F4  F3 | **B – T2**  P4  Pz  P3  C4  Cz  C3  F4  Fz  F3    F3  Fz  F4  P3  C3  C4  Pz |
| --- | --- |
| **C – T1** | **D – T2** |
| **E – T1** | **F – T2** |

**Figure 14.** CASE LV (no SLT). A – B: Average waveforms elicited by the correct and incorrect sentences. C – D: Difference waveforms (incorrect – correct condition) at the midline electrode sites. E – F: Topographic distribution of the difference waveforms.

**Electrophysiological results of the P600 paradigm**

| **A – T1**  F3  Fz  F4  C3  Cz  C4  P3  Pz  P4    Pz  C4  C3  Fz  P3  F4  F3 | **B – T2**  P4  Pz  P3  C4  Cz  C3  F4  Fz  F3    F3  Fz  F4  P3  C3  C4  Pz |
| --- | --- |
| **C – T1** | **D – T2** |
| **E – T1** | **F – T2** |

**Figure 15.** CASE NFV (SLT). A – B: Average waveforms elicited by the correct and incorrect sentences. C – D: Difference waveforms (incorrect – correct condition) at the midline electrode sites. E – F: Topographic distribution of the difference waveforms.

| **A – T1**  P4  Pz  P3  C4  Cz  C3  F4  Fz  F3    Pz  C4  C3  Fz  P3  F4  F3 | **B – T2**  P4  Pz  P3  C4  Cz  C3  F4  Fz  F3    F3  Fz  F4  P3  C3  C4  Pz |
| --- | --- |
| **C – T1** | **D – T2** |
| **E – T1** | **F – T2** |

**Figure 16.** CASE PPA-NOS (SLT). A – B: Average waveforms elicited by the correct and incorrect sentences. C – D: Difference waveforms (incorrect – correct condition) at the midline electrode sites. E – F: Topographic distribution of the difference waveforms.

| **A – T1**  P4  Pz  P3  C4  Cz  C3  F4  Fz  F3    Pz  C4  C3  Fz  P3  F4  F3 | **B – T2**  F3  Fz  F4  C3  Cz  C4  P3  Pz  P4    F3  Fz  F4  P3  C3  C4  Pz |
| --- | --- |
| **C – T1** | **D – T2** |
| **E – T1** | **F – T2** |

**Figure 17.** CASE LV (no SLT). A – B: Average waveforms elicited by the correct and incorrect sentences. C – D: Difference waveforms (incorrect – correct condition) at the midline electrode sites. E – F: Topographic distribution of the difference waveforms.

# Supplementary Tables of the healthy control group

**Table 23.** The descriptive statistics of the mean amplitudes (in μV) and onset latencies (in ms) of the MMN difference waves (deviant – standard condition) for the control group at the frontal, central, parietal, left, midline, and right electrode sites.

|  | Electrode | Mean | SD | 95% CI | Min | Max | IQR |
| --- | --- | --- | --- | --- | --- | --- | --- |
| Mean amplitude 150-250ms | F | -1.60 | 0.76 | -1.31 – -1.88 | -1.88 | -3.23 | 0.12 |
|  | C | -1.34 | 0.74 | -1.07 – -1.62 | -1.62 | -3.35 | 0.17 |
|  | P | -0.78 | 0.60 | -0.55 – -1.00 | -1.00 | -1.82 | 0.34 |
|  | L | -1.19 | 0.57 | -0.97 – -1.40 | -1.40 | -2.40 | -0.09 |
|  | M | -1.33 | 0.72 | -1.07 – -1.60 | -1.60 | -2.82 | 0.25 |
|  | R | -1.20 | 0.69 | -0.94 – -1.46 | -1.46 | -2.69 | 0.19 |
| Mean amplitude 250-350ms | F | -1.03 | 0.81 | -0.73 – -1.34 | -1.34 | -3.07 | 0.97 |
|  | C | -0.85 | 0.80 | -0.55 – -1.15 | -1.15 | -2.83 | 0.93 |
|  | P | -0.49 | 0.66 | -0.24 – -0.74 | -0.74 | -1.61 | 1.11 |
|  | L | -0.77 | 0.63 | -0.54 – -1.01 | -1.01 | -2.16 | 0.94 |
|  | M | -0.82 | 0.79 | -0.52 – -1.11 | -1.11 | -2.37 | 0.83 |
|  | R | -0.79 | 0.73 | -0.51 – -1.06 | -1.06 | -2.50 | 0.76 |
| Mean amplitude 350-450ms | F | -0.32 | 0.82 | -0.01 – -0.62 | -0.62 | -2.56 | 1.84 |
|  | C | -0.30 | 0.81 | 0.00 – -0.60 | -0.60 | -2.65 | 1.66 |
|  | P | -0.37 | 0.70 | -0.11 – -0.63 | -0.63 | -1.75 | 1.12 |
|  | L | -0.28 | 0.67 | -0.03 – -0.53 | -0.53 | -1.80 | 1.70 |
|  | M | -0.35 | 0.80 | -0.05 – -0.65 | -0.65 | -2.05 | 1.56 |
|  | R | -0.36 | 0.75 | -0.08 – -0.64 | -0.64 | -2.49 | 1.37 |
| Onset latency | F | 208.69 | 16.46 | 202.54 – 214.84 | 202.54 | 187.33 | 241.33 |
|  | C | 211.40 | 16.04 | 205.41 – 217.39 | 205.41 | 187.33 | 247.33 |
|  | P | 216.44 | 20.99 | 208.61 – 224.28 | 208.61 | 180.00 | 267.33 |
|  | L | 212.82 | 16.81 | 206.54 – 219.10 | 206.54 | 182.67 | 244.67 |
|  | M | 211.71 | 17.81 | 205.06 – 218.36 | 205.06 | 178.67 | 250.67 |
|  | R | 212.00 | 15.94 | 206.05 – 217.95 | 206.05 | 180.67 | 252.67 |

Abbreviations: F = frontal, C = central, P = parietal, L = left, M = midline, R = right, SD = standard deviation, CI = confidence interval, Min = minimum, Max = maximum, IQR = interquartile range.

**Table 24.** The descriptive statistics of the mean amplitudes (in μV) and onset latencies (in ms) of the P300 difference waves (deviant – standard condition) for the control group at the frontal, central, parietal, left, midline, and right electrode sites.

|  | Electrode | Mean | SD | 95% CI | Min | Max | IQR |
| --- | --- | --- | --- | --- | --- | --- | --- |
| Mean amplitude 350-550ms | F | 1.27 | 2.98 | 0.16 – 2.38 | -4.93 | 7.06 | 4.90 |
|  | C | 1.32 | 3.47 | 0.02 – 2.61 | -6.55 | 8.76 | 3.71 |
|  | P | 3.24 | 3.84 | 1.81 – 4.67 | -4.74 | 11.52 | 4.59 |
|  | L | 1.55 | 2.93 | 0.46 – 2.65 | -4.78 | 8.02 | 2.49 |
|  | M | 2.10 | 3.44 | 0.81 – 3.38 | -5.54 | 9.50 | 3.81 |
|  | R | 2.17 | 3.05 | 1.04 – 3.31 | -5.90 | 8.83 | 3.35 |
| Mean amplitude 550-750ms | F | -1.11 | 2.36 | -1.99 – -0.23 | -6.43 | 2.62 | 3.21 |
|  | C | 0.37 | 2.73 | -0.65 – 1.39 | -6.76 | 4.76 | 3.34 |
|  | P | 2.72 | 2.91 | 1.63 – 3.80 | -3.49 | 9.16 | 3.85 |
|  | L | 0.40 | 2.16 | -0.40 – 1.21 | -5.38 | 4.04 | 2.67 |
|  | M | 0.65 | 2.49 | -0.28 – 1.58 | -5.08 | 4.42 | 3.27 |
|  | R | 0.92 | 2.12 | 0.13 – 1.71 | -3.69 | 4.16 | 1.77 |
| Mean amplitude 750-950ms | F | -1.29 | 2.21 | -2.11 – -0.46 | -7.21 | 3.33 | 2.95 |
|  | C | -0.82 | 2.05 | -1.58 – -0.05 | -5.32 | 3.81 | 2.43 |
|  | P | 0.07 | 2.23 | -0.76 – 0.90 | -3.88 | 5.10 | 2.86 |
|  | L | -1.12 | 1.74 | -1.77 – -0.47 | -4.87 | 2.42 | 1.69 |
|  | M | -0.79 | 2.18 | -1.60 – 0.03 | -4.83 | 4.68 | 2.68 |
|  | R | -0.13 | 1.62 | -0.74 – 0.48 | -4.02 | 3.86 | 1.81 |
| Onset latency | F | 491.51 | 121.23 | 446.24 – 536.78 | 365.33 | 834.67 | 98.00 |
|  | C | 472.27 | 69.75 | 446.22 – 498.31 | 352.00 | 674.67 | 100.17 |
|  | P | 494.42 | 80.78 | 464.26 – 524.58 | 367.33 | 818.00 | 54.33 |
|  | L | 487.58 | 85.53 | 455.64 – 519.51 | 363.33 | 818.00 | 72.67 |
|  | M | 489.53 | 88.84 | 456.36 – 522.71 | 365.33 | 833.33 | 84.67 |
|  | R | 481.09 | 63.26 | 457.47 – 504.71 | 366.00 | 676.00 | 50.50 |

Abbreviations: F = frontal, C = central, P = parietal, L = left, M = midline, R = right, SD = standard deviation, CI = confidence interval, Min = minimum, Max = maximum, IQR = interquartile range.

**Table 25.** The descriptive statistics of the mean amplitudes (in μV) and onset latencies (in ms) of the difference waves (different – same category condition) elicited by the categorical priming paradigm for the control group at the frontal, central, parietal, left, midline, and right electrode sites.

|  | Electrode | Mean | SD | 95% CI | Min | Max | IQR |
| --- | --- | --- | --- | --- | --- | --- | --- |
| Mean amplitude 300-500ms | F | -0.69 | 1.19 | -1.13 – -0.25 | -2.46 | 2.86 | 1.44 |
|  | C | -0.52 | 1.00 | -0.90 – -0.15 | -2.85 | 1.74 | 1.46 |
|  | P | -0.51 | 0.94 | -0.86 – -0.15 | -2.19 | 1.25 | 1.70 |
|  | L | -0.61 | 0.96 | -0.97 – -0.25 | -2.70 | 1.55 | 1.22 |
|  | M | -0.67 | 1.15 | -1.10 – -0.24 | -2.86 | 2.90 | 1.38 |
|  | R | -0.44 | 0.94 | -0.79 – -0.09 | -2.01 | 1.71 | 1.58 |
| Mean amplitude 500-700ms | F | -0.78 | 1.36 | -1.29 – -0.27 | -3.45 | 2.08 | 2.15 |
|  | C | -1.53 | 1.21 | -1.99 – -1.08 | -3.98 | 1.49 | 1.56 |
|  | P | -1.92 | 1.28 | -2.39 – -1.44 | -4.33 | 1.01 | 1.46 |
|  | L | -1.34 | 1.17 | -1.77 – -0.90 | -3.86 | 0.70 | 1.54 |
|  | M | -1.58 | 1.34 | -2.08 – -1.07 | -4.06 | 1.48 | 1.66 |
|  | R | -1.32 | 1.22 | -1.78 – -0.87 | -3.19 | 1.98 | 2.00 |
| Mean amplitude 700-900ms | F | -0.37 | 1.19 | -0.81 – 0.07 | -2.81 | 1.76 | 1.84 |
|  | C | -0.84 | 1.19 | -1.28 – -0.39 | -3.48 | 1.77 | 1.69 |
|  | P | -1.21 | 1.35 | -1.72 – -0.71 | -3.78 | 1.14 | 2.27 |
|  | L | -0.69 | 1.12 | -1.11 – -0.27 | -2.68 | 1.40 | 1.82 |
|  | M | -0.86 | 1.29 | -1.34 – -0.37 | -3.36 | 1.47 | 2.32 |
|  | R | -0.87 | 1.17 | -1.31 – -0.43 | -2.95 | 1.44 | 1.92 |
| Onset latency N400 | F | 462.73 | 73.10 | 435.44 – 490.03 | 357.33 | 656.67 | 109.83 |
|  | C | 512.89 | 69.68 | 486.87 – 538.91 | 399.33 | 693.33 | 96.00 |
|  | P | 528.31 | 57.63 | 506.79 – 549.83 | 422.67 | 634.67 | 75.00 |
|  | L | 485.76 | 64.42 | 461.70 – 509.81 | 364.67 | 657.33 | 87.17 |
|  | M | 498.38 | 61.82 | 475.29 – 521.46 | 422.67 | 634.00 | 103.17 |
|  | R | 519.80 | 85.56 | 487.85 – 551.75 | 413.33 | 852.00 | 90.33 |

Abbreviations: F = frontal, C = central, P = parietal, L = left, M = midline, R = right, SD = standard deviation, CI = confidence interval, Min = minimum, Max = maximum, IQR = interquartile range.

**Table 26.** The descriptive statistics of the mean amplitudes (in μV) and onset latencies (in ms) of the difference waves (incorrect – correct condition) elicited by the semantic anomaly paradigm for the control group at the frontal, central, parietal, left, midline, and right electrode sites.

|  | Electrode | Mean | SD | 95% CI | Min | Max | IQR |
| --- | --- | --- | --- | --- | --- | --- | --- |
| Mean amplitude 300-500ms | F | -0.62 | 1.27 | -1.09 – -0.15 | -2.93 | 1.84 | 2.09 |
|  | C | -1.44 | 1.11 | -1.86 – -1.03 | -4.23 | 0.45 | 1.25 |
|  | P | -1.43 | 0.95 | -1.78 – -1.07 | -3.46 | 0.26 | 1.30 |
|  | L | -0.90 | 0.91 | -1.24 – -0.56 | -2.70 | 0.81 | 1.20 |
|  | M | -1.27 | 1.02 | -1.65 – -0.89 | -3.39 | 0.61 | 1.47 |
|  | R | -1.33 | 0.93 | -1.67 – -0.98 | -3.37 | 0.07 | 1.71 |
| Mean amplitude 500-700ms | F | 0.70 | 1.86 | 0.00 – 1.39 | -3.50 | 4.06 | 2.25 |
|  | C | 0.51 | 1.74 | -0.14 – 1.16 | -5.54 | 4.13 | 1.04 |
|  | P | 0.72 | 1.48 | 0.17 – 1.27 | -2.47 | 4.37 | 1.21 |
|  | L | 0.92 | 1.57 | 0.33 – 1.50 | -3.27 | 4.66 | 1.74 |
|  | M | 0.87 | 1.66 | 0.25 – 1.49 | -4.00 | 4.38 | 1.19 |
|  | R | 0.14 | 1.46 | -0.40 – 0.69 | -3.96 | 3.52 | 1.33 |
| Mean amplitude 700-900ms | F | 1.04 | 1.57 | 0.45 – 1.63 | -1.90 | 4.31 | 2.43 |
|  | C | 1.38 | 1.82 | 0.70 – 2.06 | -3.64 | 4.49 | 2.10 |
|  | P | 1.57 | 1.75 | 0.92 – 2.23 | -1.24 | 6.44 | 2.50 |
|  | L | 1.47 | 1.59 | 0.88 – 2.07 | -2.01 | 4.26 | 2.06 |
|  | M | 1.53 | 1.81 | 0.85 – 2.20 | -2.36 | 5.57 | 2.42 |
|  | R | 0.99 | 1.54 | 0.42 – 1.57 | -2.41 | 4.09 | 2.47 |
| Mean amplitude 900-1100ms | F | 0.39 | 1.49 | -0.17 – 0.95 | -4.84 | 2.46 | 2.25 |
|  | C | 0.44 | 2.01 | -0.31 – 1.19 | -6.13 | 3.14 | 2.61 |
|  | P | 0.51 | 1.68 | -0.12 – 1.13 | -2.69 | 3.89 | 1.99 |
|  | L | 0.53 | 1.63 | -0.08 – 1.13 | -3.94 | 2.95 | 2.10 |
|  | M | 0.49 | 1.80 | -0.18 – 1.16 | -4.74 | 3.35 | 2.17 |
|  | R | 0.32 | 1.56 | -0.26 – 0.90 | -4.53 | 2.81 | 2.46 |
| Onset latency N400 | F | 425.31 | 106.34 | 385.60 – 465.02 | 314.67 | 783.33 | 69.83 |
|  | C | 403.36 | 48.45 | 385.26 – 421.45 | 344.67 | 567.33 | 56.00 |
|  | P | 399.07 | 48.65 | 380.90 – 417.23 | 342.00 | 584.67 | 44.83 |
|  | L | 400.84 | 47.52 | 383.10 – 418.59 | 338.67 | 513.33 | 71.33 |
|  | M | 405.38 | 62.36 | 382.09 – 428.66 | 340.00 | 611.33 | 75.50 |
|  | R | 421.51 | 57.57 | 400.01 – 443.01 | 346.00 | 590.67 | 61.67 |
| Onset latency  LPC | F | 698.24 | 92.99 | 663.52 – 732.97 | 548.67 | 933.33 | 135.67 |
|  | C | 685.96 | 81.93 | 655.36 – 716.55 | 502.00 | 896.00 | 103.33 |
|  | P | 689.11 | 79.50 | 659.42 – 718.80 | 537.33 | 873.33 | 122.17 |
|  | L | 685.42 | 78.85 | 655.98 – 714.87 | 564.67 | 898.67 | 107.83 |
|  | M | 681.02 | 74.46 | 653.22 – 708.83 | 560.67 | 882.00 | 108.50 |
|  | R | 706.87 | 83.72 | 675.61 – 738.13 | 561.33 | 910.67 | 136.33 |

Abbreviations: F = frontal, C = central, P = parietal, L = left, M = midline, R = right, SD = standard deviation, CI = confidence interval, Min = minimum, Max = maximum, IQR = interquartile range.

**Table 27.** The descriptive statistics of the mean amplitudes (in μV) and onset latencies (in ms) of the difference waves (incorrect – correct condition) for the control group at the frontal, central, parietal, left, midline, and right electrode sites.

| Time window | Electrode | Mean | SD | 95% CI | Min | Max | IQR |
| --- | --- | --- | --- | --- | --- | --- | --- |
| Mean amplitude | |  |  |  |  |  |  |
| 500ms - 750ms | F | 2.01 | 2.86 | 0.95 – 3.08 | -4.06 | 8.98 | 2.29 |
|  | C | 2.34 | 2.85 | 1.28 – 3.40 | -2.97 | 10.48 | 3.15 |
|  | P | 2.47 | 3.24 | 1.26 – 3.68 | -4.37 | 9.72 | 4.69 |
|  | L | 1.71 | 2.52 | 0.77 – 2.65 | -3.23 | 8.59 | 3.12 |
|  | M | 2.84 | 3.09 | 1.69 – 3.99 | -2.78 | 10.87 | 3.57 |
|  | R | 2.28 | 2.65 | 1.29 – 3.27 | -2.51 | 9.72 | 3.29 |
| 750ms - 1000ms | F | 1.94 | 2.78 | 0.91 – 2.98 | -4.27 | 7.42 | 2.79 |
|  | C | 3.19 | 2.97 | 2.08 – 4.29 | -2.55 | 12.16 | 3.58 |
|  | P | 3.60 | 3.11 | 2.44 – 4.76 | -1.69 | 12.94 | 4.45 |
|  | L | 2.11 | 2.55 | 1.16 – 3.07 | -1.82 | 8.83 | 3.27 |
|  | M | 3.58 | 3.16 | 2.40 – 4.76 | -1.75 | 12.50 | 4.30 |
|  | R | 3.04 | 2.57 | 2.08 – 4.00 | -1.30 | 11.20 | 3.08 |
| 1000 - 1250ms | F | 1.20 | 2.30 | 0.34 – 2.06 | -2.07 | 6.72 | 3.40 |
|  | C | 1.91 | 2.79 | 0.86 – 2.95 | -2.63 | 9.73 | 3.69 |
|  | P | 1.89 | 2.79 | 0.85 – 2.94 | -3.14 | 8.82 | 3.71 |
|  | L | 1.12 | 2.42 | 0.22 – 2.02 | -2.56 | 7.07 | 3.37 |
|  | M | 1.99 | 2.93 | 0.89 – 3.08 | -3.45 | 9.46 | 4.11 |
|  | R | 1.89 | 2.33 | 1.02 – 2.76 | -1.10 | 8.74 | 3.02 |
| Onset latency | |  |  |  |  |  |  |
| 500ms - 1250ms | F | 706.73 | 100.77 | 669.10 – 744.36 | 565.33 | 958.00 | 109.33 |
|  | C | 718.71 | 70.97 | 692.21 – 745.21 | 597.33 | 914.00 | 100.67 |
|  | P | 720.89 | 64.12 | 696.95 – 744.83 | 588.67 | 879.33 | 81.83 |
|  | L | 712.69 | 72.59 | 685.58 – 739.80 | 594.00 | 932.00 | 90.67 |
|  | M | 705.69 | 64.00 | 681.79 – 729.59 | 585.33 | 891.33 | 89.50 |
|  | R | 727.96 | 72.54 | 700.87 – 755.04 | 598.00 | 928.00 | 125.33 |

Abbreviations: F = frontal; C = central; P = parietal; L = left; M = midline; R = right; SD = standard deviation; CI = confidence interval; Min = minimum; Max = maximum; IQR = interquartile range.
